# Supplementary figures and images for: Making simulation results reproducible—Survey, guidelines, and examples based on Gradle and Docker
Source: PeerJ Comput Sci. 2019 Dec 9;5:e240. doi: 10.7717/peerj-cs.240 (PMC7924710; doi:10.7717/peerj-cs.240)

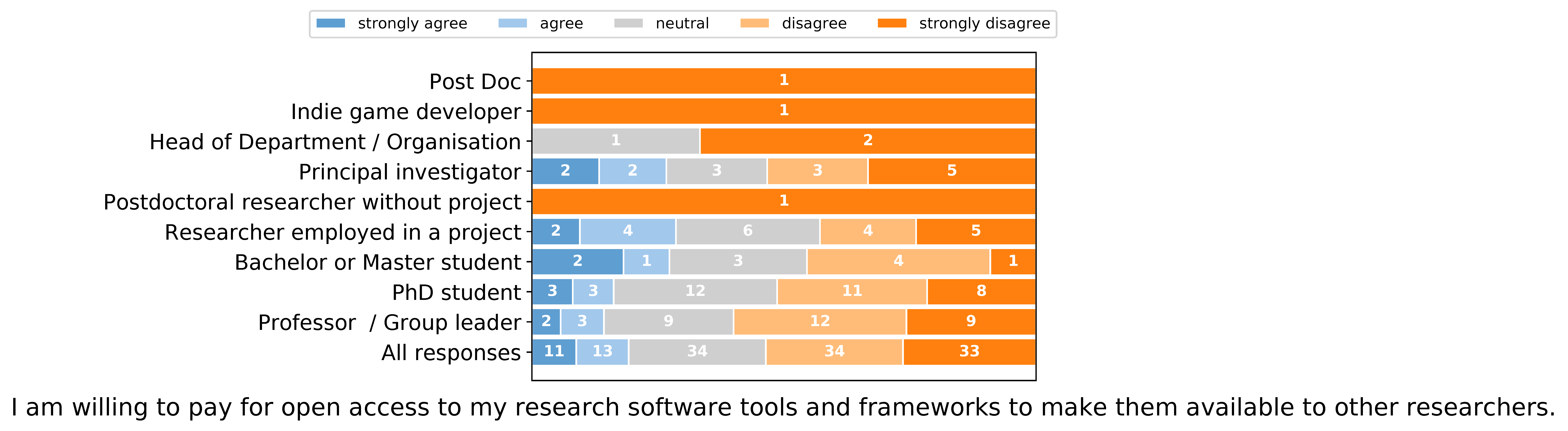

Supplement: Supplemental Information 2 — The answers to each survey question have been evaluated (1) grouped by position, (2) grouped by position, with all groups smaller than a threshold of 10 being summarized in one “other” category, (3) grouped by area of research, (4) grouped by area of research, with all groups smaller than a threshold of 10 being summarized in one “other” category, (5) grouped by research environment, (6) grouped by research environment, with all groups smaller than a threshold of 10 being summarized in one “other” category. [file peerj-cs-05-240-s002.zip › reproducibility-survey-analysis-byposition-question-01.png]

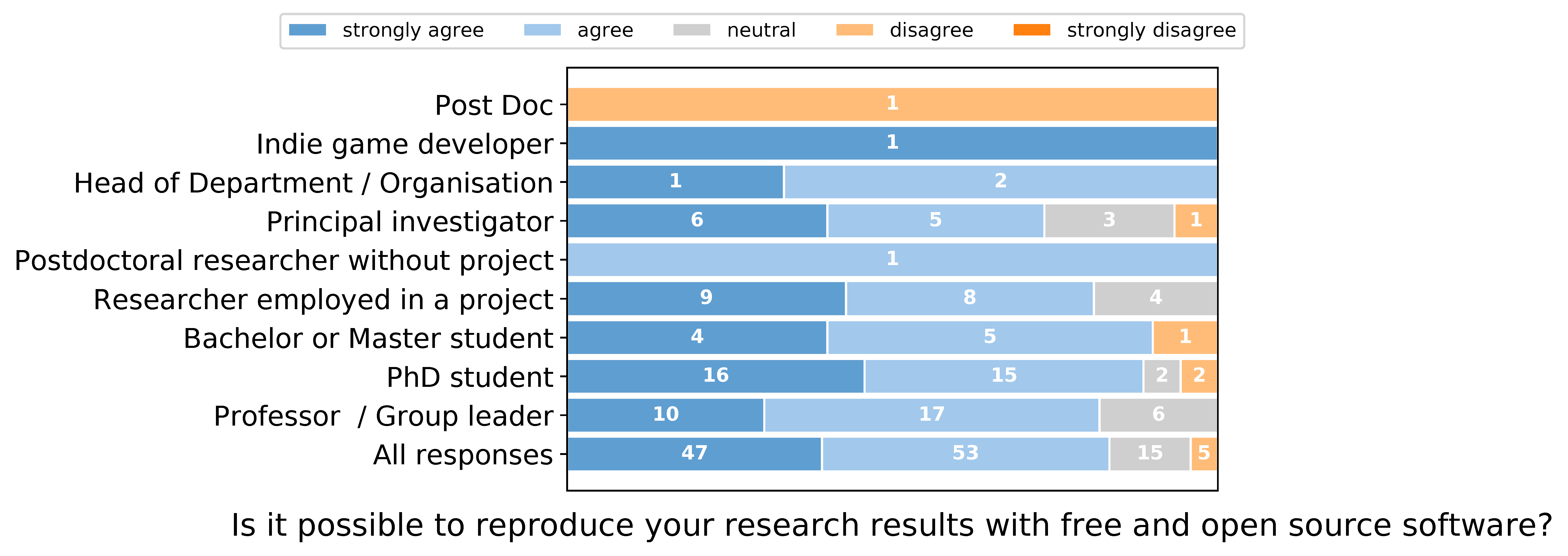

Supplement: Supplemental Information 2 — The answers to each survey question have been evaluated (1) grouped by position, (2) grouped by position, with all groups smaller than a threshold of 10 being summarized in one “other” category, (3) grouped by area of research, (4) grouped by area of research, with all groups smaller than a threshold of 10 being summarized in one “other” category, (5) grouped by research environment, (6) grouped by research environment, with all groups smaller than a threshold of 10 being summarized in one “other” category. [file peerj-cs-05-240-s002.zip › reproducibility-survey-analysis-byposition-question-02.png]

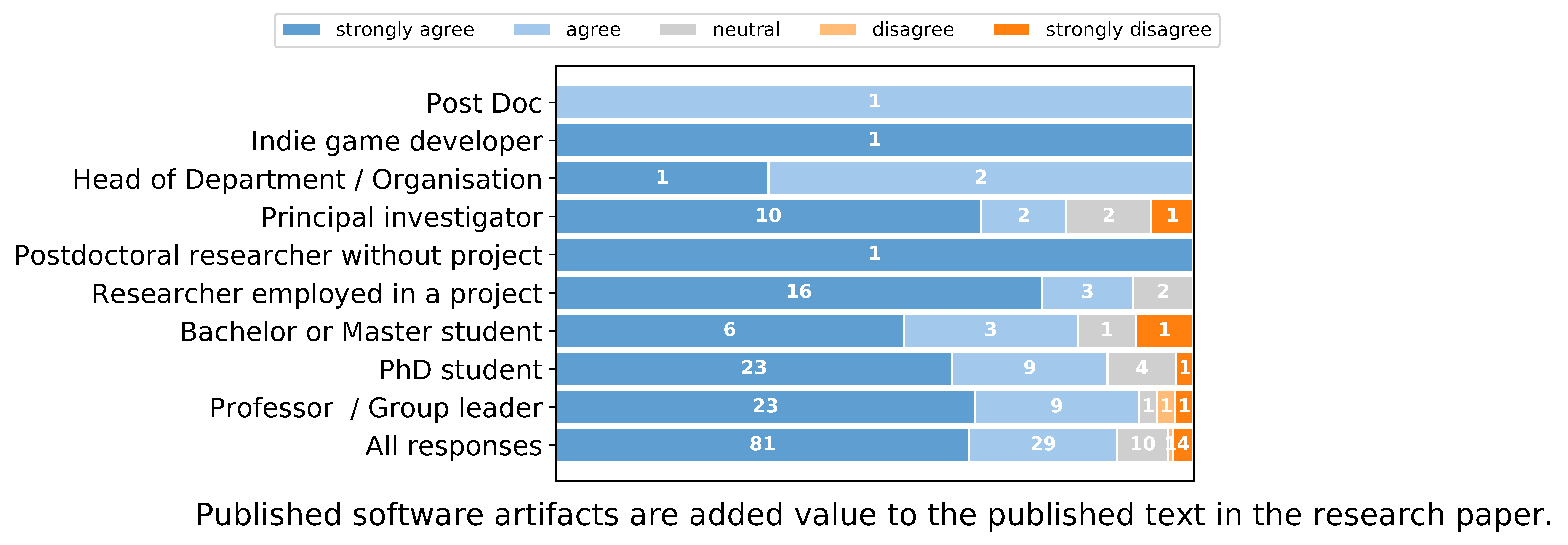

Supplement: Supplemental Information 2 — The answers to each survey question have been evaluated (1) grouped by position, (2) grouped by position, with all groups smaller than a threshold of 10 being summarized in one “other” category, (3) grouped by area of research, (4) grouped by area of research, with all groups smaller than a threshold of 10 being summarized in one “other” category, (5) grouped by research environment, (6) grouped by research environment, with all groups smaller than a threshold of 10 being summarized in one “other” category. [file peerj-cs-05-240-s002.zip › reproducibility-survey-analysis-byposition-question-03.png]

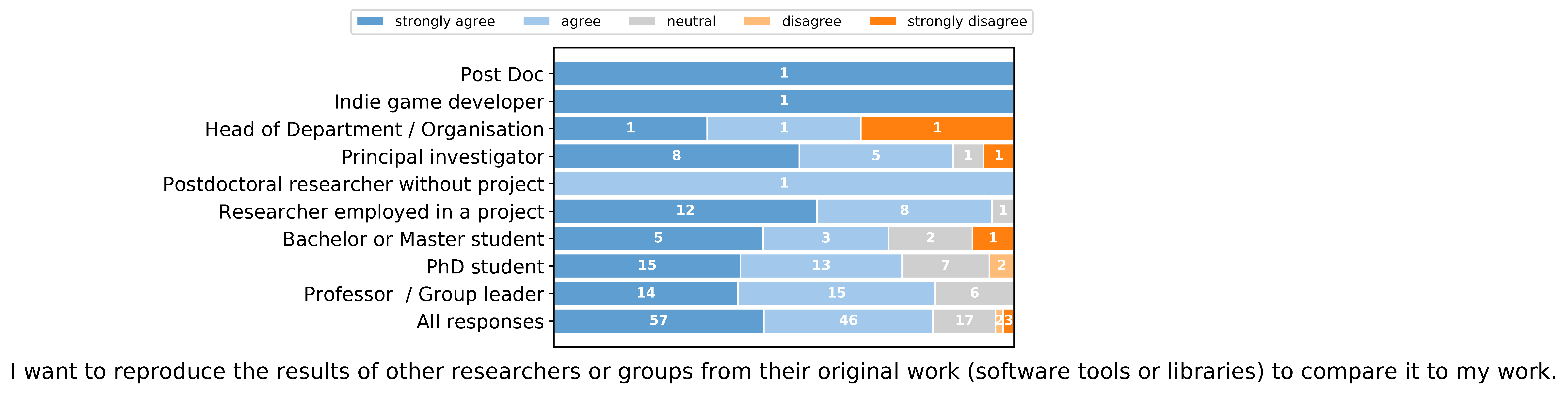

Supplement: Supplemental Information 2 — The answers to each survey question have been evaluated (1) grouped by position, (2) grouped by position, with all groups smaller than a threshold of 10 being summarized in one “other” category, (3) grouped by area of research, (4) grouped by area of research, with all groups smaller than a threshold of 10 being summarized in one “other” category, (5) grouped by research environment, (6) grouped by research environment, with all groups smaller than a threshold of 10 being summarized in one “other” category. [file peerj-cs-05-240-s002.zip › reproducibility-survey-analysis-byposition-question-04.png]

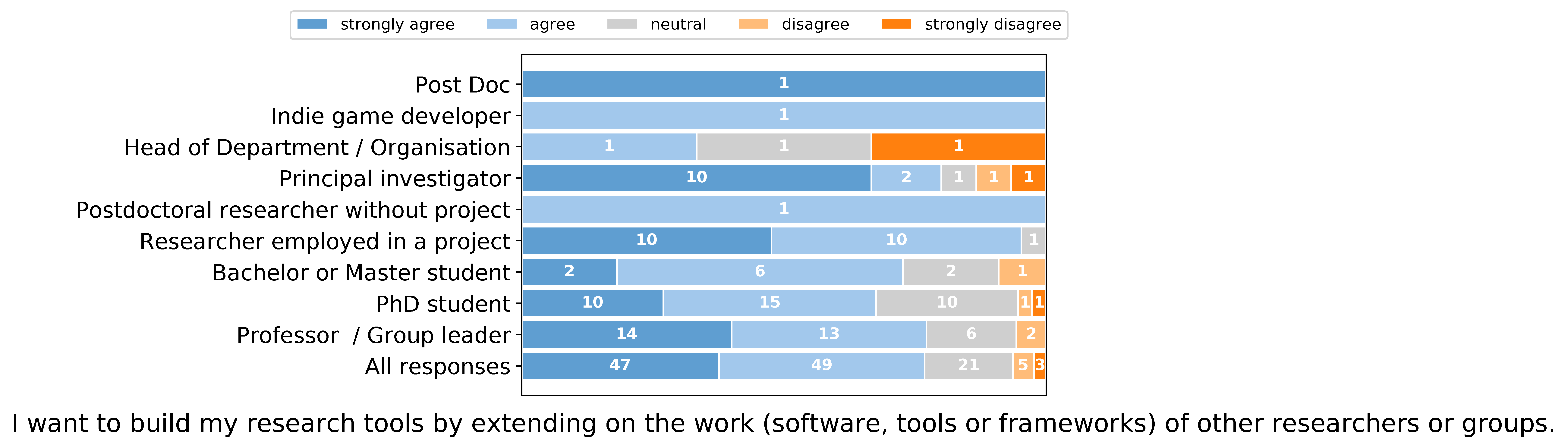

Supplement: Supplemental Information 2 — The answers to each survey question have been evaluated (1) grouped by position, (2) grouped by position, with all groups smaller than a threshold of 10 being summarized in one “other” category, (3) grouped by area of research, (4) grouped by area of research, with all groups smaller than a threshold of 10 being summarized in one “other” category, (5) grouped by research environment, (6) grouped by research environment, with all groups smaller than a threshold of 10 being summarized in one “other” category. [file peerj-cs-05-240-s002.zip › reproducibility-survey-analysis-byposition-question-05.png]

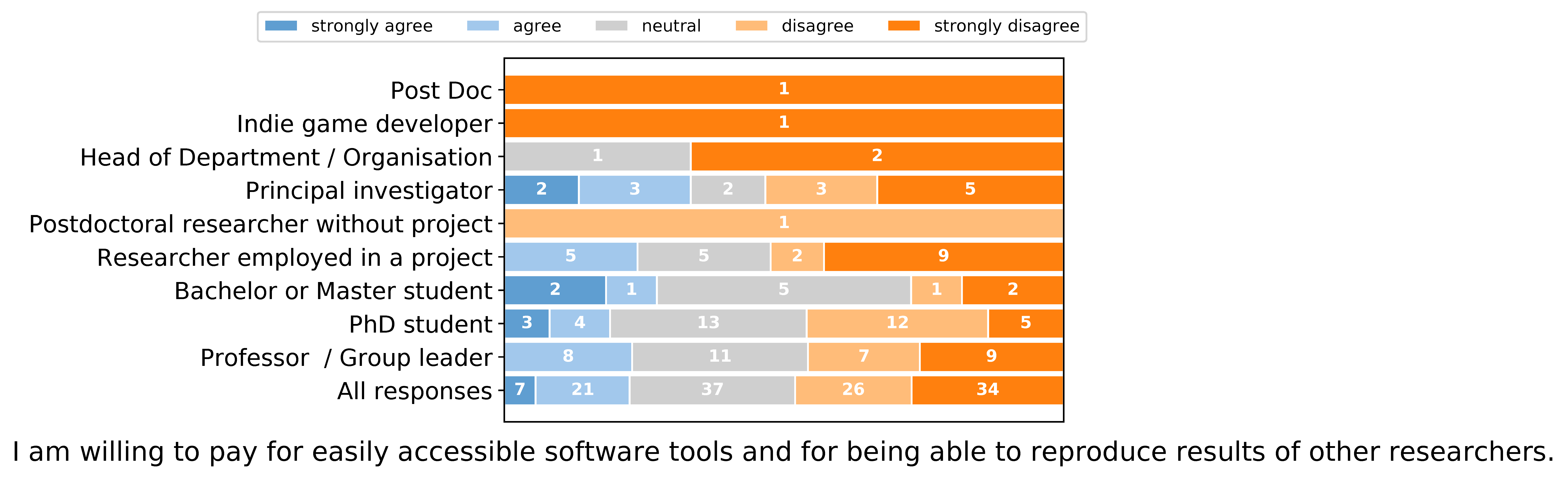

Supplement: Supplemental Information 2 — The answers to each survey question have been evaluated (1) grouped by position, (2) grouped by position, with all groups smaller than a threshold of 10 being summarized in one “other” category, (3) grouped by area of research, (4) grouped by area of research, with all groups smaller than a threshold of 10 being summarized in one “other” category, (5) grouped by research environment, (6) grouped by research environment, with all groups smaller than a threshold of 10 being summarized in one “other” category. [file peerj-cs-05-240-s002.zip › reproducibility-survey-analysis-byposition-question-06.png]

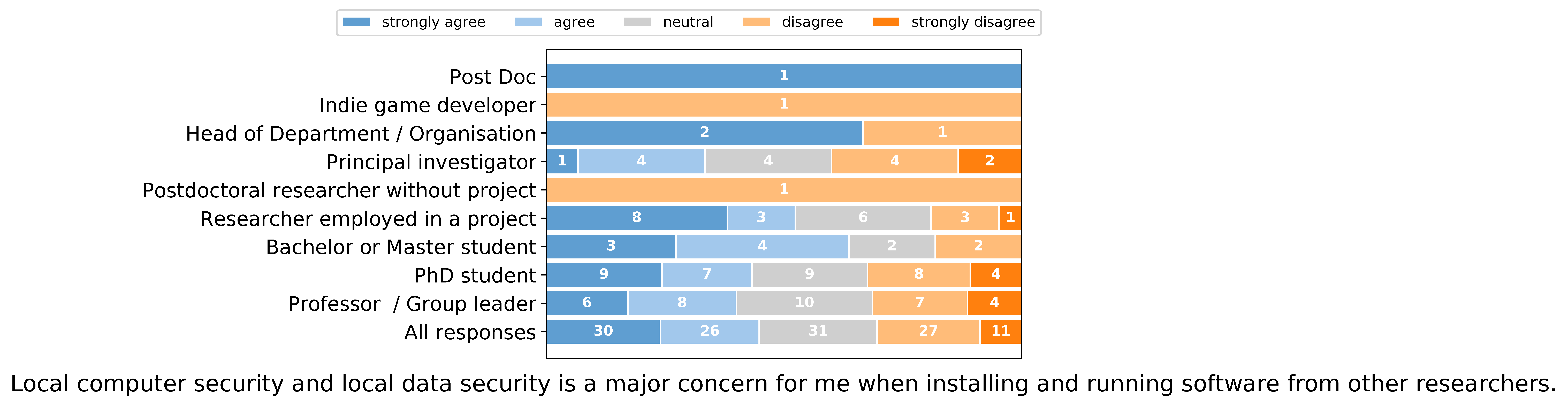

Supplement: Supplemental Information 2 — The answers to each survey question have been evaluated (1) grouped by position, (2) grouped by position, with all groups smaller than a threshold of 10 being summarized in one “other” category, (3) grouped by area of research, (4) grouped by area of research, with all groups smaller than a threshold of 10 being summarized in one “other” category, (5) grouped by research environment, (6) grouped by research environment, with all groups smaller than a threshold of 10 being summarized in one “other” category. [file peerj-cs-05-240-s002.zip › reproducibility-survey-analysis-byposition-question-07.png]

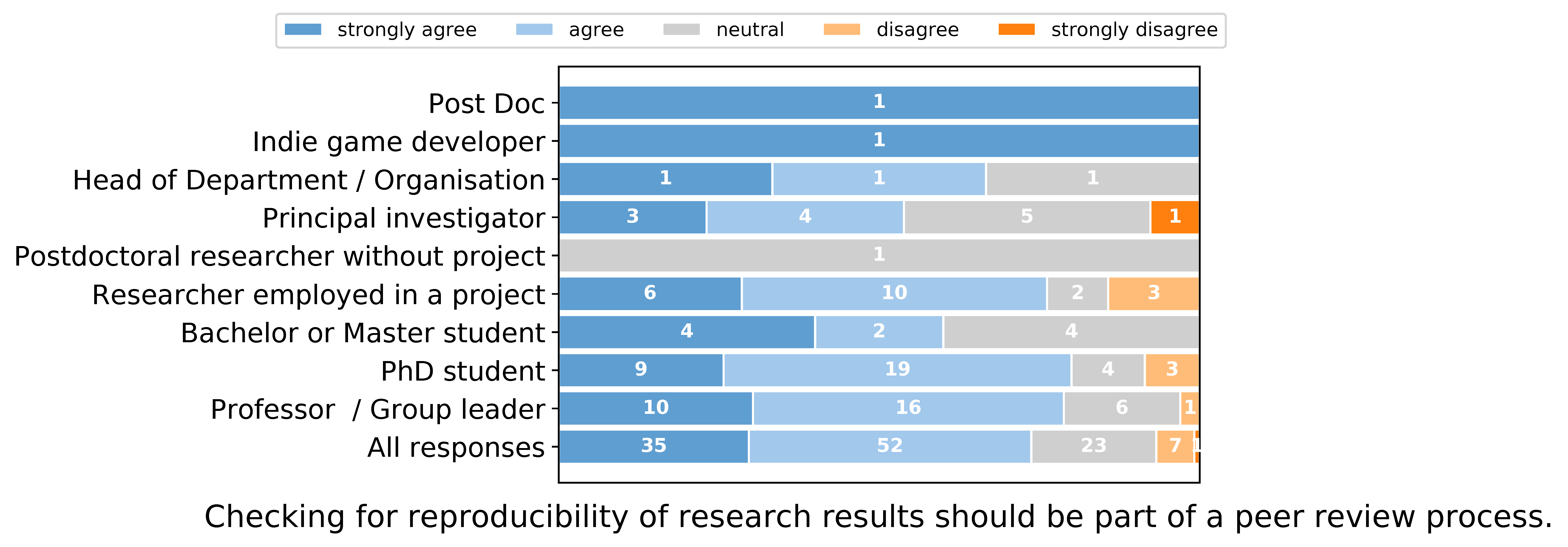

Supplement: Supplemental Information 2 — The answers to each survey question have been evaluated (1) grouped by position, (2) grouped by position, with all groups smaller than a threshold of 10 being summarized in one “other” category, (3) grouped by area of research, (4) grouped by area of research, with all groups smaller than a threshold of 10 being summarized in one “other” category, (5) grouped by research environment, (6) grouped by research environment, with all groups smaller than a threshold of 10 being summarized in one “other” category. [file peerj-cs-05-240-s002.zip › reproducibility-survey-analysis-byposition-question-08.png]

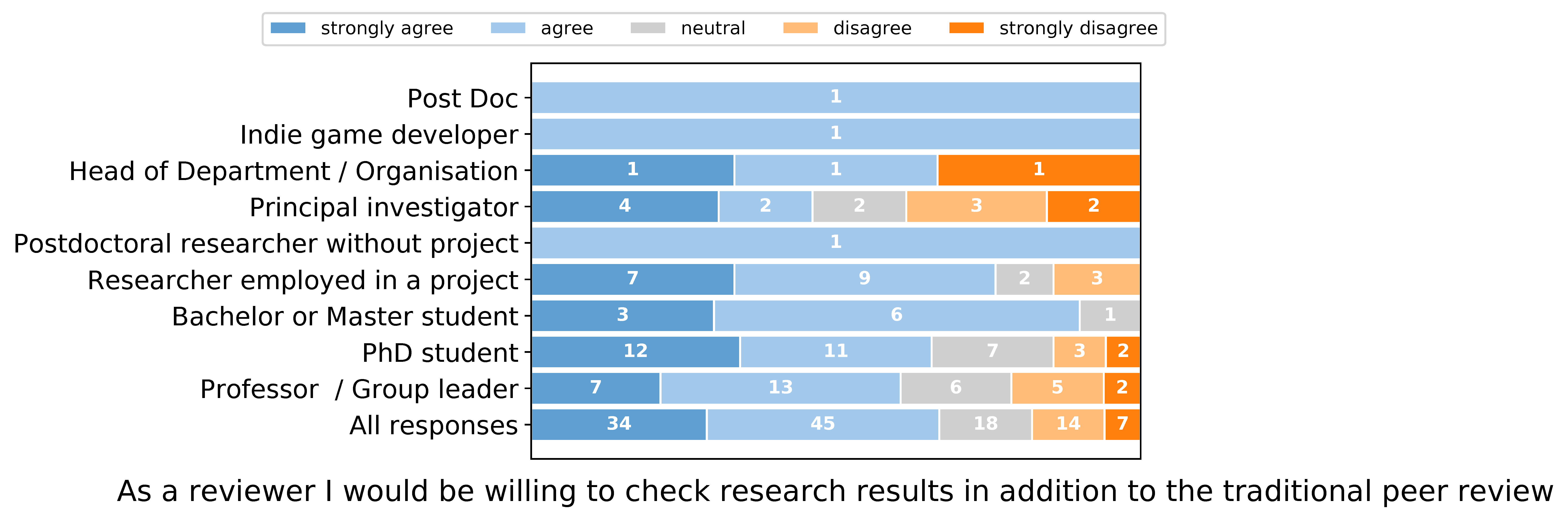

Supplement: Supplemental Information 2 — The answers to each survey question have been evaluated (1) grouped by position, (2) grouped by position, with all groups smaller than a threshold of 10 being summarized in one “other” category, (3) grouped by area of research, (4) grouped by area of research, with all groups smaller than a threshold of 10 being summarized in one “other” category, (5) grouped by research environment, (6) grouped by research environment, with all groups smaller than a threshold of 10 being summarized in one “other” category. [file peerj-cs-05-240-s002.zip › reproducibility-survey-analysis-byposition-question-09.png]

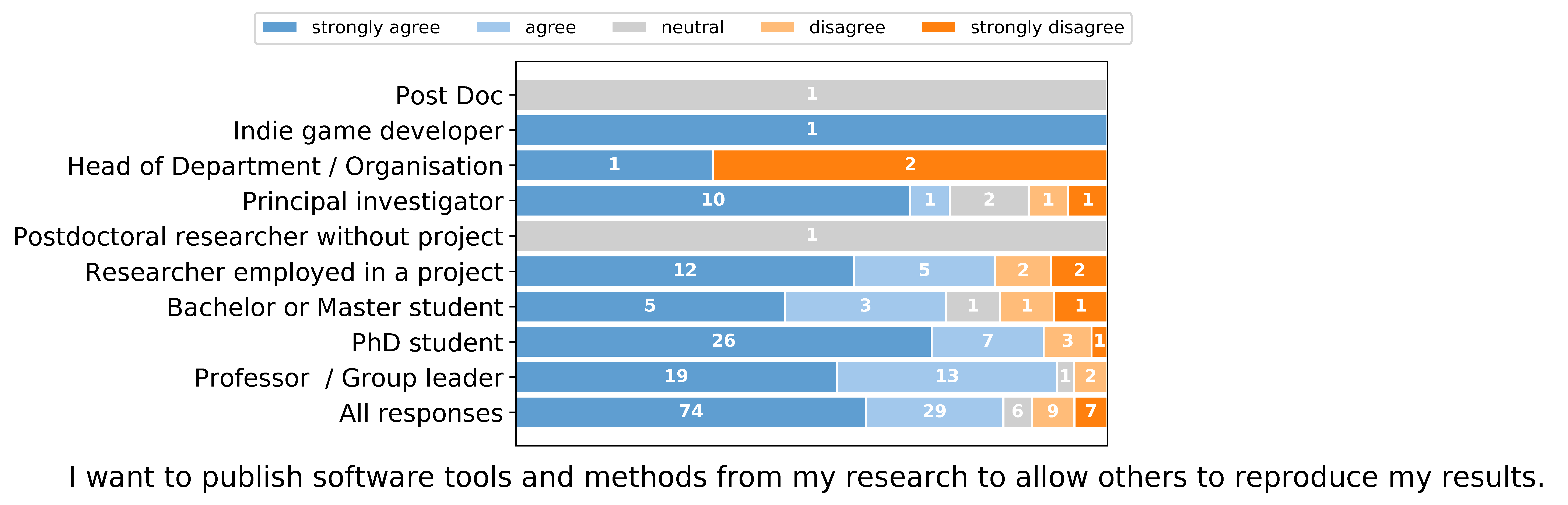

Supplement: Supplemental Information 2 — The answers to each survey question have been evaluated (1) grouped by position, (2) grouped by position, with all groups smaller than a threshold of 10 being summarized in one “other” category, (3) grouped by area of research, (4) grouped by area of research, with all groups smaller than a threshold of 10 being summarized in one “other” category, (5) grouped by research environment, (6) grouped by research environment, with all groups smaller than a threshold of 10 being summarized in one “other” category. [file peerj-cs-05-240-s002.zip › reproducibility-survey-analysis-byposition-question-10.png]

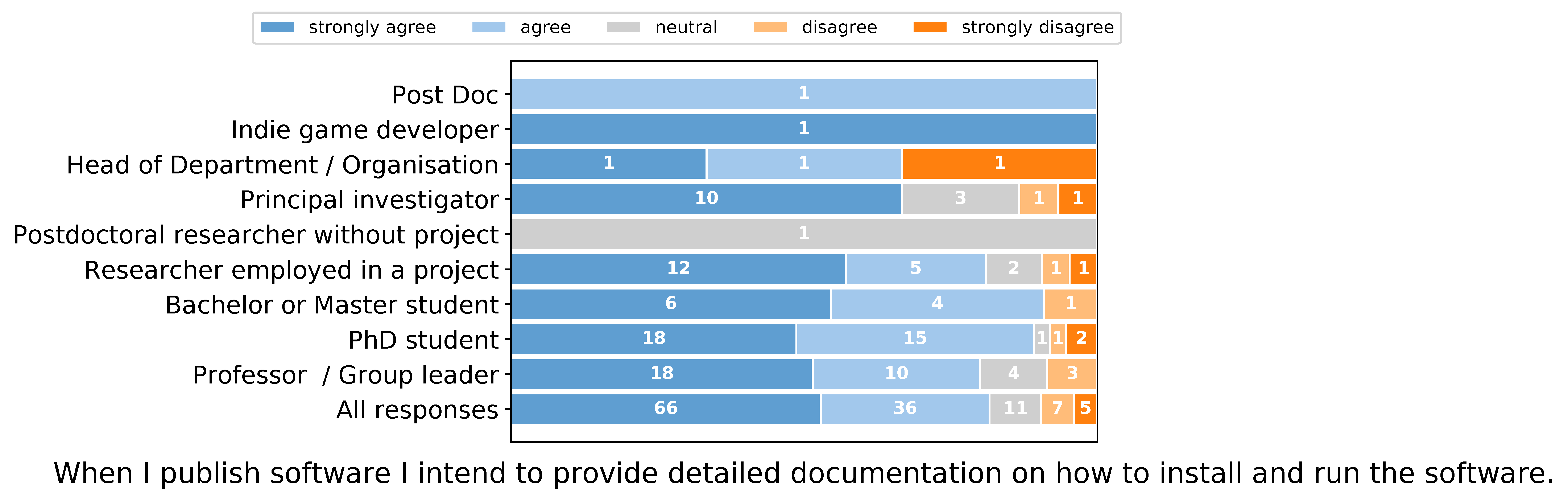

Supplement: Supplemental Information 2 — The answers to each survey question have been evaluated (1) grouped by position, (2) grouped by position, with all groups smaller than a threshold of 10 being summarized in one “other” category, (3) grouped by area of research, (4) grouped by area of research, with all groups smaller than a threshold of 10 being summarized in one “other” category, (5) grouped by research environment, (6) grouped by research environment, with all groups smaller than a threshold of 10 being summarized in one “other” category. [file peerj-cs-05-240-s002.zip › reproducibility-survey-analysis-byposition-question-11.png]

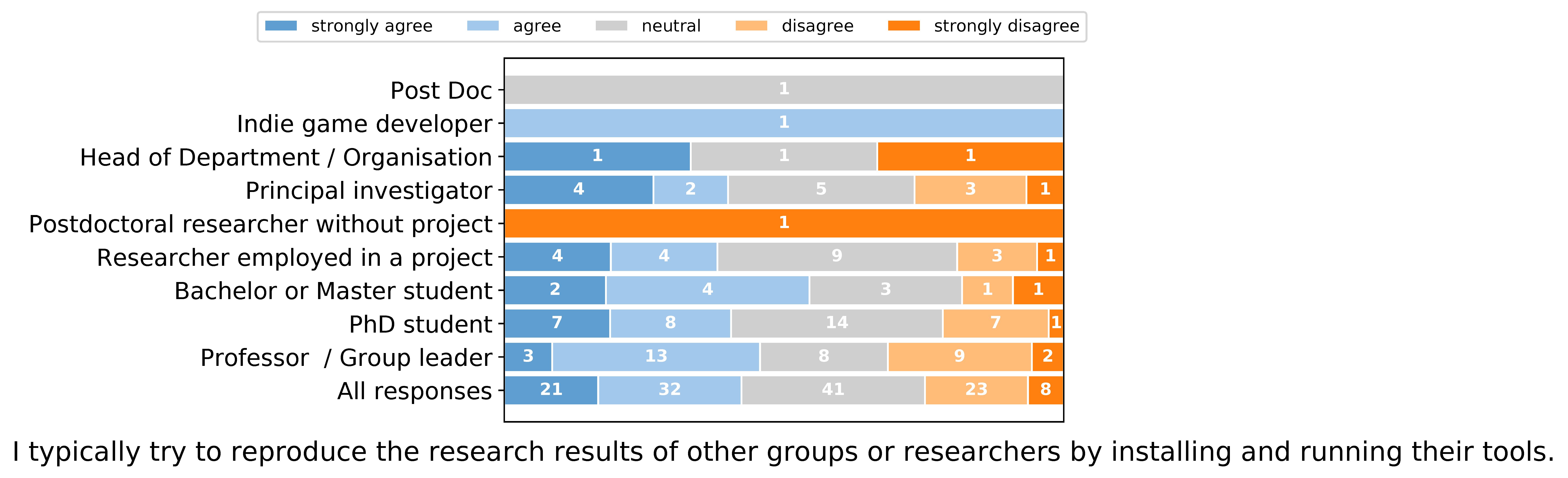

Supplement: Supplemental Information 2 — The answers to each survey question have been evaluated (1) grouped by position, (2) grouped by position, with all groups smaller than a threshold of 10 being summarized in one “other” category, (3) grouped by area of research, (4) grouped by area of research, with all groups smaller than a threshold of 10 being summarized in one “other” category, (5) grouped by research environment, (6) grouped by research environment, with all groups smaller than a threshold of 10 being summarized in one “other” category. [file peerj-cs-05-240-s002.zip › reproducibility-survey-analysis-byposition-question-12.png]

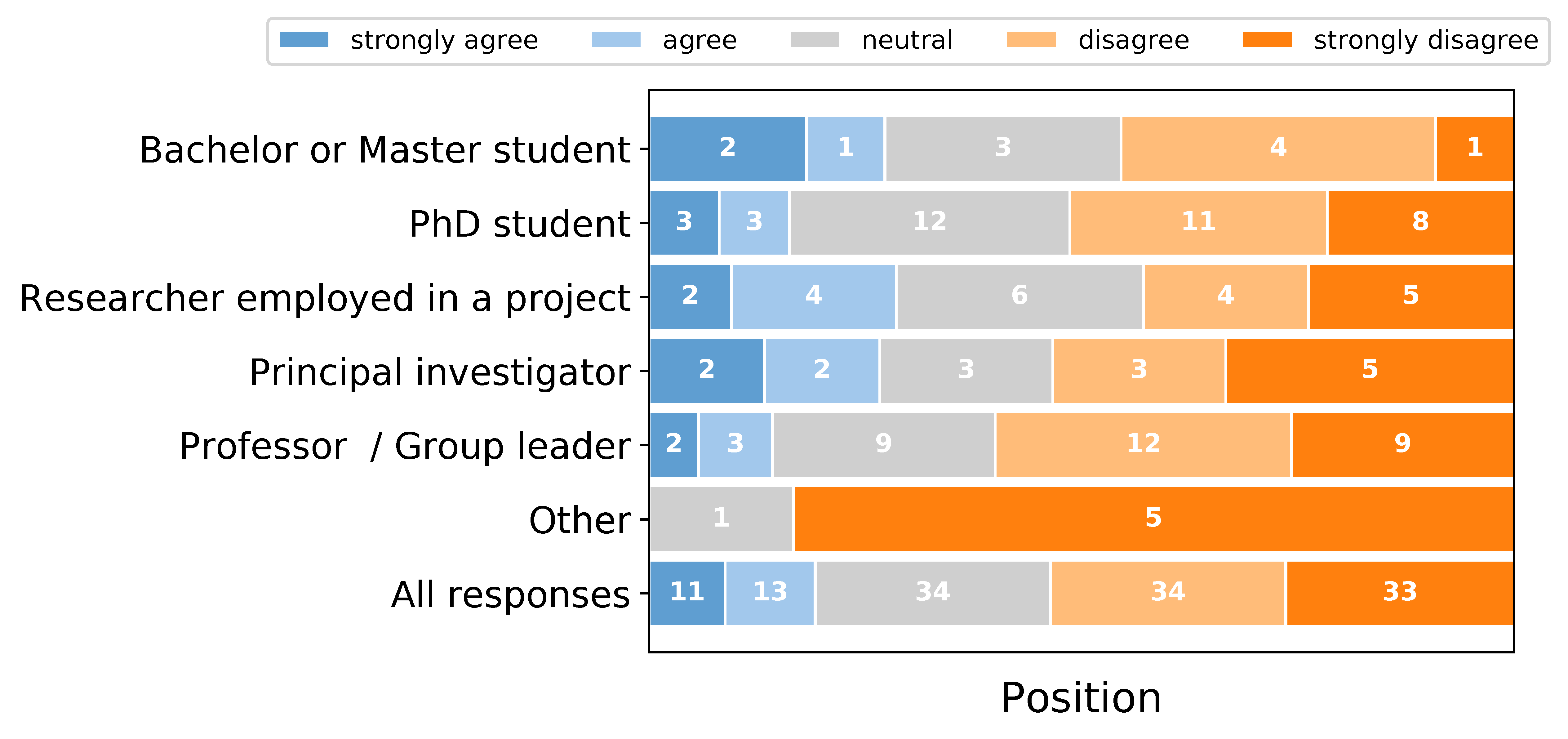

Supplement: Supplemental Information 2 — The answers to each survey question have been evaluated (1) grouped by position, (2) grouped by position, with all groups smaller than a threshold of 10 being summarized in one “other” category, (3) grouped by area of research, (4) grouped by area of research, with all groups smaller than a threshold of 10 being summarized in one “other” category, (5) grouped by research environment, (6) grouped by research environment, with all groups smaller than a threshold of 10 being summarized in one “other” category. [file peerj-cs-05-240-s002.zip › reproducibility-survey-analysis-bypositionthreshold-question-01.png]

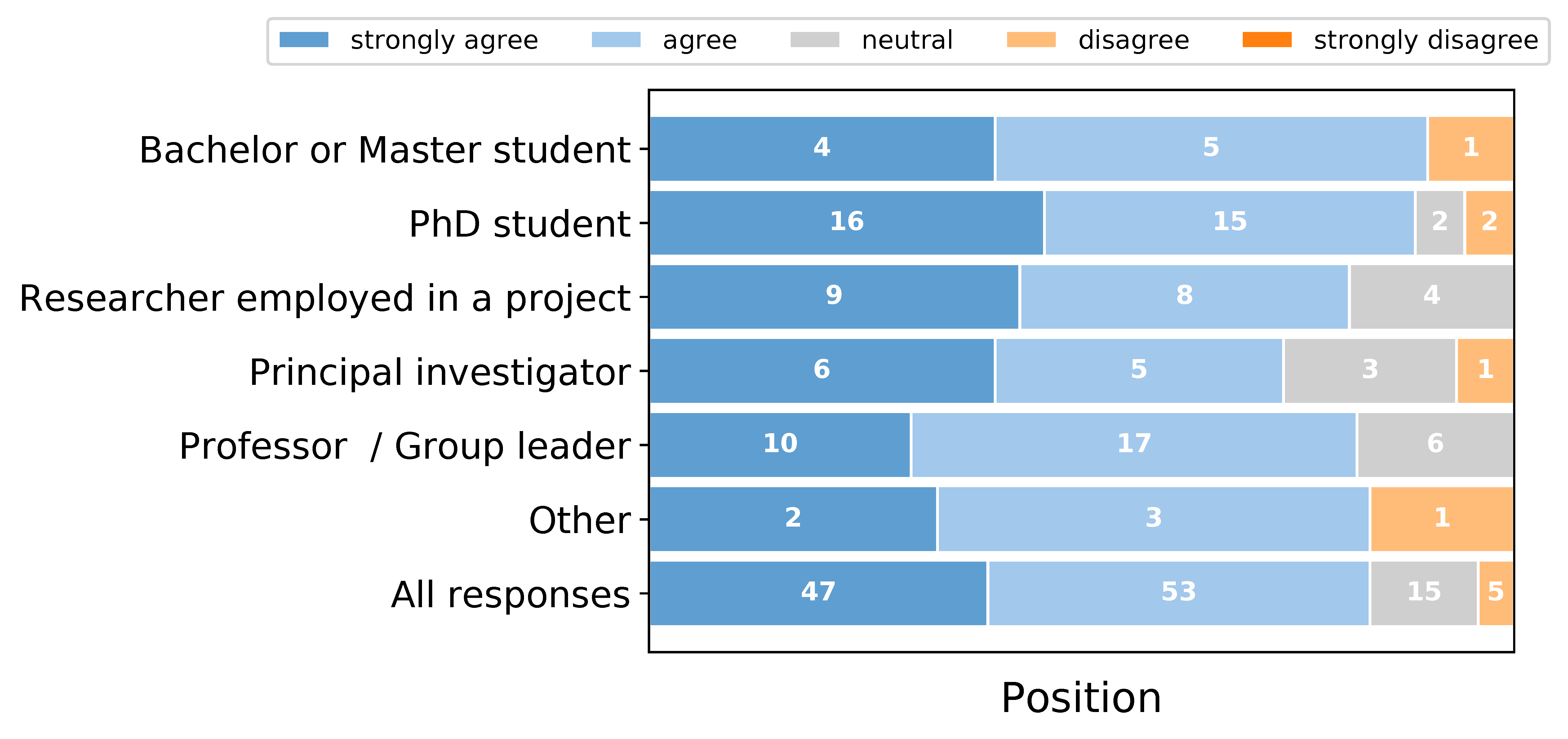

Supplement: Supplemental Information 2 — The answers to each survey question have been evaluated (1) grouped by position, (2) grouped by position, with all groups smaller than a threshold of 10 being summarized in one “other” category, (3) grouped by area of research, (4) grouped by area of research, with all groups smaller than a threshold of 10 being summarized in one “other” category, (5) grouped by research environment, (6) grouped by research environment, with all groups smaller than a threshold of 10 being summarized in one “other” category. [file peerj-cs-05-240-s002.zip › reproducibility-survey-analysis-bypositionthreshold-question-02.png]

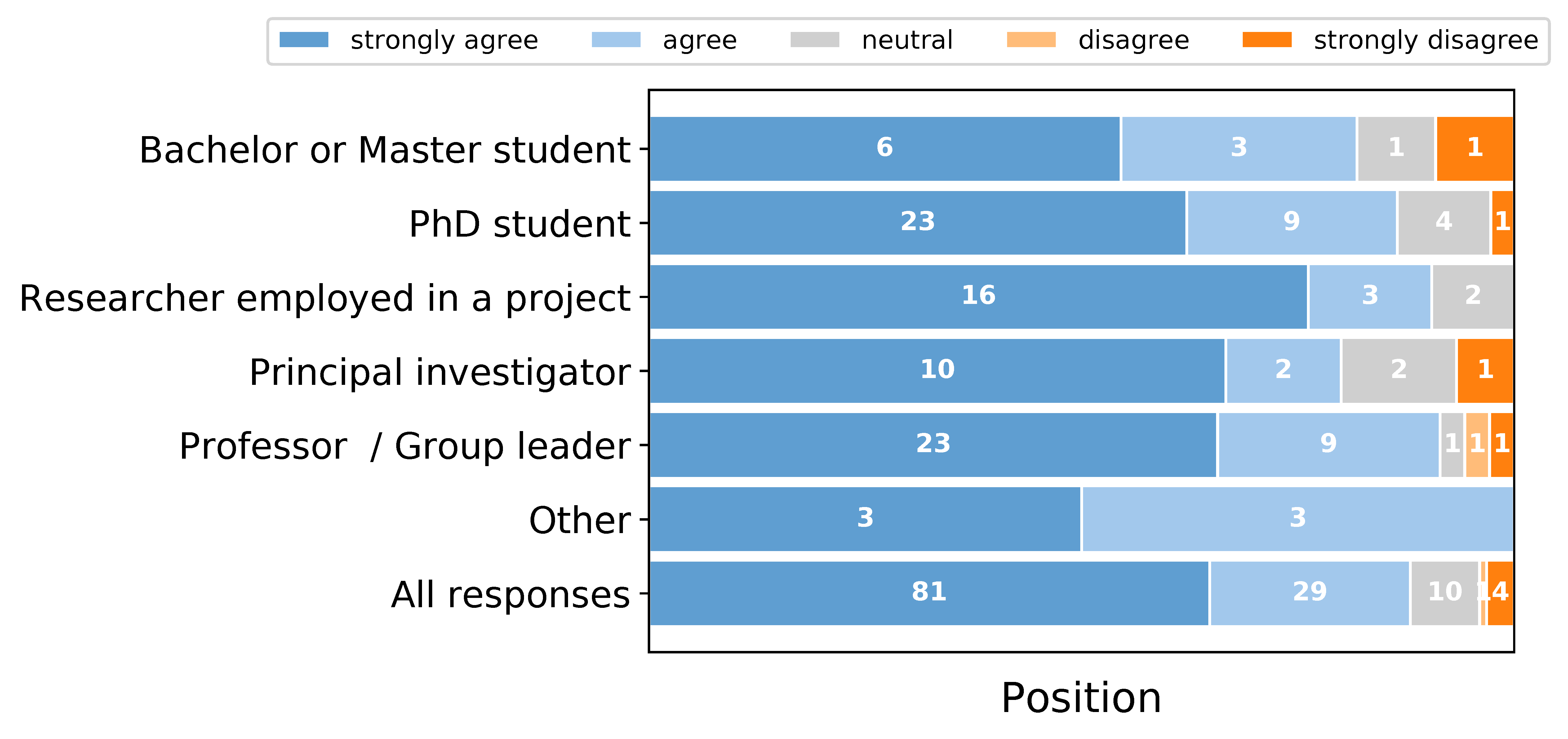

Supplement: Supplemental Information 2 — The answers to each survey question have been evaluated (1) grouped by position, (2) grouped by position, with all groups smaller than a threshold of 10 being summarized in one “other” category, (3) grouped by area of research, (4) grouped by area of research, with all groups smaller than a threshold of 10 being summarized in one “other” category, (5) grouped by research environment, (6) grouped by research environment, with all groups smaller than a threshold of 10 being summarized in one “other” category. [file peerj-cs-05-240-s002.zip › reproducibility-survey-analysis-bypositionthreshold-question-03.png]

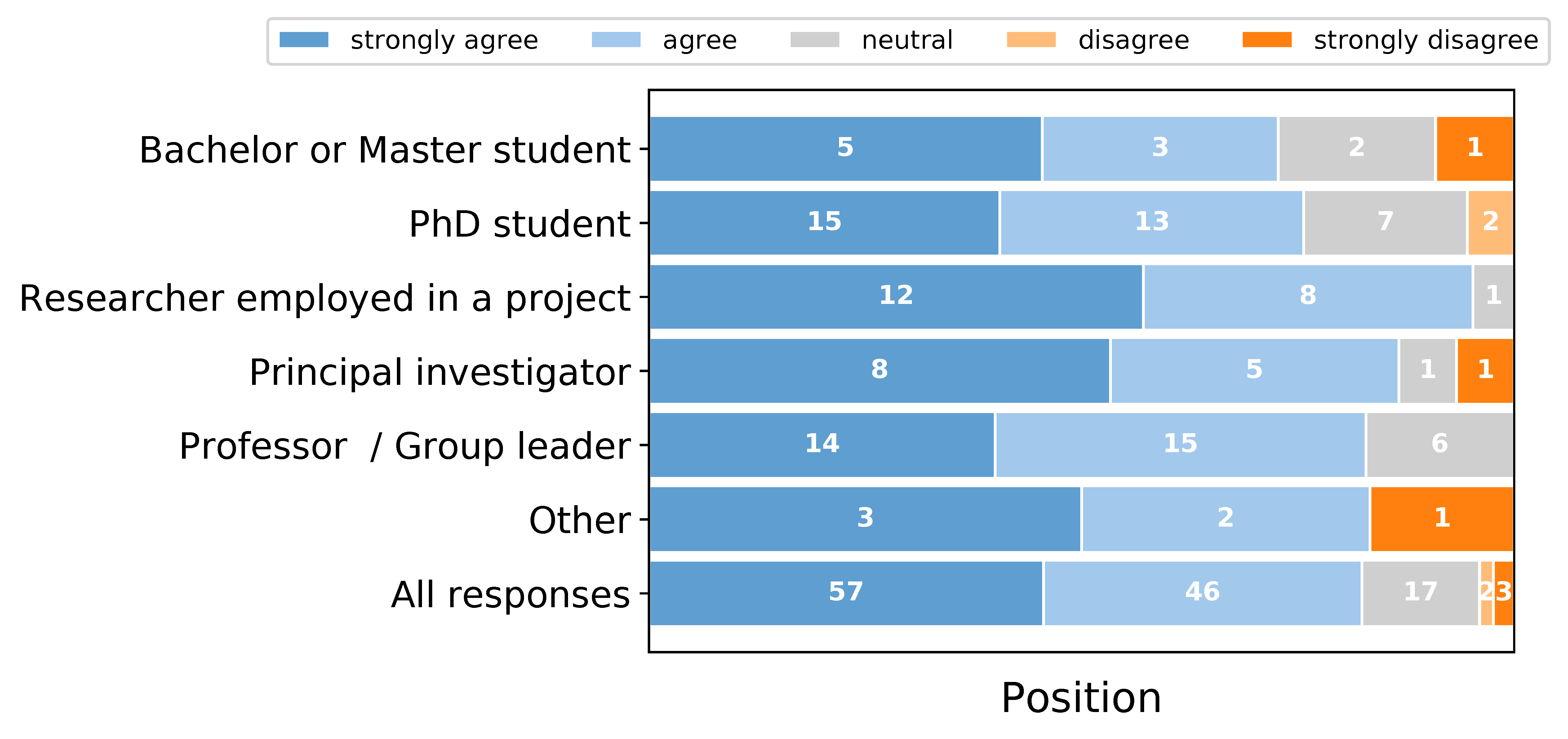

Supplement: Supplemental Information 2 — The answers to each survey question have been evaluated (1) grouped by position, (2) grouped by position, with all groups smaller than a threshold of 10 being summarized in one “other” category, (3) grouped by area of research, (4) grouped by area of research, with all groups smaller than a threshold of 10 being summarized in one “other” category, (5) grouped by research environment, (6) grouped by research environment, with all groups smaller than a threshold of 10 being summarized in one “other” category. [file peerj-cs-05-240-s002.zip › reproducibility-survey-analysis-bypositionthreshold-question-04.png]

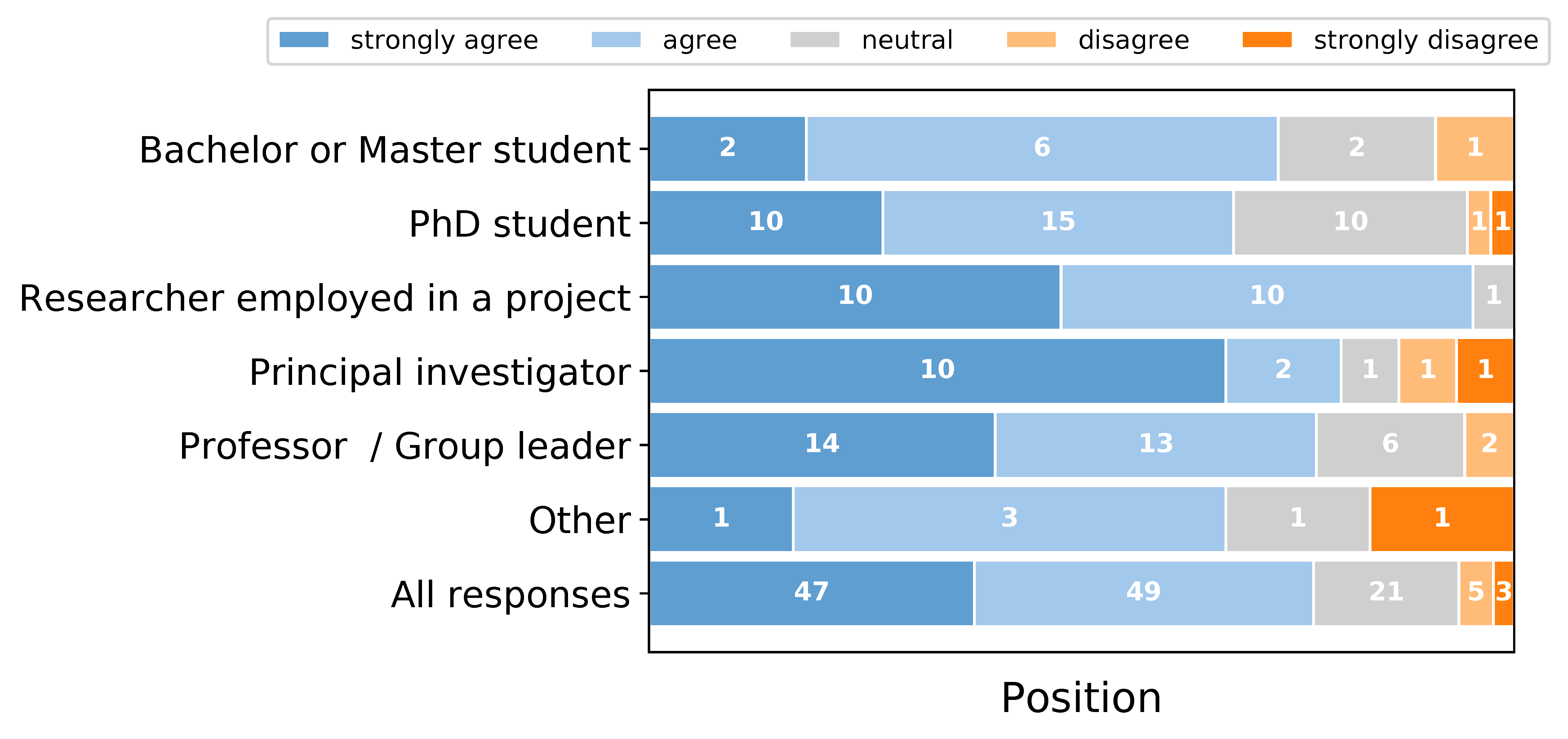

Supplement: Supplemental Information 2 — The answers to each survey question have been evaluated (1) grouped by position, (2) grouped by position, with all groups smaller than a threshold of 10 being summarized in one “other” category, (3) grouped by area of research, (4) grouped by area of research, with all groups smaller than a threshold of 10 being summarized in one “other” category, (5) grouped by research environment, (6) grouped by research environment, with all groups smaller than a threshold of 10 being summarized in one “other” category. [file peerj-cs-05-240-s002.zip › reproducibility-survey-analysis-bypositionthreshold-question-05.png]

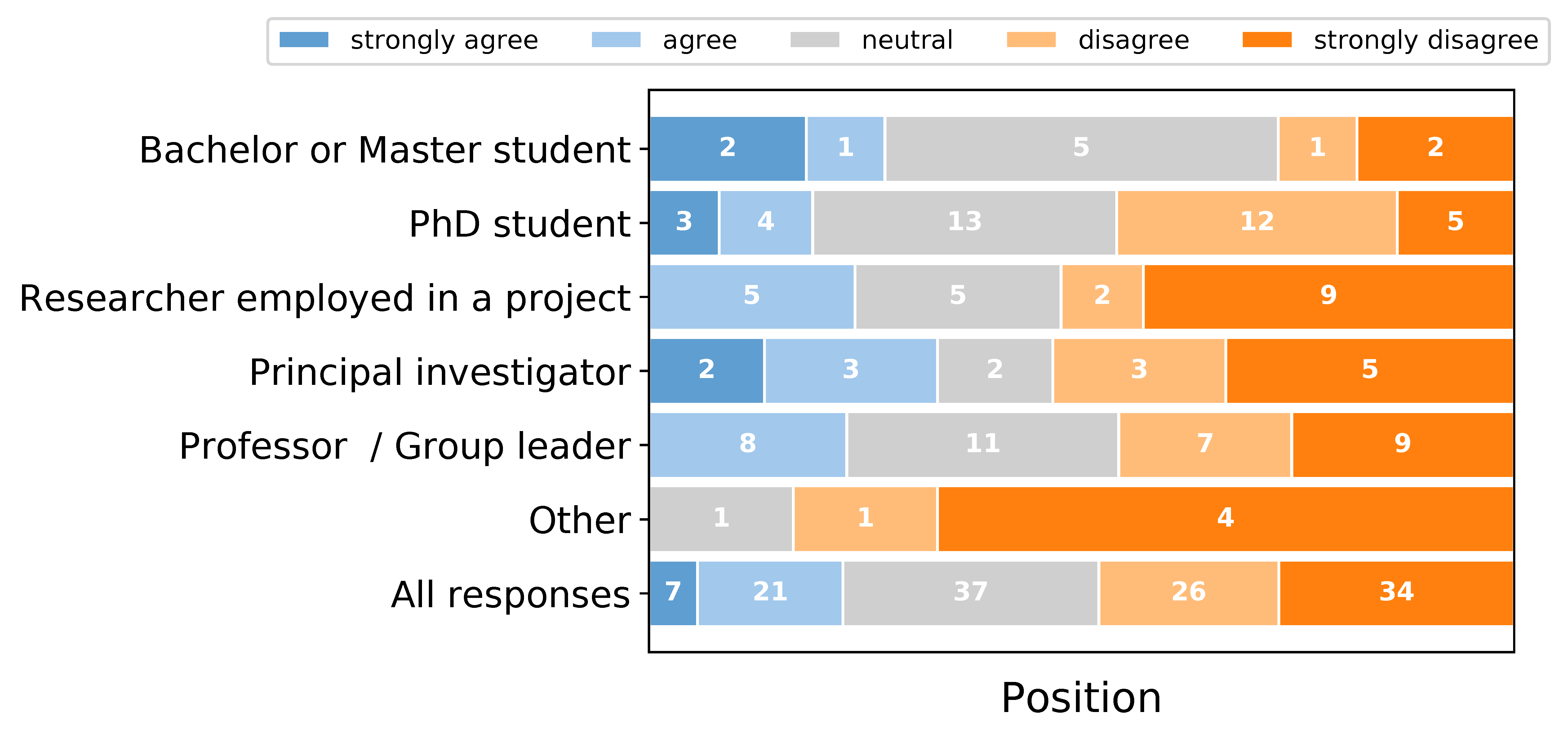

Supplement: Supplemental Information 2 — The answers to each survey question have been evaluated (1) grouped by position, (2) grouped by position, with all groups smaller than a threshold of 10 being summarized in one “other” category, (3) grouped by area of research, (4) grouped by area of research, with all groups smaller than a threshold of 10 being summarized in one “other” category, (5) grouped by research environment, (6) grouped by research environment, with all groups smaller than a threshold of 10 being summarized in one “other” category. [file peerj-cs-05-240-s002.zip › reproducibility-survey-analysis-bypositionthreshold-question-06.png]

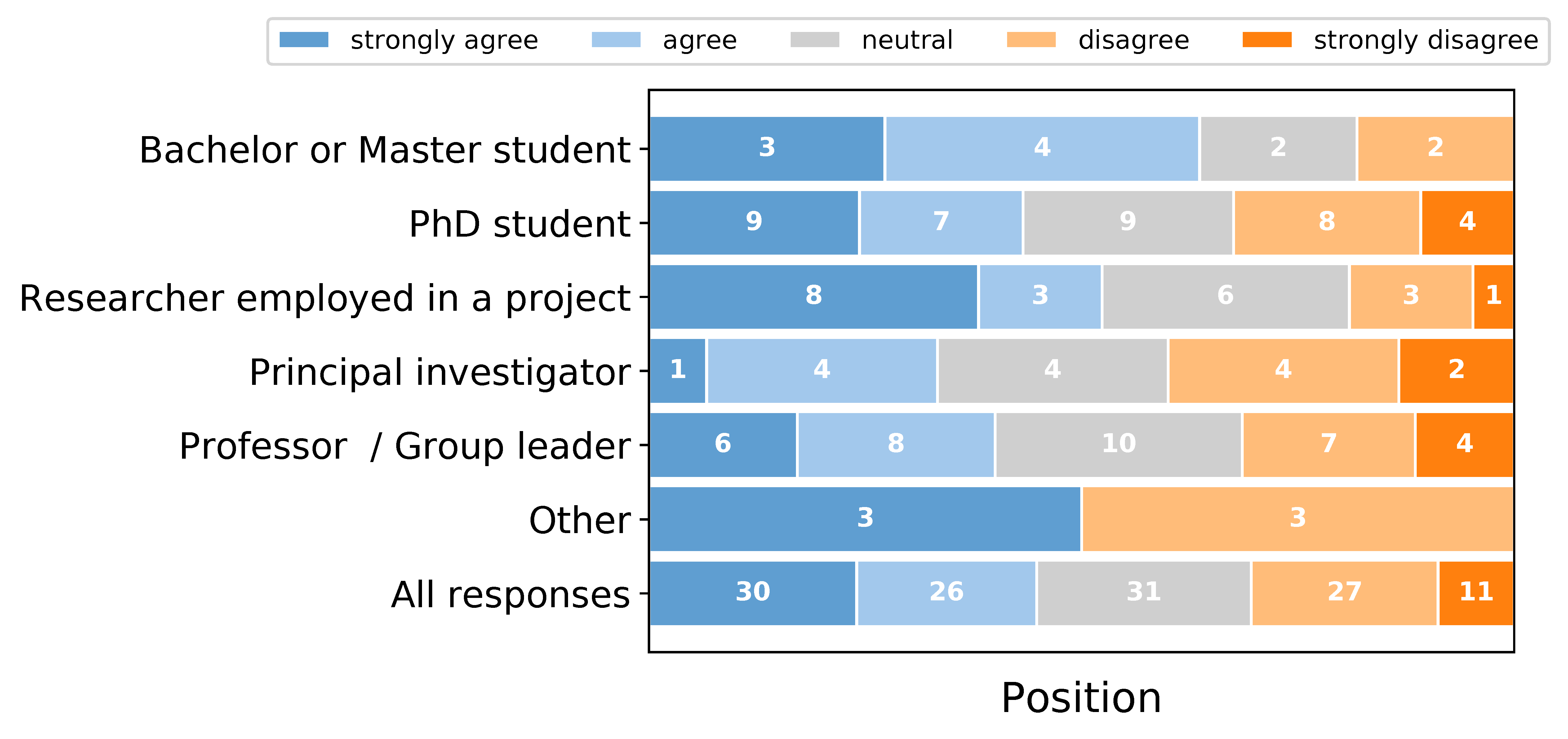

Supplement: Supplemental Information 2 — The answers to each survey question have been evaluated (1) grouped by position, (2) grouped by position, with all groups smaller than a threshold of 10 being summarized in one “other” category, (3) grouped by area of research, (4) grouped by area of research, with all groups smaller than a threshold of 10 being summarized in one “other” category, (5) grouped by research environment, (6) grouped by research environment, with all groups smaller than a threshold of 10 being summarized in one “other” category. [file peerj-cs-05-240-s002.zip › reproducibility-survey-analysis-bypositionthreshold-question-07.png]

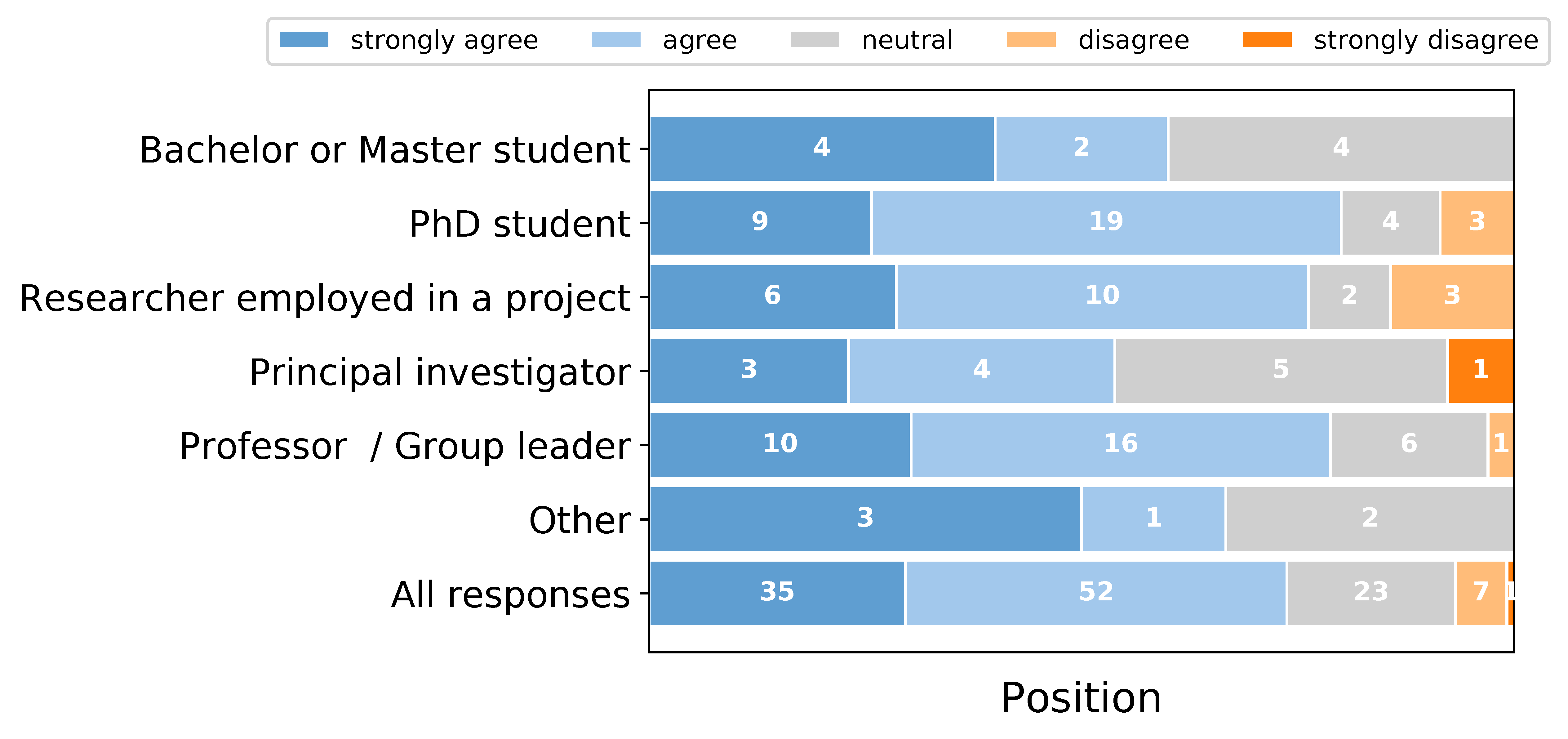

Supplement: Supplemental Information 2 — The answers to each survey question have been evaluated (1) grouped by position, (2) grouped by position, with all groups smaller than a threshold of 10 being summarized in one “other” category, (3) grouped by area of research, (4) grouped by area of research, with all groups smaller than a threshold of 10 being summarized in one “other” category, (5) grouped by research environment, (6) grouped by research environment, with all groups smaller than a threshold of 10 being summarized in one “other” category. [file peerj-cs-05-240-s002.zip › reproducibility-survey-analysis-bypositionthreshold-question-08.png]

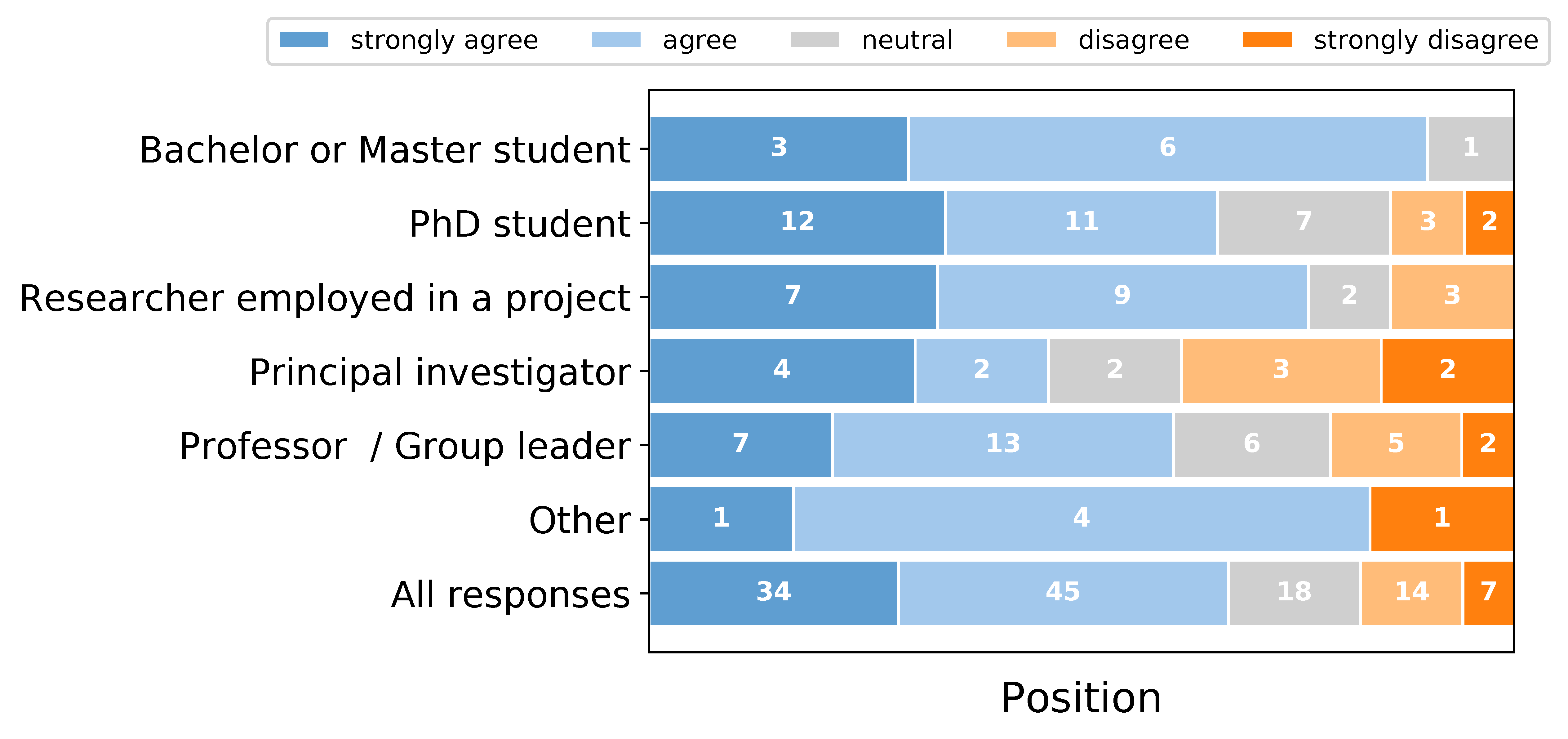

Supplement: Supplemental Information 2 — The answers to each survey question have been evaluated (1) grouped by position, (2) grouped by position, with all groups smaller than a threshold of 10 being summarized in one “other” category, (3) grouped by area of research, (4) grouped by area of research, with all groups smaller than a threshold of 10 being summarized in one “other” category, (5) grouped by research environment, (6) grouped by research environment, with all groups smaller than a threshold of 10 being summarized in one “other” category. [file peerj-cs-05-240-s002.zip › reproducibility-survey-analysis-bypositionthreshold-question-09.png]

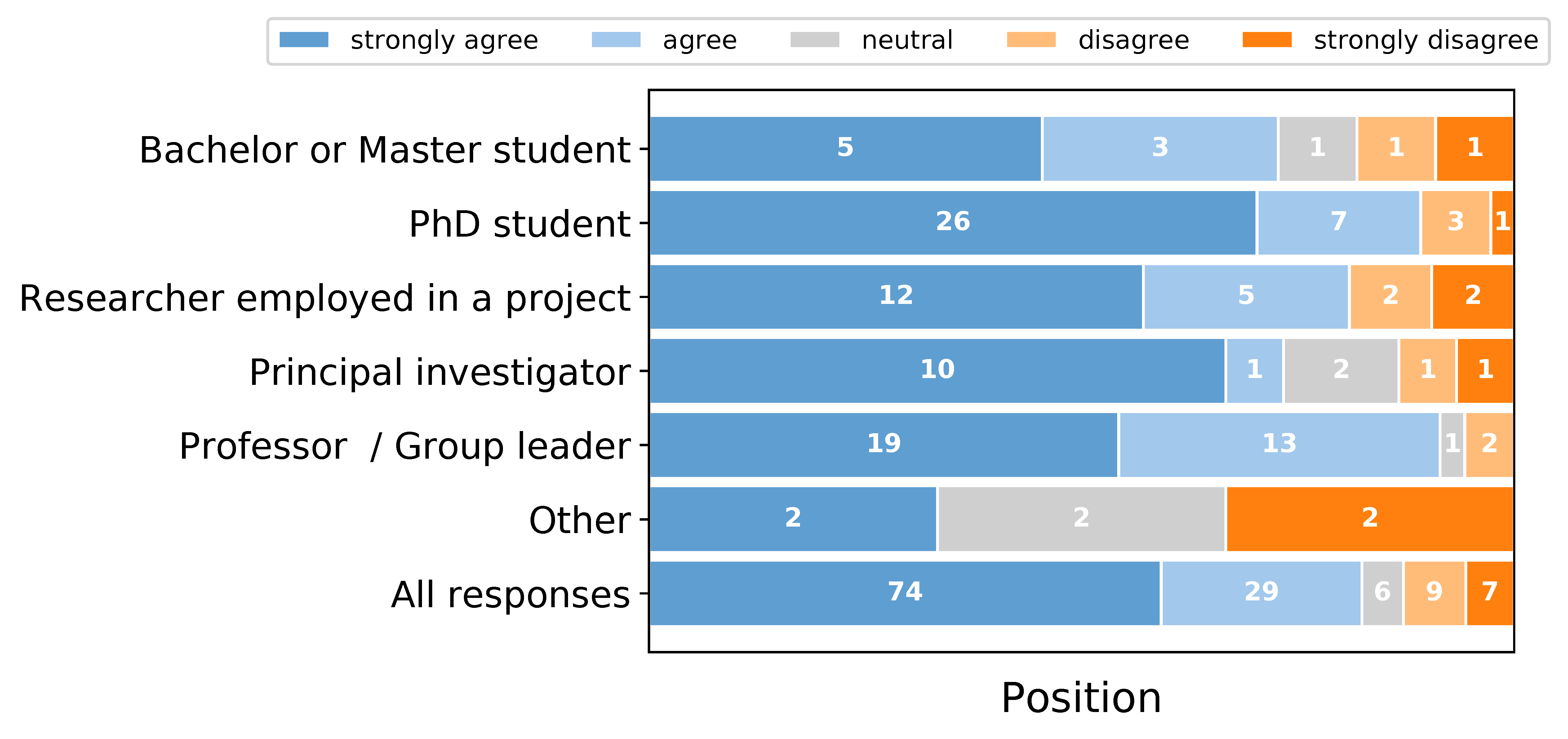

Supplement: Supplemental Information 2 — The answers to each survey question have been evaluated (1) grouped by position, (2) grouped by position, with all groups smaller than a threshold of 10 being summarized in one “other” category, (3) grouped by area of research, (4) grouped by area of research, with all groups smaller than a threshold of 10 being summarized in one “other” category, (5) grouped by research environment, (6) grouped by research environment, with all groups smaller than a threshold of 10 being summarized in one “other” category. [file peerj-cs-05-240-s002.zip › reproducibility-survey-analysis-bypositionthreshold-question-10.png]

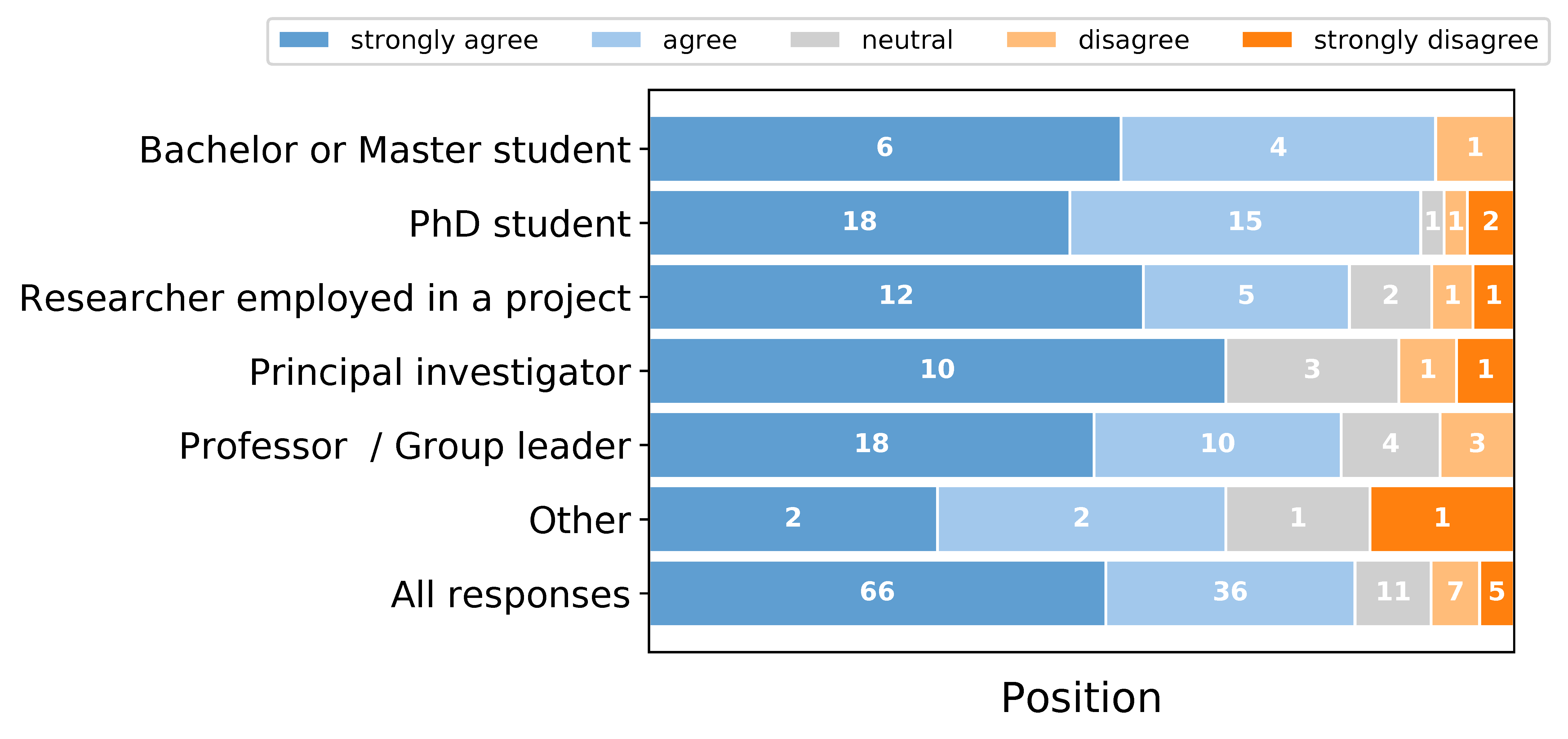

Supplement: Supplemental Information 2 — The answers to each survey question have been evaluated (1) grouped by position, (2) grouped by position, with all groups smaller than a threshold of 10 being summarized in one “other” category, (3) grouped by area of research, (4) grouped by area of research, with all groups smaller than a threshold of 10 being summarized in one “other” category, (5) grouped by research environment, (6) grouped by research environment, with all groups smaller than a threshold of 10 being summarized in one “other” category. [file peerj-cs-05-240-s002.zip › reproducibility-survey-analysis-bypositionthreshold-question-11.png]

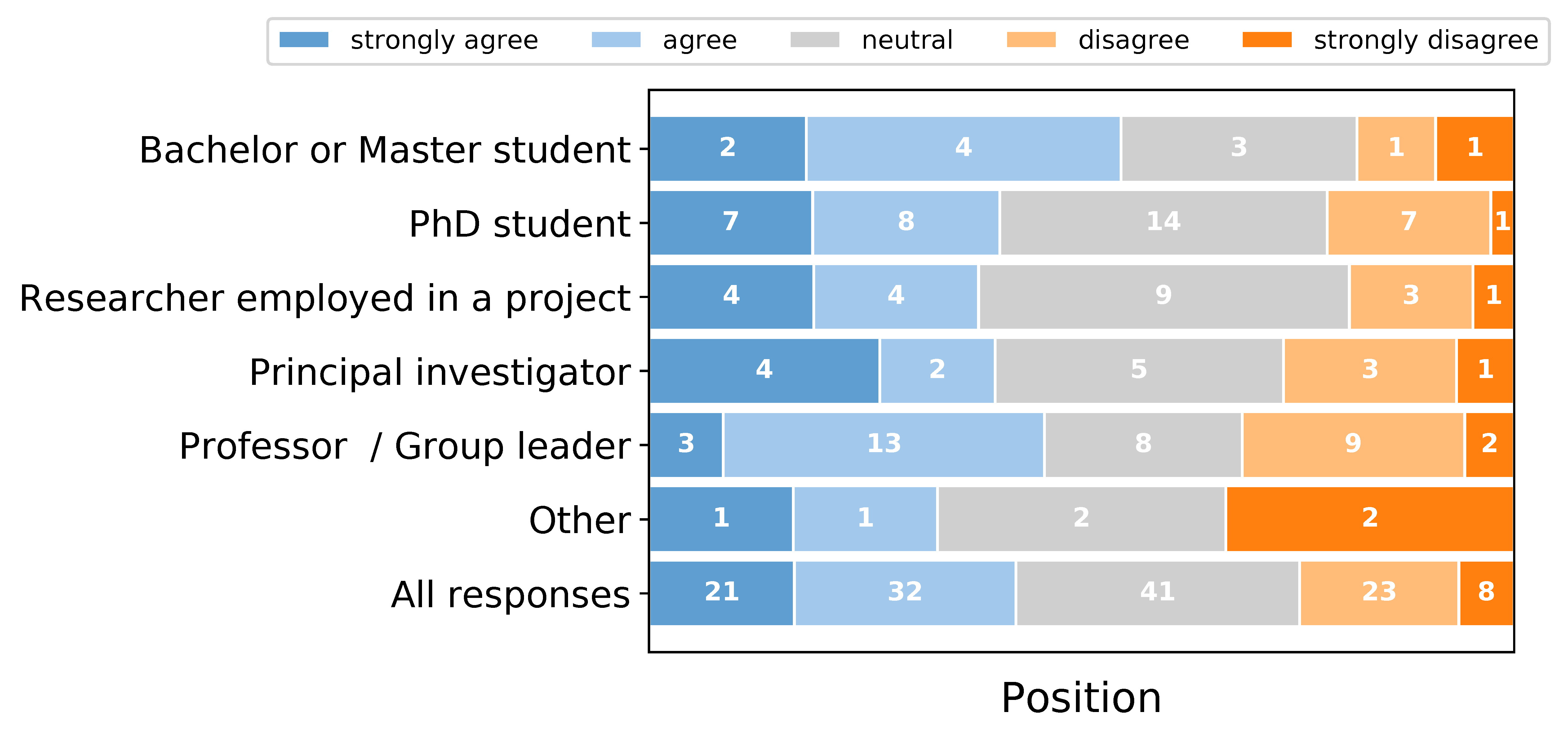

Supplement: Supplemental Information 2 — The answers to each survey question have been evaluated (1) grouped by position, (2) grouped by position, with all groups smaller than a threshold of 10 being summarized in one “other” category, (3) grouped by area of research, (4) grouped by area of research, with all groups smaller than a threshold of 10 being summarized in one “other” category, (5) grouped by research environment, (6) grouped by research environment, with all groups smaller than a threshold of 10 being summarized in one “other” category. [file peerj-cs-05-240-s002.zip › reproducibility-survey-analysis-bypositionthreshold-question-12.png]

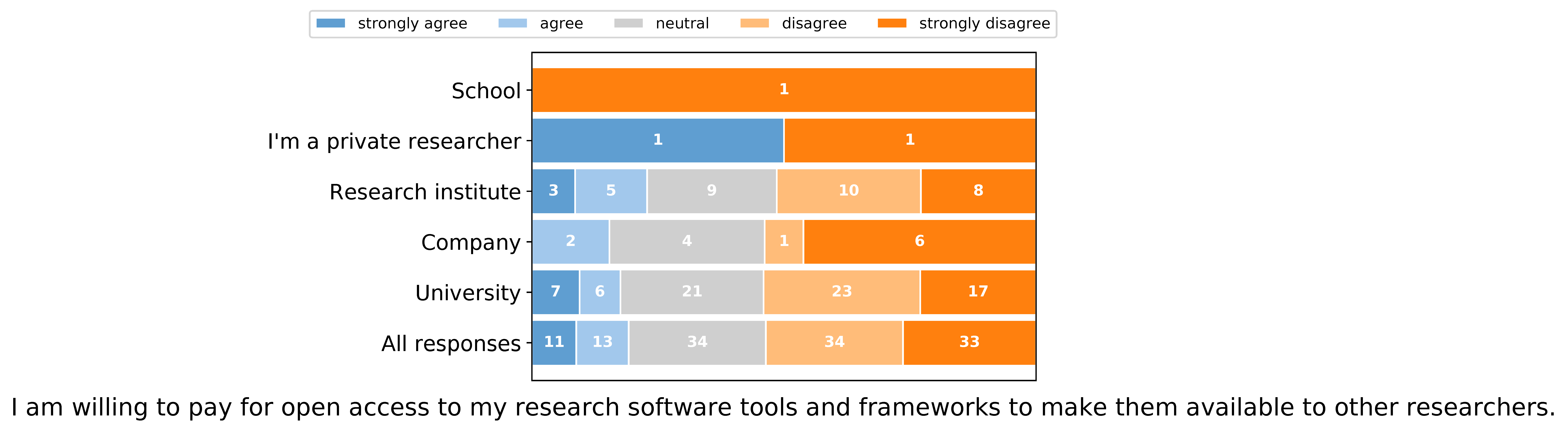

Supplement: Supplemental Information 2 — The answers to each survey question have been evaluated (1) grouped by position, (2) grouped by position, with all groups smaller than a threshold of 10 being summarized in one “other” category, (3) grouped by area of research, (4) grouped by area of research, with all groups smaller than a threshold of 10 being summarized in one “other” category, (5) grouped by research environment, (6) grouped by research environment, with all groups smaller than a threshold of 10 being summarized in one “other” category. [file peerj-cs-05-240-s002.zip › reproducibility-survey-analysis-byresearchenvironment-question-01.png]

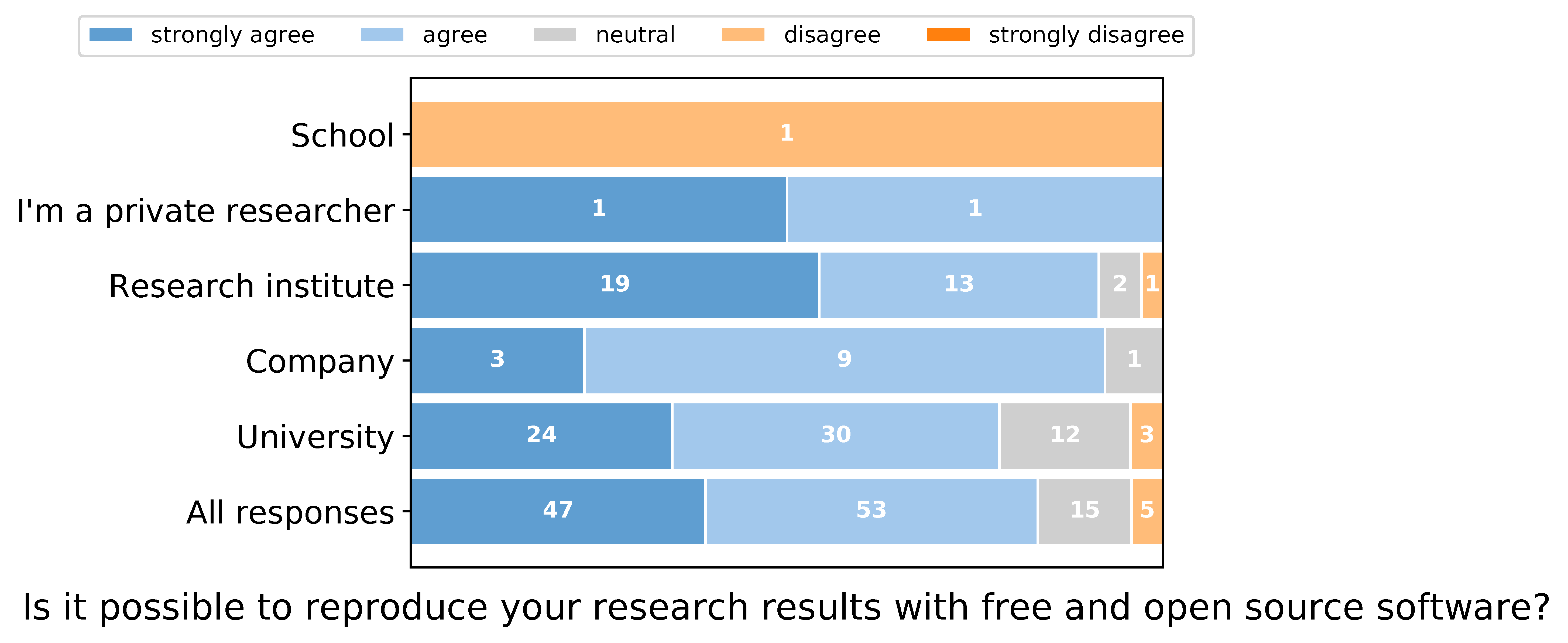

Supplement: Supplemental Information 2 — The answers to each survey question have been evaluated (1) grouped by position, (2) grouped by position, with all groups smaller than a threshold of 10 being summarized in one “other” category, (3) grouped by area of research, (4) grouped by area of research, with all groups smaller than a threshold of 10 being summarized in one “other” category, (5) grouped by research environment, (6) grouped by research environment, with all groups smaller than a threshold of 10 being summarized in one “other” category. [file peerj-cs-05-240-s002.zip › reproducibility-survey-analysis-byresearchenvironment-question-02.png]

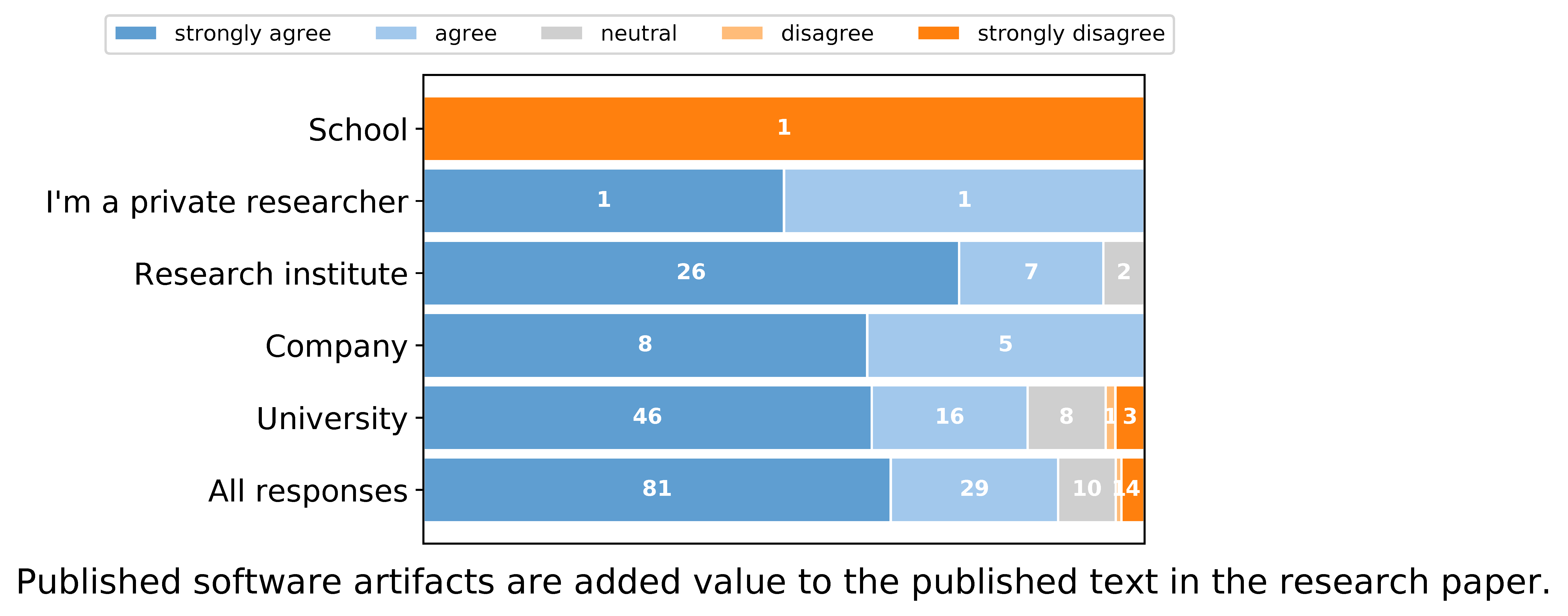

Supplement: Supplemental Information 2 — The answers to each survey question have been evaluated (1) grouped by position, (2) grouped by position, with all groups smaller than a threshold of 10 being summarized in one “other” category, (3) grouped by area of research, (4) grouped by area of research, with all groups smaller than a threshold of 10 being summarized in one “other” category, (5) grouped by research environment, (6) grouped by research environment, with all groups smaller than a threshold of 10 being summarized in one “other” category. [file peerj-cs-05-240-s002.zip › reproducibility-survey-analysis-byresearchenvironment-question-03.png]

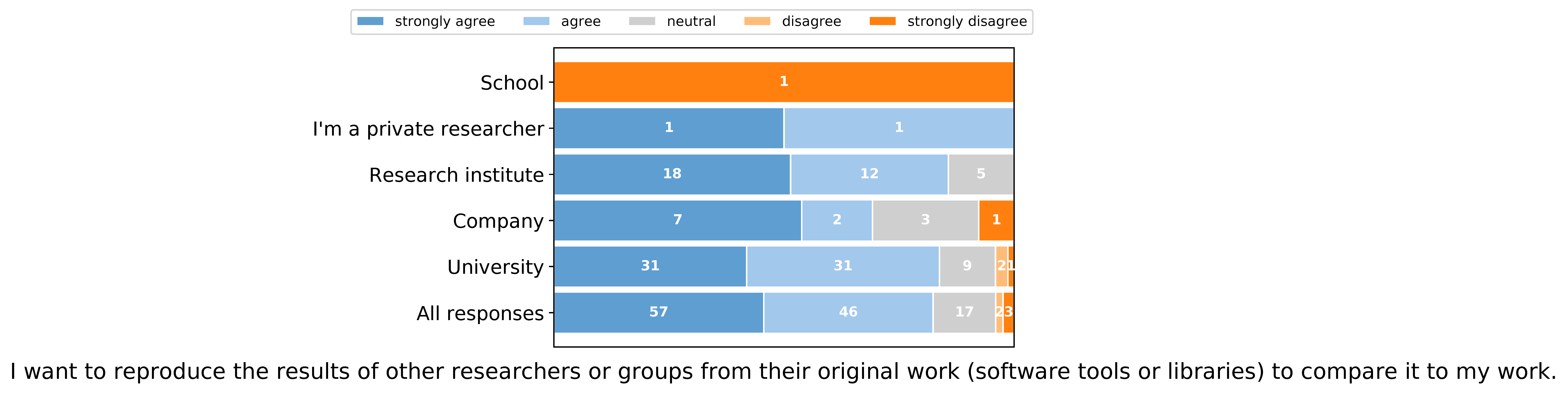

Supplement: Supplemental Information 2 — The answers to each survey question have been evaluated (1) grouped by position, (2) grouped by position, with all groups smaller than a threshold of 10 being summarized in one “other” category, (3) grouped by area of research, (4) grouped by area of research, with all groups smaller than a threshold of 10 being summarized in one “other” category, (5) grouped by research environment, (6) grouped by research environment, with all groups smaller than a threshold of 10 being summarized in one “other” category. [file peerj-cs-05-240-s002.zip › reproducibility-survey-analysis-byresearchenvironment-question-04.png]

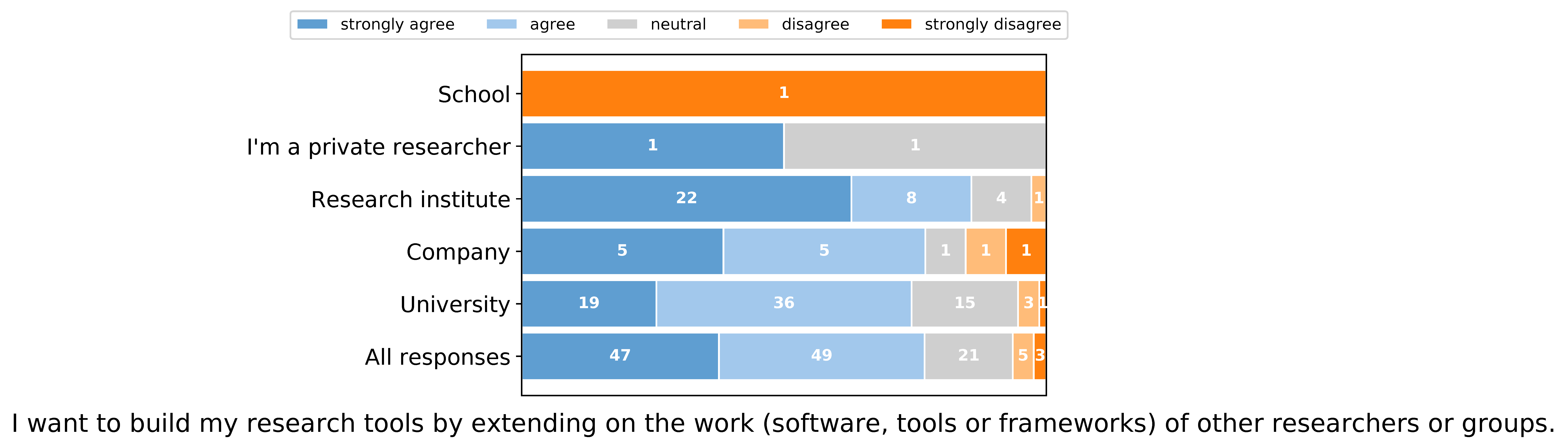

Supplement: Supplemental Information 2 — The answers to each survey question have been evaluated (1) grouped by position, (2) grouped by position, with all groups smaller than a threshold of 10 being summarized in one “other” category, (3) grouped by area of research, (4) grouped by area of research, with all groups smaller than a threshold of 10 being summarized in one “other” category, (5) grouped by research environment, (6) grouped by research environment, with all groups smaller than a threshold of 10 being summarized in one “other” category. [file peerj-cs-05-240-s002.zip › reproducibility-survey-analysis-byresearchenvironment-question-05.png]

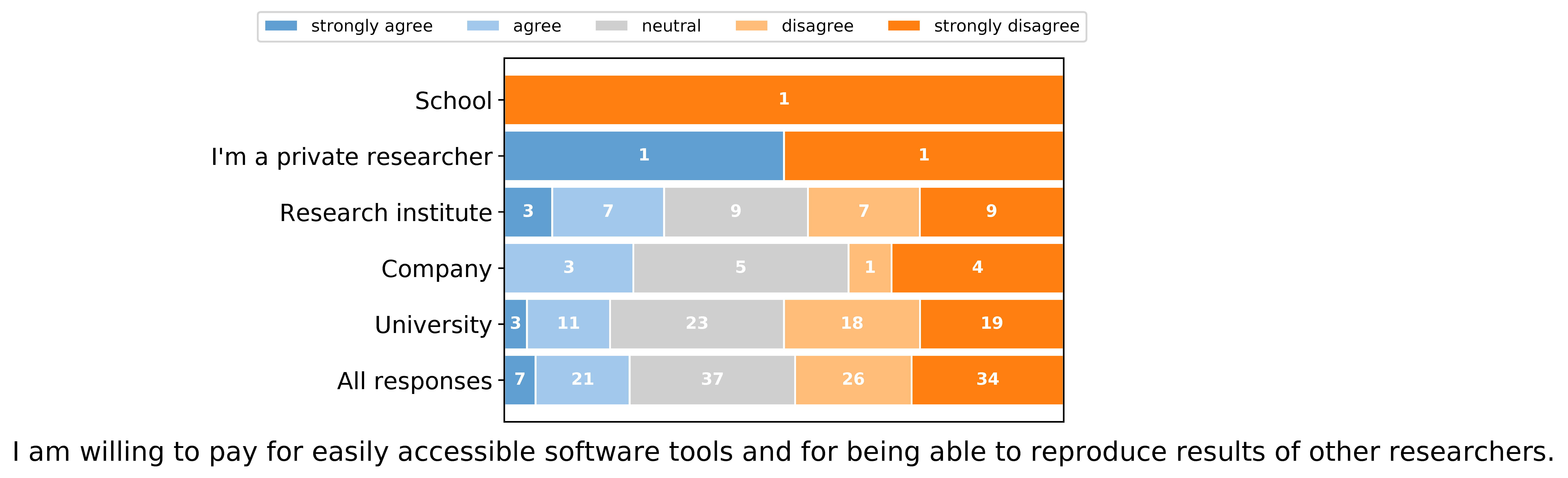

Supplement: Supplemental Information 2 — The answers to each survey question have been evaluated (1) grouped by position, (2) grouped by position, with all groups smaller than a threshold of 10 being summarized in one “other” category, (3) grouped by area of research, (4) grouped by area of research, with all groups smaller than a threshold of 10 being summarized in one “other” category, (5) grouped by research environment, (6) grouped by research environment, with all groups smaller than a threshold of 10 being summarized in one “other” category. [file peerj-cs-05-240-s002.zip › reproducibility-survey-analysis-byresearchenvironment-question-06.png]

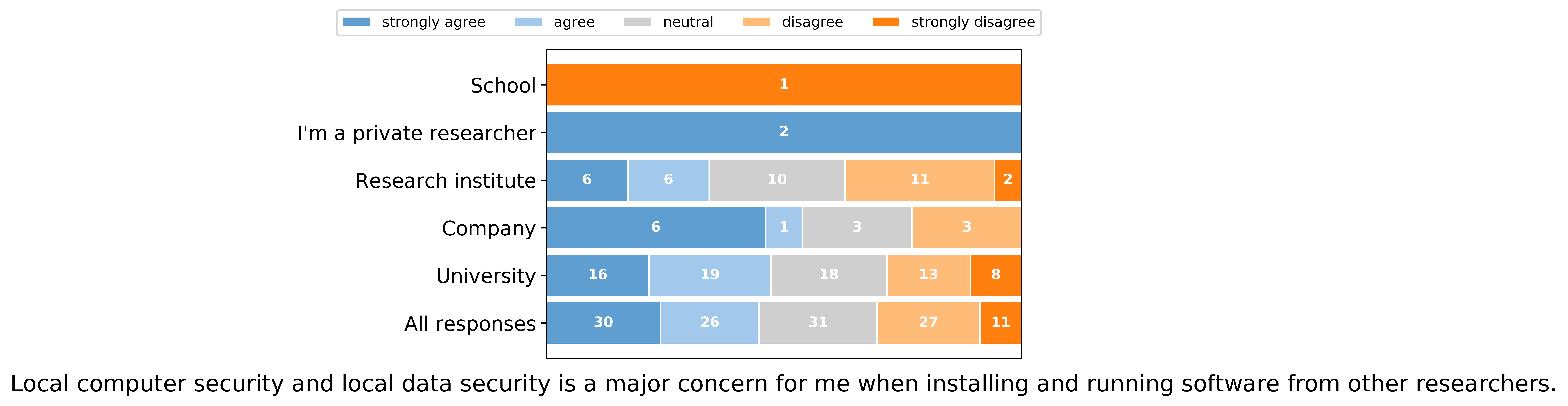

Supplement: Supplemental Information 2 — The answers to each survey question have been evaluated (1) grouped by position, (2) grouped by position, with all groups smaller than a threshold of 10 being summarized in one “other” category, (3) grouped by area of research, (4) grouped by area of research, with all groups smaller than a threshold of 10 being summarized in one “other” category, (5) grouped by research environment, (6) grouped by research environment, with all groups smaller than a threshold of 10 being summarized in one “other” category. [file peerj-cs-05-240-s002.zip › reproducibility-survey-analysis-byresearchenvironment-question-07.png]

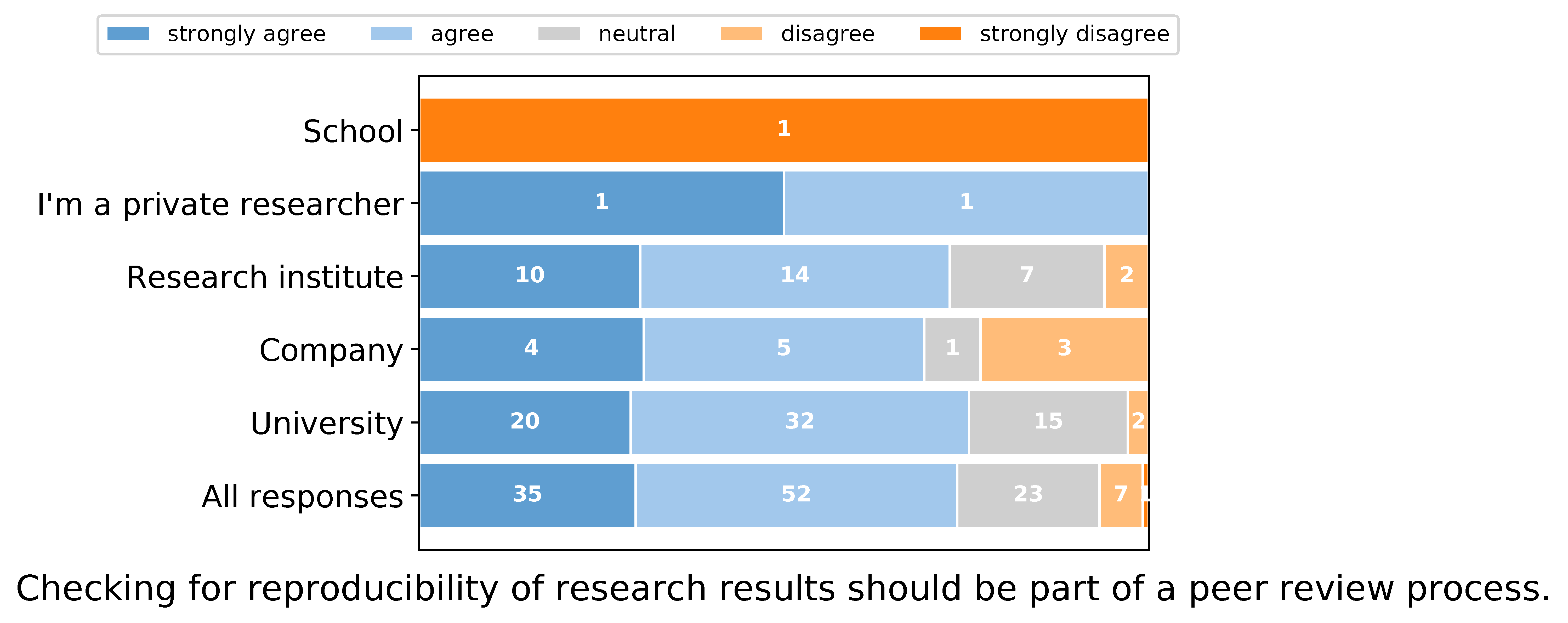

Supplement: Supplemental Information 2 — The answers to each survey question have been evaluated (1) grouped by position, (2) grouped by position, with all groups smaller than a threshold of 10 being summarized in one “other” category, (3) grouped by area of research, (4) grouped by area of research, with all groups smaller than a threshold of 10 being summarized in one “other” category, (5) grouped by research environment, (6) grouped by research environment, with all groups smaller than a threshold of 10 being summarized in one “other” category. [file peerj-cs-05-240-s002.zip › reproducibility-survey-analysis-byresearchenvironment-question-08.png]

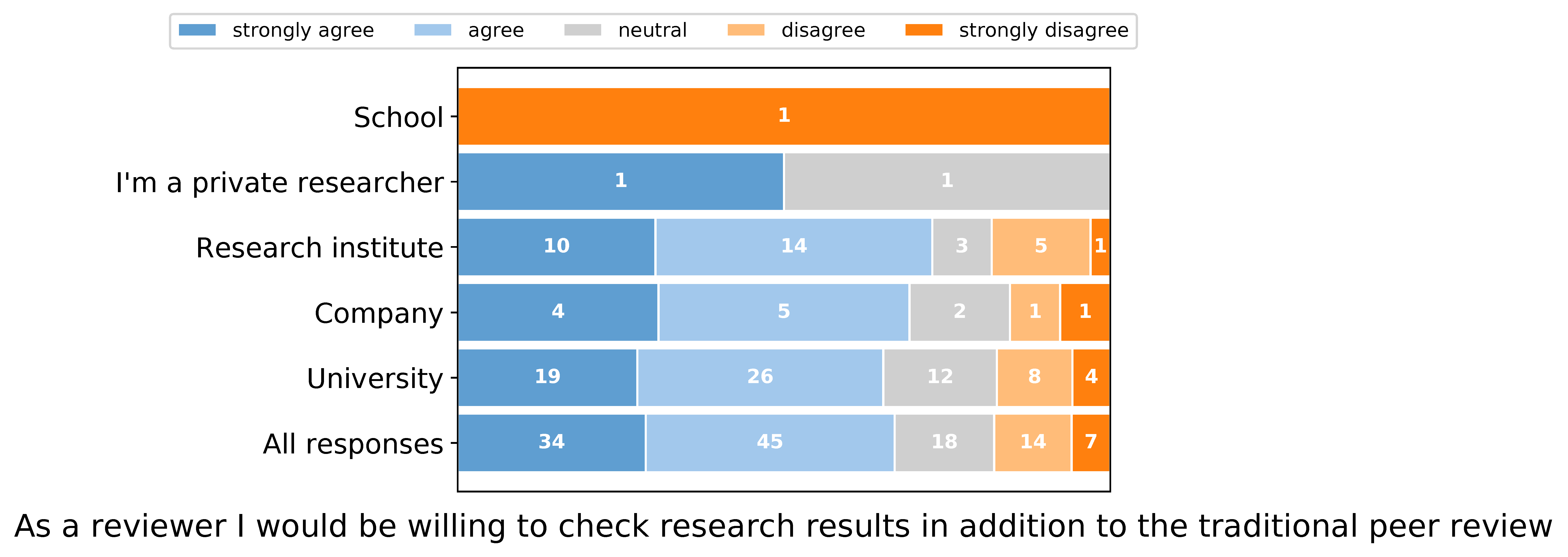

Supplement: Supplemental Information 2 — The answers to each survey question have been evaluated (1) grouped by position, (2) grouped by position, with all groups smaller than a threshold of 10 being summarized in one “other” category, (3) grouped by area of research, (4) grouped by area of research, with all groups smaller than a threshold of 10 being summarized in one “other” category, (5) grouped by research environment, (6) grouped by research environment, with all groups smaller than a threshold of 10 being summarized in one “other” category. [file peerj-cs-05-240-s002.zip › reproducibility-survey-analysis-byresearchenvironment-question-09.png]

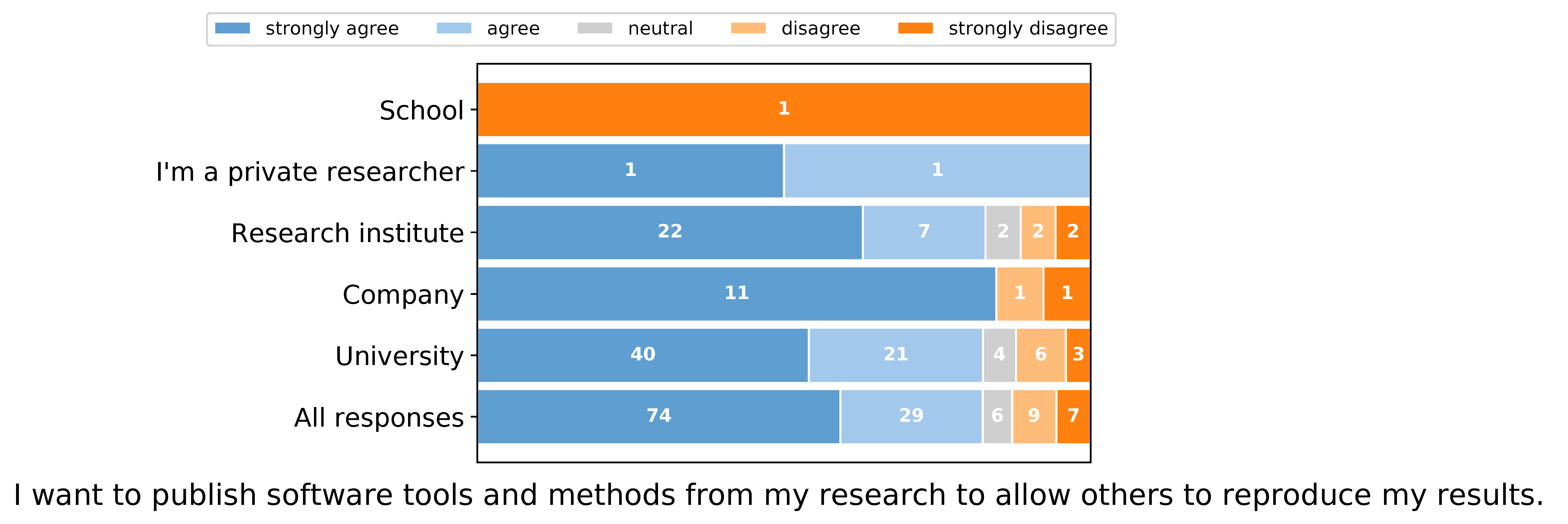

Supplement: Supplemental Information 2 — The answers to each survey question have been evaluated (1) grouped by position, (2) grouped by position, with all groups smaller than a threshold of 10 being summarized in one “other” category, (3) grouped by area of research, (4) grouped by area of research, with all groups smaller than a threshold of 10 being summarized in one “other” category, (5) grouped by research environment, (6) grouped by research environment, with all groups smaller than a threshold of 10 being summarized in one “other” category. [file peerj-cs-05-240-s002.zip › reproducibility-survey-analysis-byresearchenvironment-question-10.png]

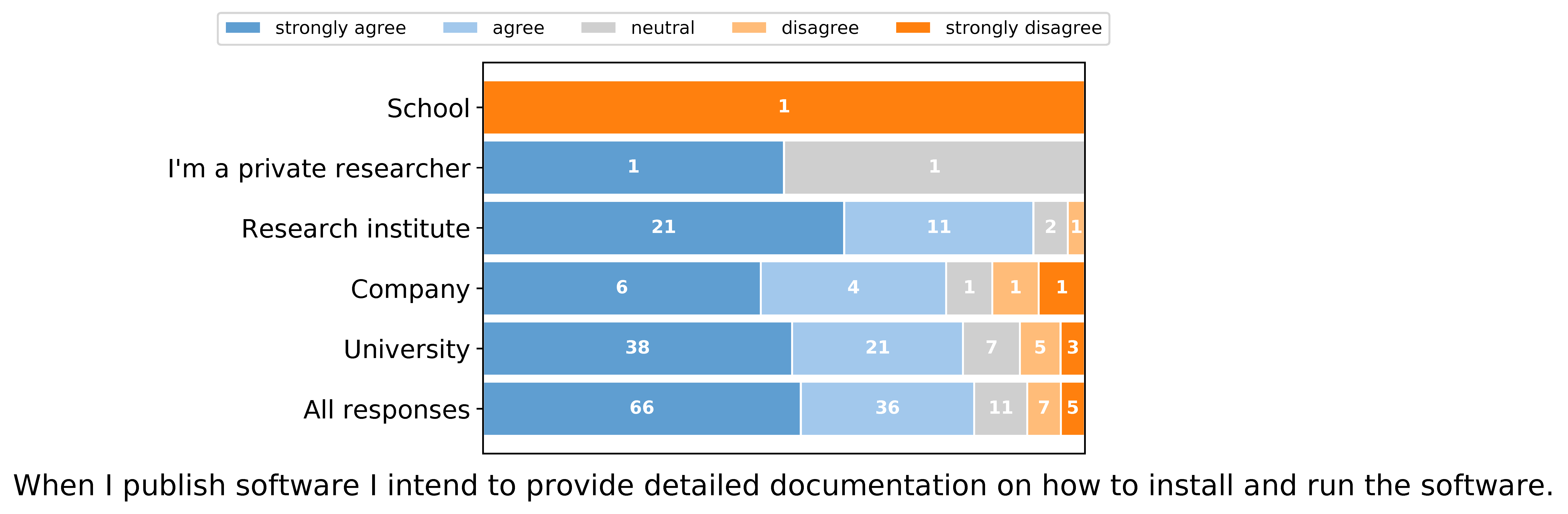

Supplement: Supplemental Information 2 — The answers to each survey question have been evaluated (1) grouped by position, (2) grouped by position, with all groups smaller than a threshold of 10 being summarized in one “other” category, (3) grouped by area of research, (4) grouped by area of research, with all groups smaller than a threshold of 10 being summarized in one “other” category, (5) grouped by research environment, (6) grouped by research environment, with all groups smaller than a threshold of 10 being summarized in one “other” category. [file peerj-cs-05-240-s002.zip › reproducibility-survey-analysis-byresearchenvironment-question-11.png]

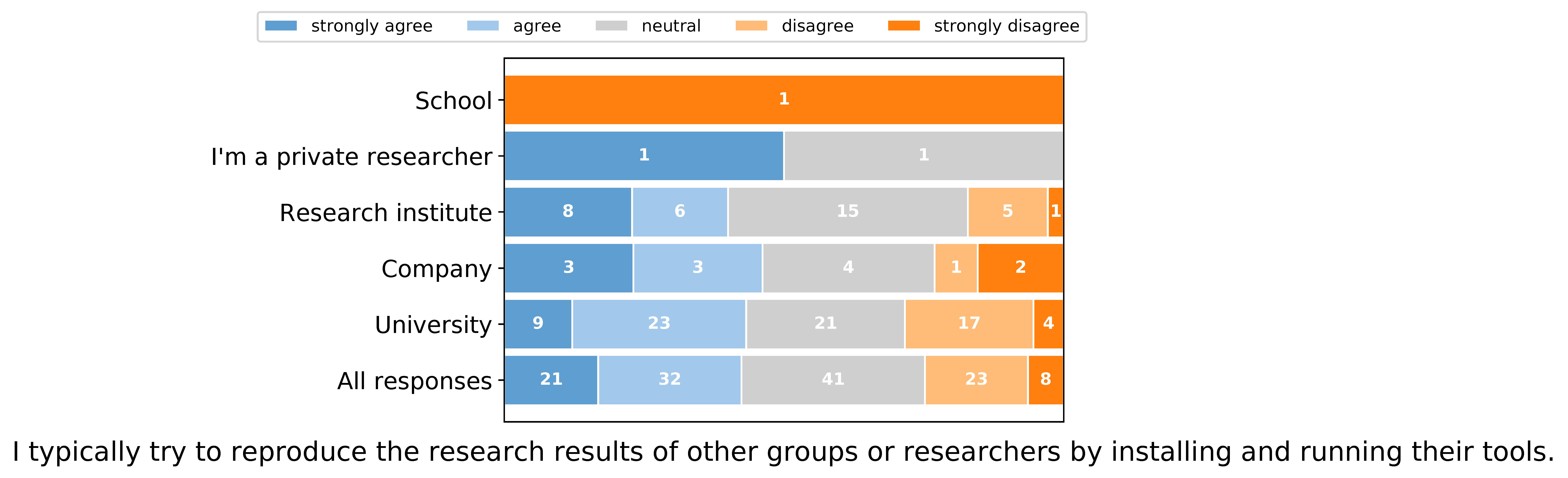

Supplement: Supplemental Information 2 — The answers to each survey question have been evaluated (1) grouped by position, (2) grouped by position, with all groups smaller than a threshold of 10 being summarized in one “other” category, (3) grouped by area of research, (4) grouped by area of research, with all groups smaller than a threshold of 10 being summarized in one “other” category, (5) grouped by research environment, (6) grouped by research environment, with all groups smaller than a threshold of 10 being summarized in one “other” category. [file peerj-cs-05-240-s002.zip › reproducibility-survey-analysis-byresearchenvironment-question-12.png]

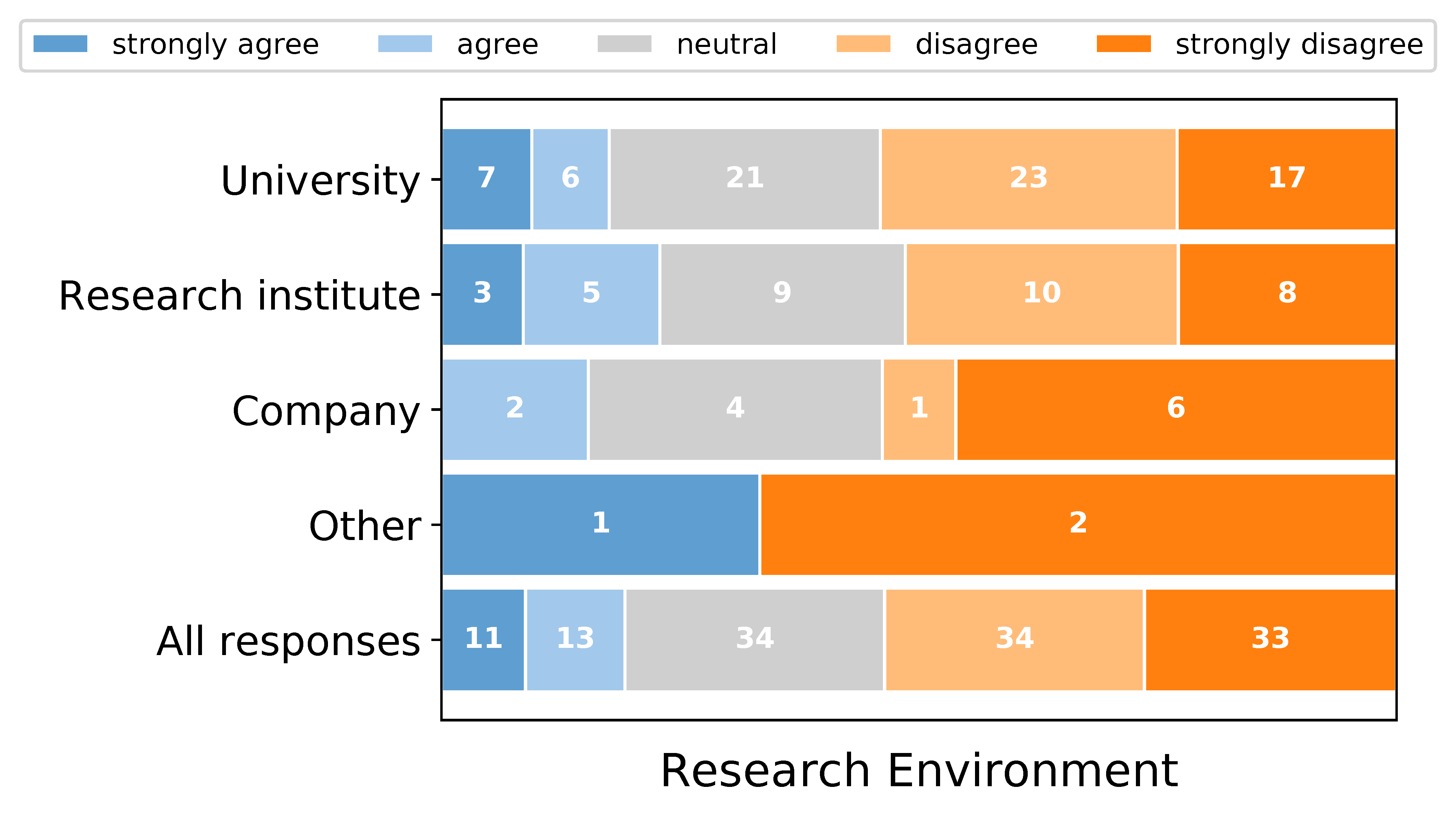

Supplement: Supplemental Information 2 — The answers to each survey question have been evaluated (1) grouped by position, (2) grouped by position, with all groups smaller than a threshold of 10 being summarized in one “other” category, (3) grouped by area of research, (4) grouped by area of research, with all groups smaller than a threshold of 10 being summarized in one “other” category, (5) grouped by research environment, (6) grouped by research environment, with all groups smaller than a threshold of 10 being summarized in one “other” category. [file peerj-cs-05-240-s002.zip › reproducibility-survey-analysis-byresearchenvironmentthreshold-question-01.png]

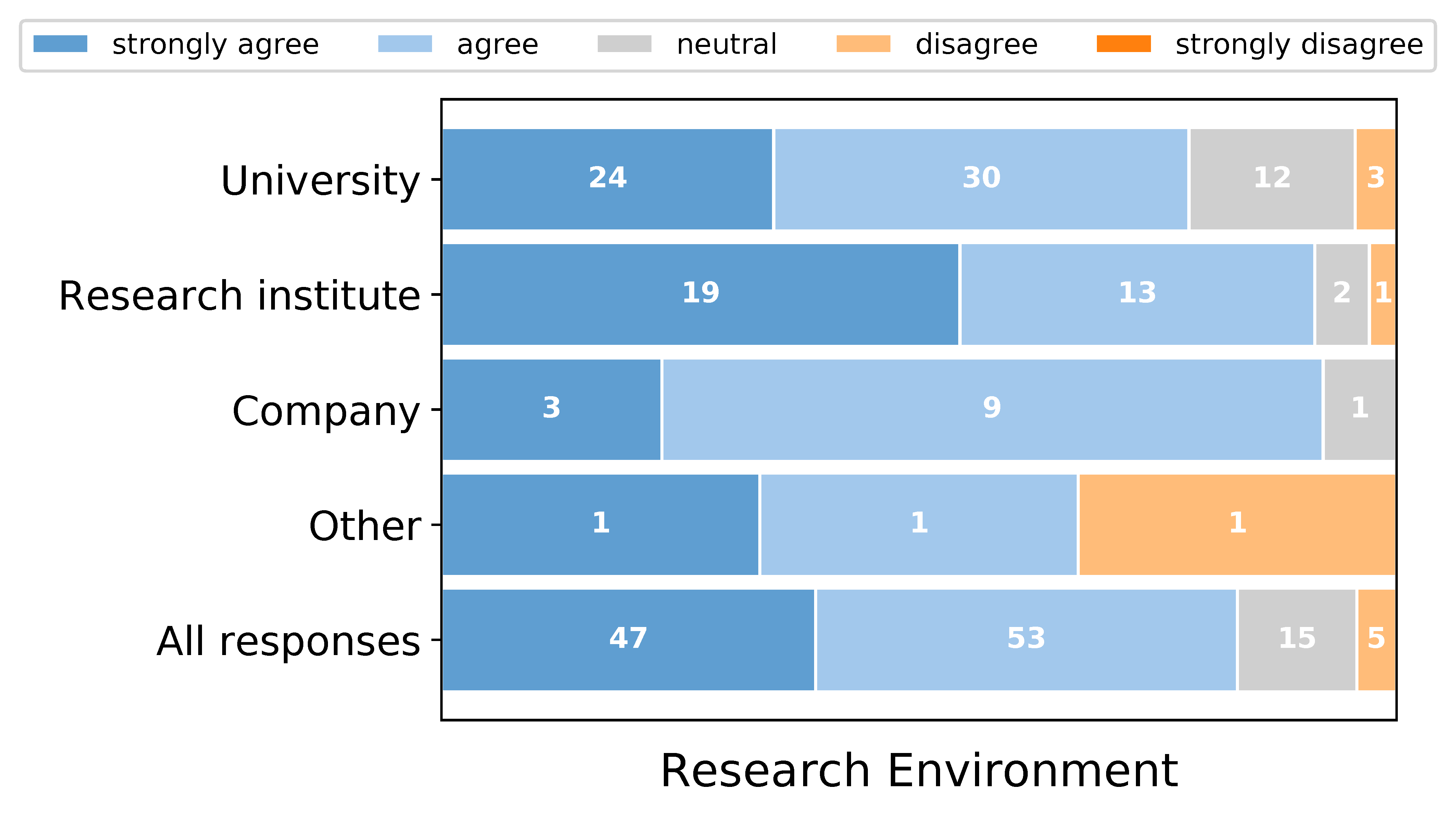

Supplement: Supplemental Information 2 — The answers to each survey question have been evaluated (1) grouped by position, (2) grouped by position, with all groups smaller than a threshold of 10 being summarized in one “other” category, (3) grouped by area of research, (4) grouped by area of research, with all groups smaller than a threshold of 10 being summarized in one “other” category, (5) grouped by research environment, (6) grouped by research environment, with all groups smaller than a threshold of 10 being summarized in one “other” category. [file peerj-cs-05-240-s002.zip › reproducibility-survey-analysis-byresearchenvironmentthreshold-question-02.png]

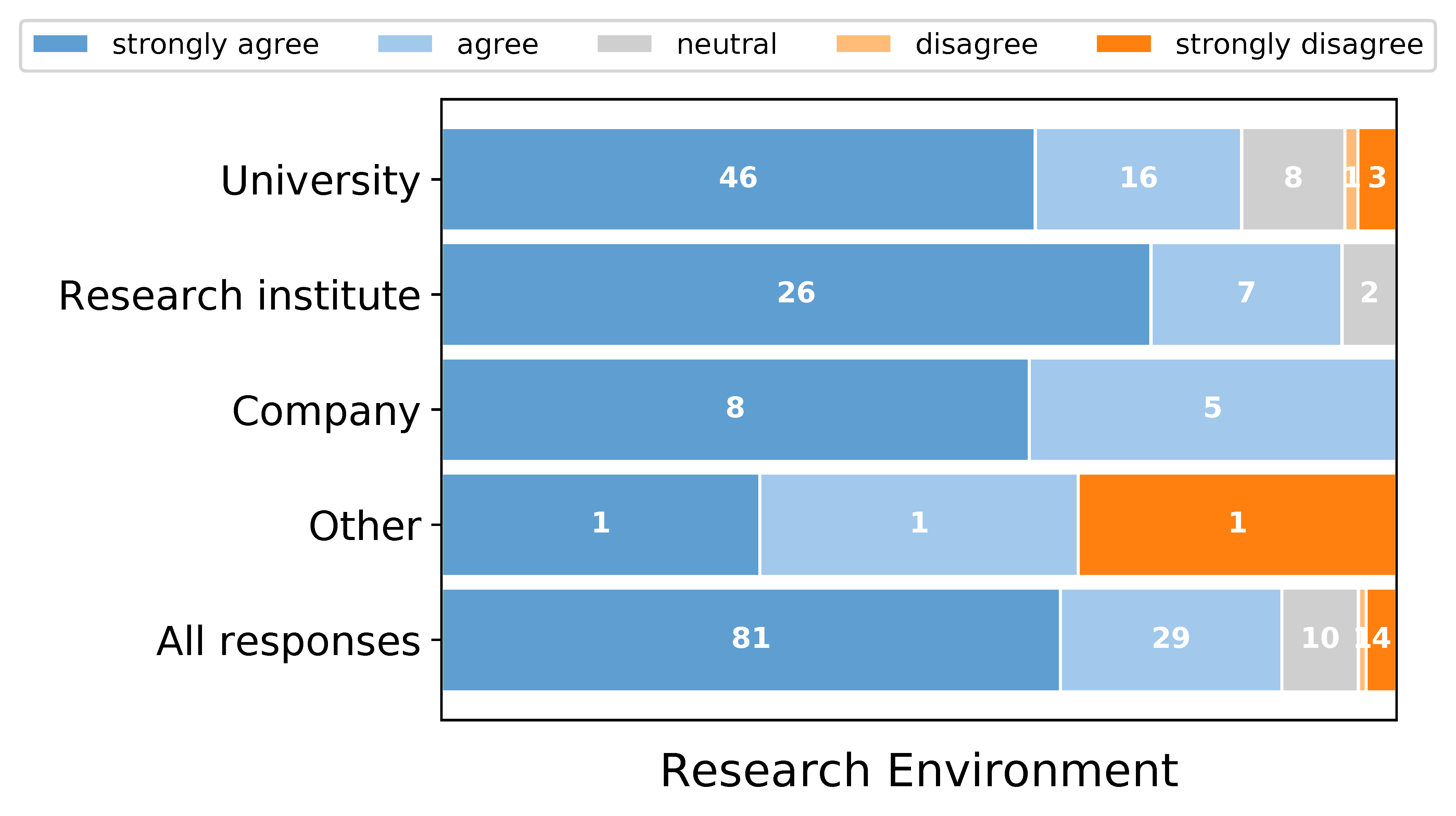

Supplement: Supplemental Information 2 — The answers to each survey question have been evaluated (1) grouped by position, (2) grouped by position, with all groups smaller than a threshold of 10 being summarized in one “other” category, (3) grouped by area of research, (4) grouped by area of research, with all groups smaller than a threshold of 10 being summarized in one “other” category, (5) grouped by research environment, (6) grouped by research environment, with all groups smaller than a threshold of 10 being summarized in one “other” category. [file peerj-cs-05-240-s002.zip › reproducibility-survey-analysis-byresearchenvironmentthreshold-question-03.png]

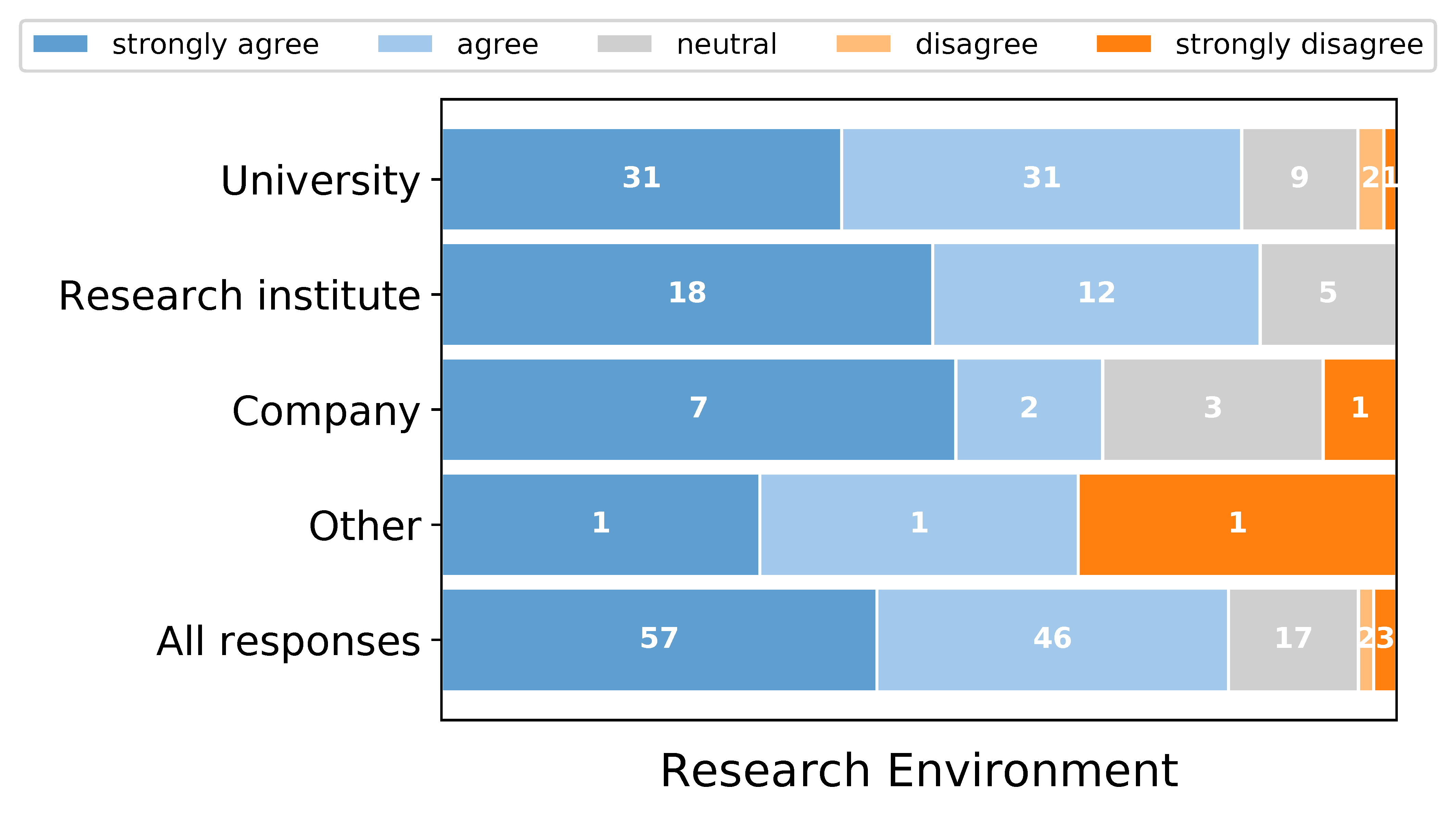

Supplement: Supplemental Information 2 — The answers to each survey question have been evaluated (1) grouped by position, (2) grouped by position, with all groups smaller than a threshold of 10 being summarized in one “other” category, (3) grouped by area of research, (4) grouped by area of research, with all groups smaller than a threshold of 10 being summarized in one “other” category, (5) grouped by research environment, (6) grouped by research environment, with all groups smaller than a threshold of 10 being summarized in one “other” category. [file peerj-cs-05-240-s002.zip › reproducibility-survey-analysis-byresearchenvironmentthreshold-question-04.png]

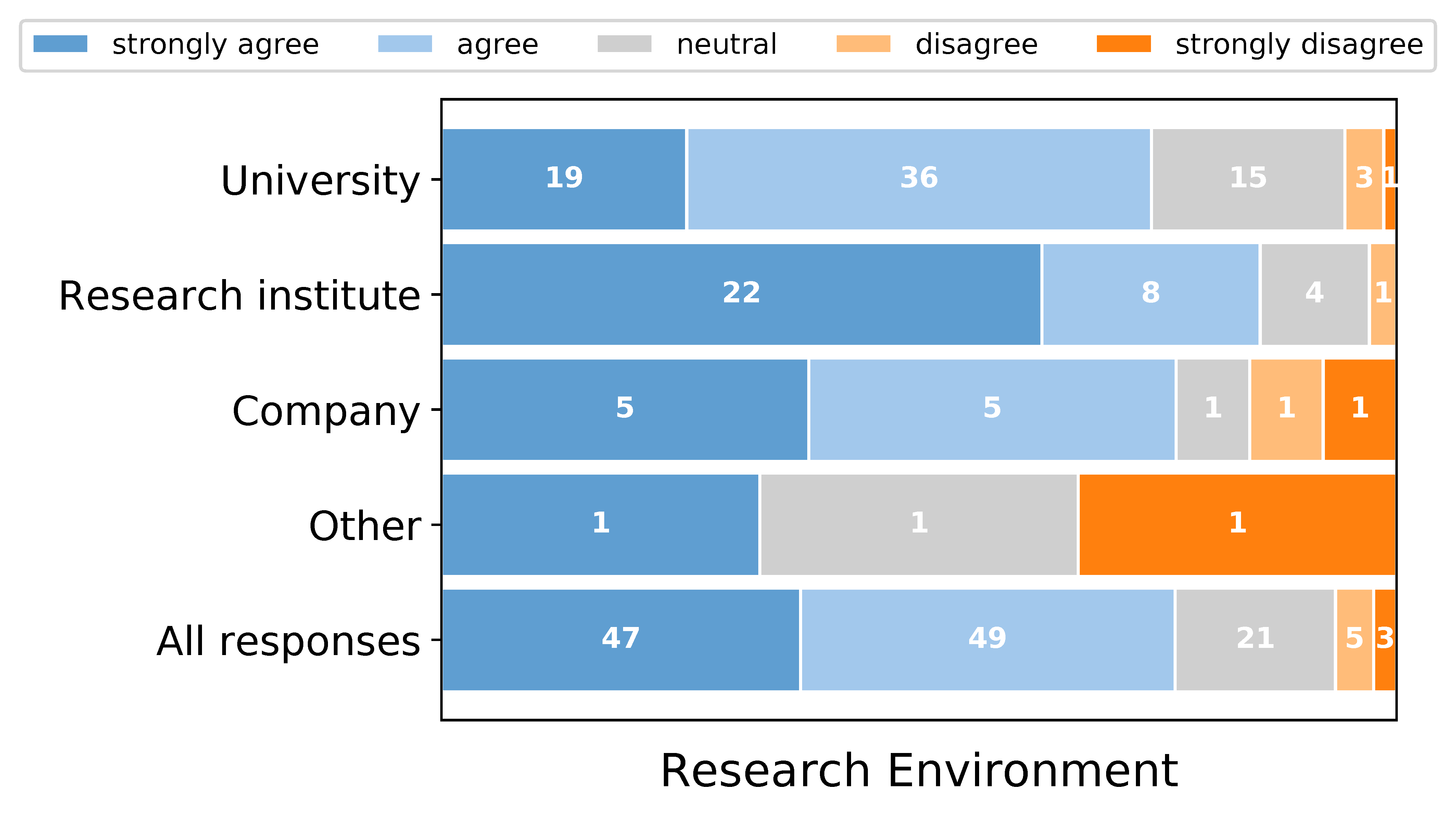

Supplement: Supplemental Information 2 — The answers to each survey question have been evaluated (1) grouped by position, (2) grouped by position, with all groups smaller than a threshold of 10 being summarized in one “other” category, (3) grouped by area of research, (4) grouped by area of research, with all groups smaller than a threshold of 10 being summarized in one “other” category, (5) grouped by research environment, (6) grouped by research environment, with all groups smaller than a threshold of 10 being summarized in one “other” category. [file peerj-cs-05-240-s002.zip › reproducibility-survey-analysis-byresearchenvironmentthreshold-question-05.png]

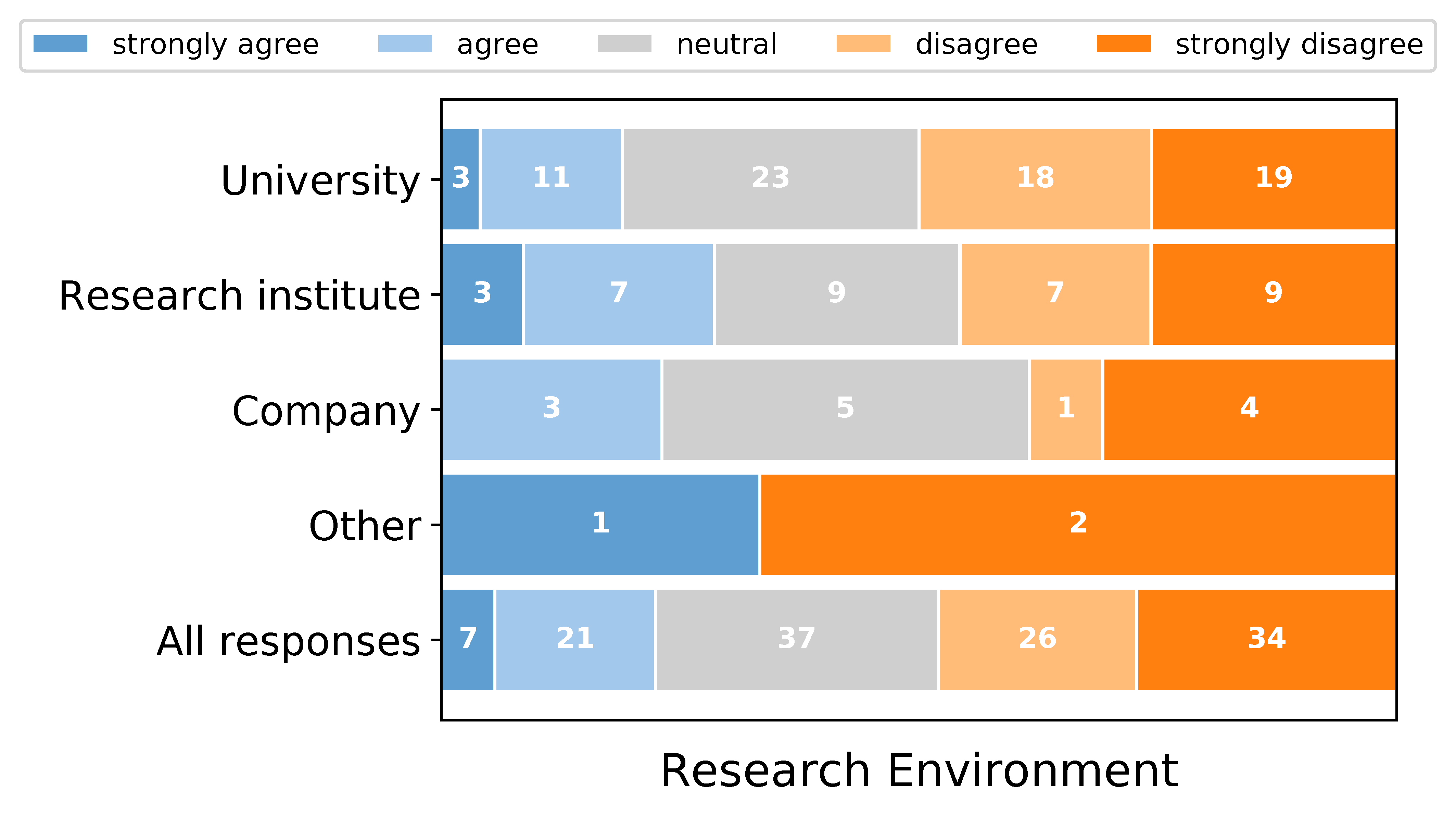

Supplement: Supplemental Information 2 — The answers to each survey question have been evaluated (1) grouped by position, (2) grouped by position, with all groups smaller than a threshold of 10 being summarized in one “other” category, (3) grouped by area of research, (4) grouped by area of research, with all groups smaller than a threshold of 10 being summarized in one “other” category, (5) grouped by research environment, (6) grouped by research environment, with all groups smaller than a threshold of 10 being summarized in one “other” category. [file peerj-cs-05-240-s002.zip › reproducibility-survey-analysis-byresearchenvironmentthreshold-question-06.png]

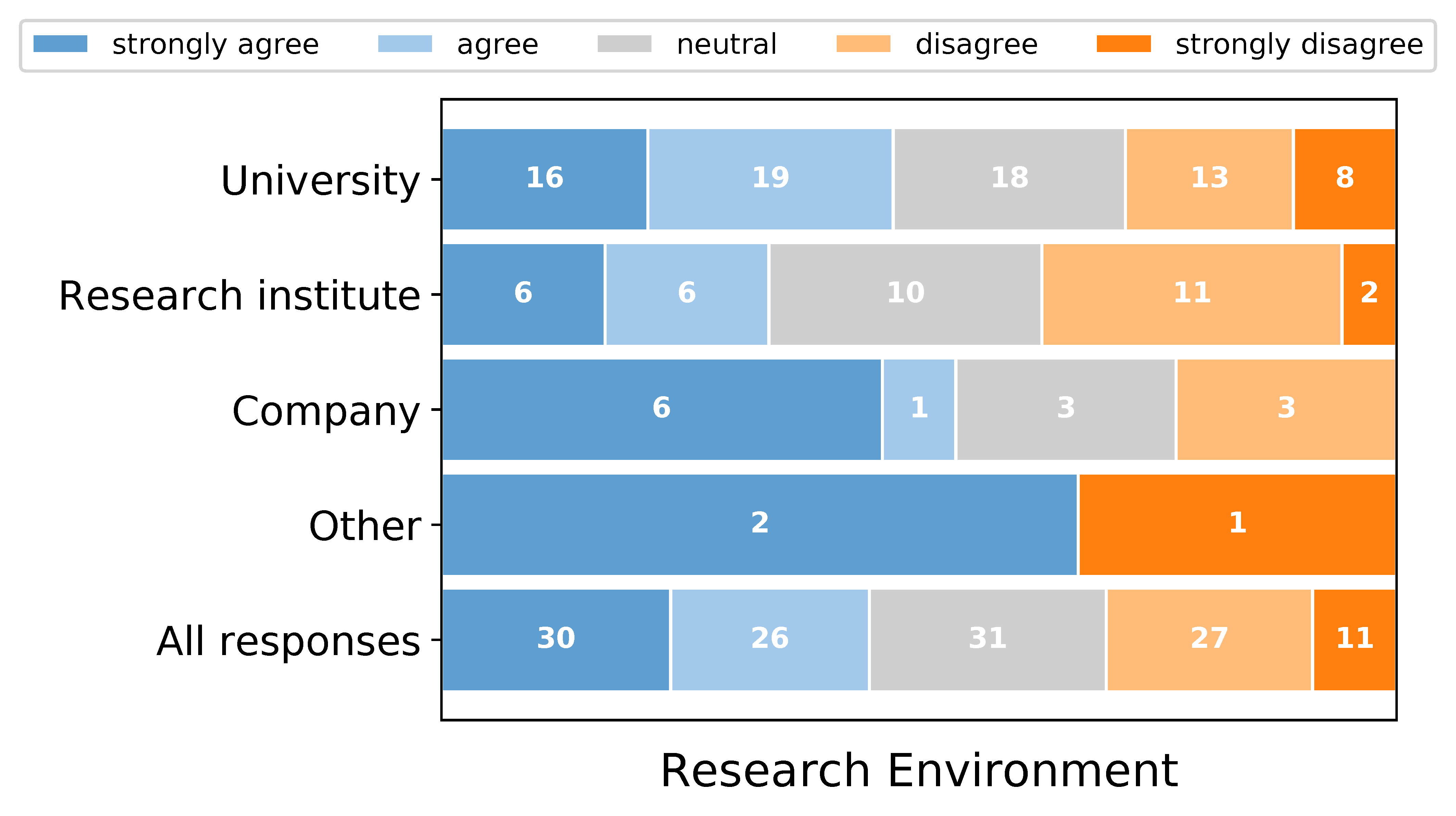

Supplement: Supplemental Information 2 — The answers to each survey question have been evaluated (1) grouped by position, (2) grouped by position, with all groups smaller than a threshold of 10 being summarized in one “other” category, (3) grouped by area of research, (4) grouped by area of research, with all groups smaller than a threshold of 10 being summarized in one “other” category, (5) grouped by research environment, (6) grouped by research environment, with all groups smaller than a threshold of 10 being summarized in one “other” category. [file peerj-cs-05-240-s002.zip › reproducibility-survey-analysis-byresearchenvironmentthreshold-question-07.png]

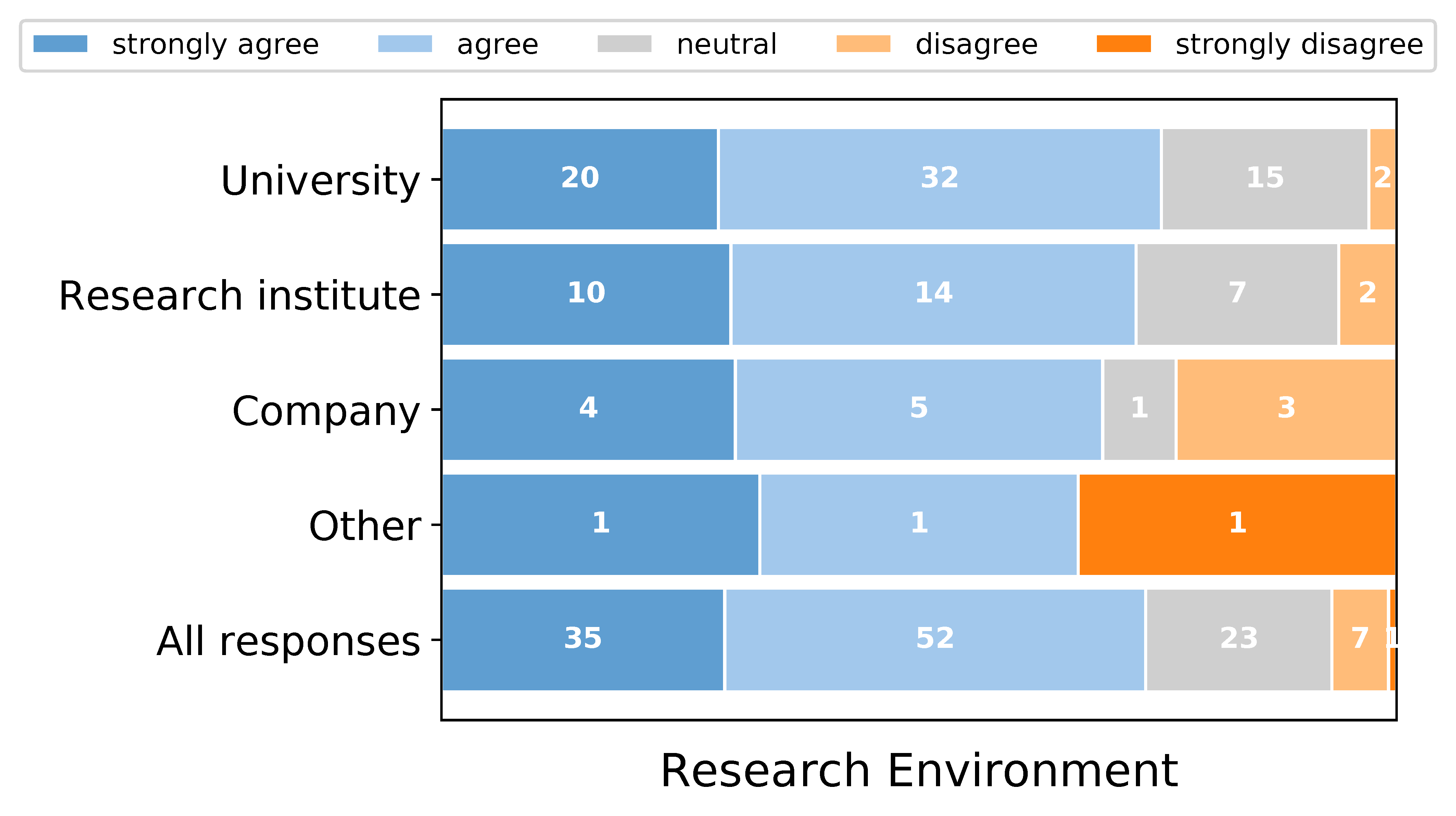

Supplement: Supplemental Information 2 — The answers to each survey question have been evaluated (1) grouped by position, (2) grouped by position, with all groups smaller than a threshold of 10 being summarized in one “other” category, (3) grouped by area of research, (4) grouped by area of research, with all groups smaller than a threshold of 10 being summarized in one “other” category, (5) grouped by research environment, (6) grouped by research environment, with all groups smaller than a threshold of 10 being summarized in one “other” category. [file peerj-cs-05-240-s002.zip › reproducibility-survey-analysis-byresearchenvironmentthreshold-question-08.png]

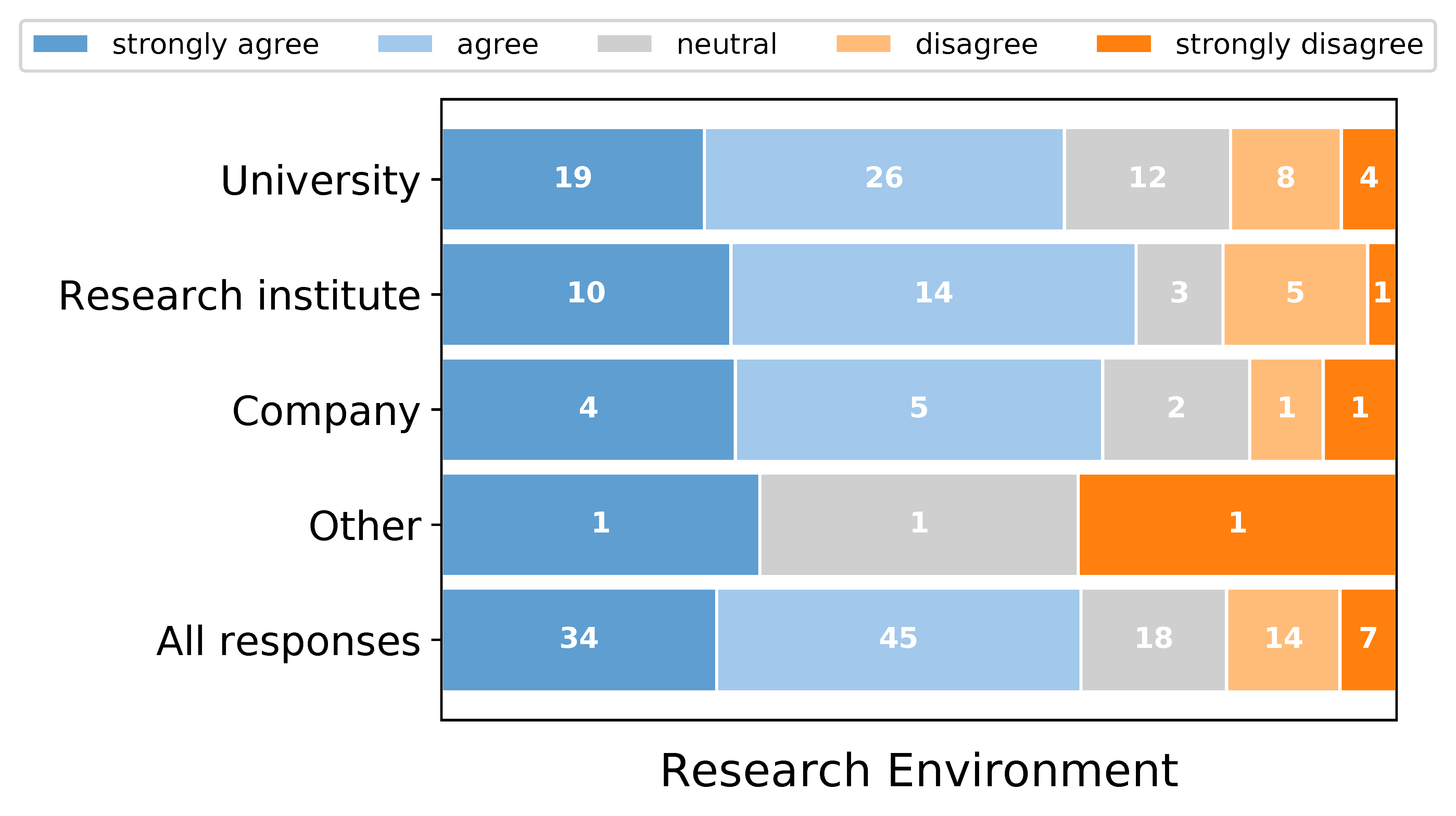

Supplement: Supplemental Information 2 — The answers to each survey question have been evaluated (1) grouped by position, (2) grouped by position, with all groups smaller than a threshold of 10 being summarized in one “other” category, (3) grouped by area of research, (4) grouped by area of research, with all groups smaller than a threshold of 10 being summarized in one “other” category, (5) grouped by research environment, (6) grouped by research environment, with all groups smaller than a threshold of 10 being summarized in one “other” category. [file peerj-cs-05-240-s002.zip › reproducibility-survey-analysis-byresearchenvironmentthreshold-question-09.png]

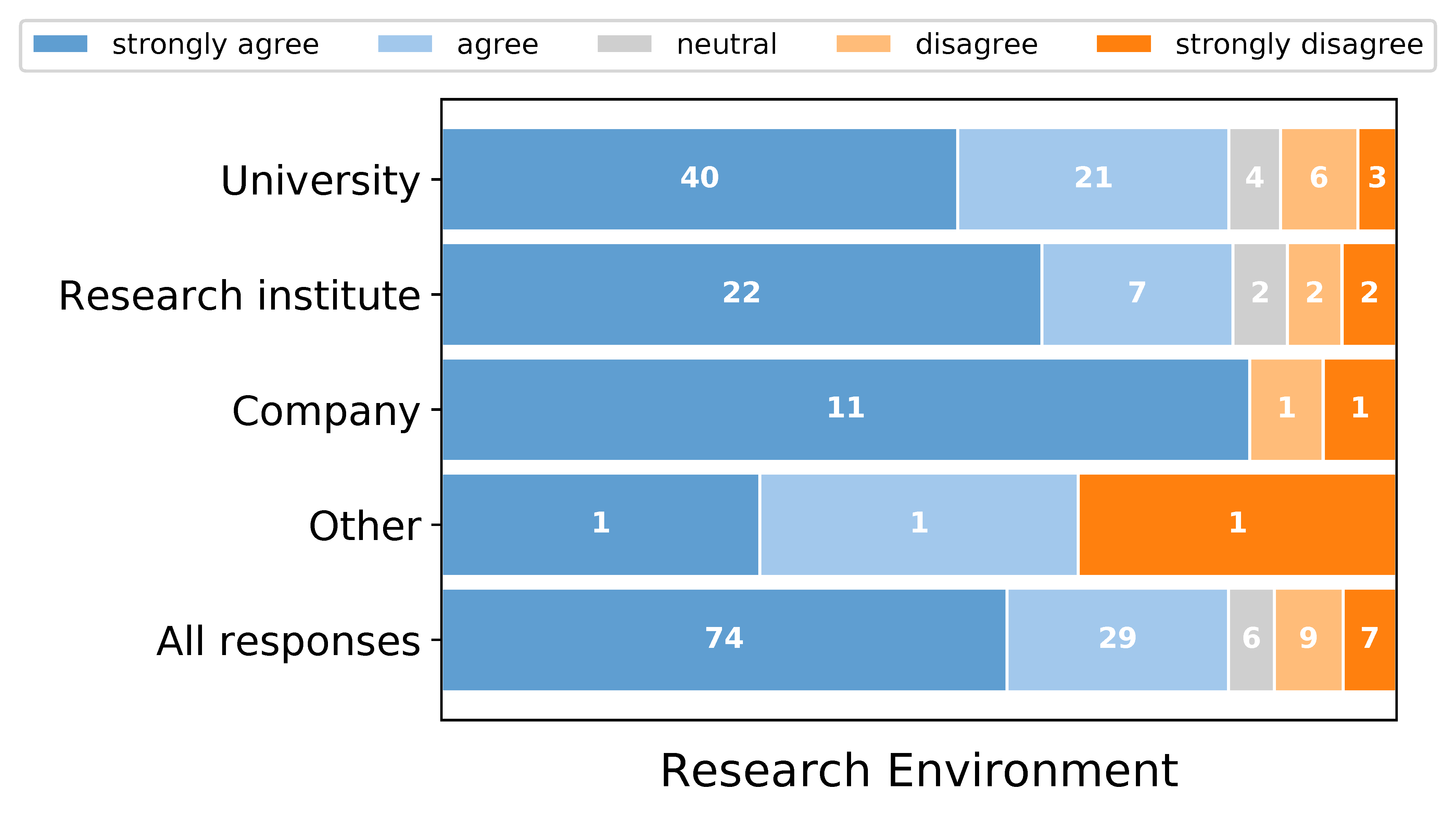

Supplement: Supplemental Information 2 — The answers to each survey question have been evaluated (1) grouped by position, (2) grouped by position, with all groups smaller than a threshold of 10 being summarized in one “other” category, (3) grouped by area of research, (4) grouped by area of research, with all groups smaller than a threshold of 10 being summarized in one “other” category, (5) grouped by research environment, (6) grouped by research environment, with all groups smaller than a threshold of 10 being summarized in one “other” category. [file peerj-cs-05-240-s002.zip › reproducibility-survey-analysis-byresearchenvironmentthreshold-question-10.png]

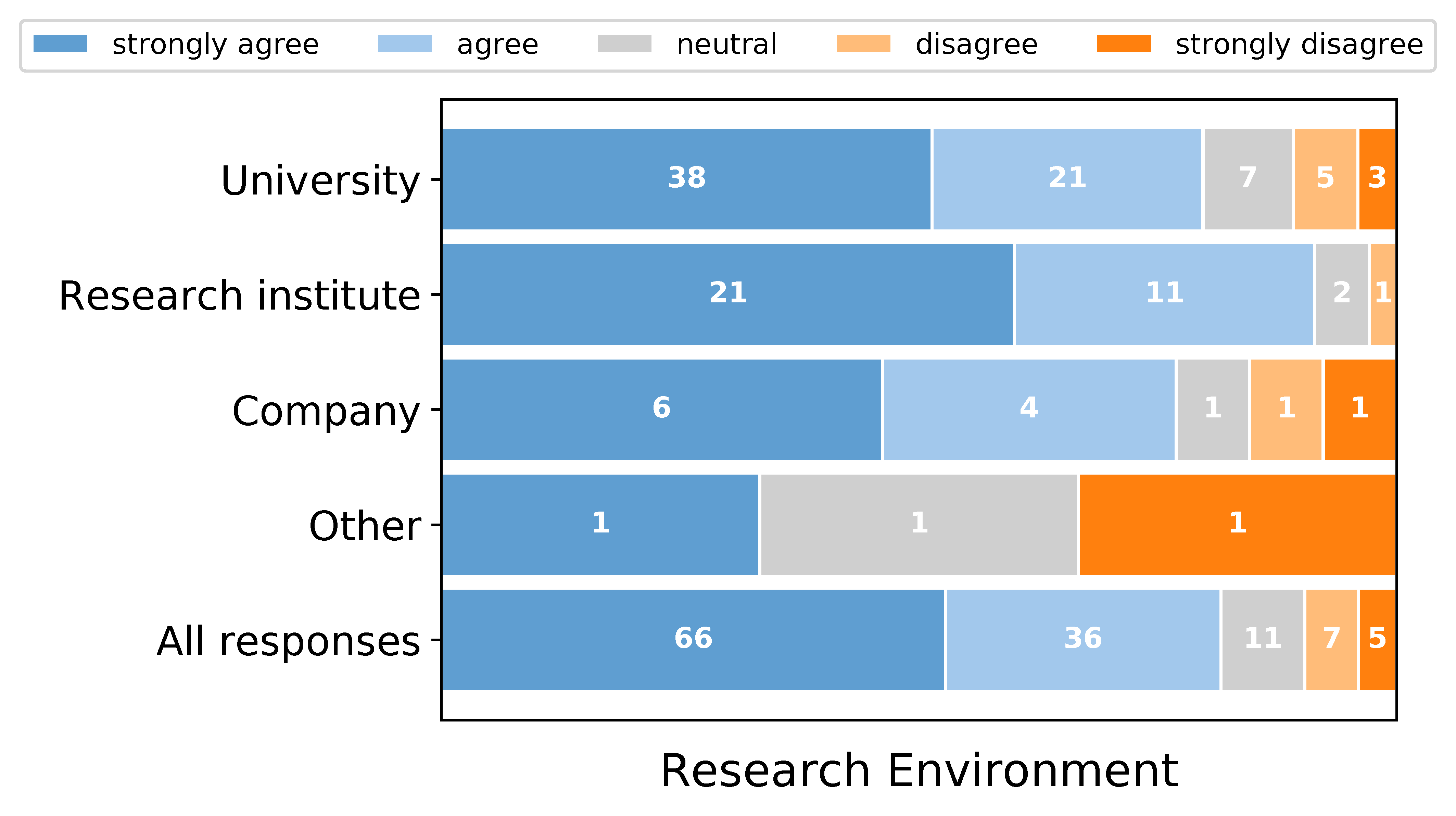

Supplement: Supplemental Information 2 — The answers to each survey question have been evaluated (1) grouped by position, (2) grouped by position, with all groups smaller than a threshold of 10 being summarized in one “other” category, (3) grouped by area of research, (4) grouped by area of research, with all groups smaller than a threshold of 10 being summarized in one “other” category, (5) grouped by research environment, (6) grouped by research environment, with all groups smaller than a threshold of 10 being summarized in one “other” category. [file peerj-cs-05-240-s002.zip › reproducibility-survey-analysis-byresearchenvironmentthreshold-question-11.png]

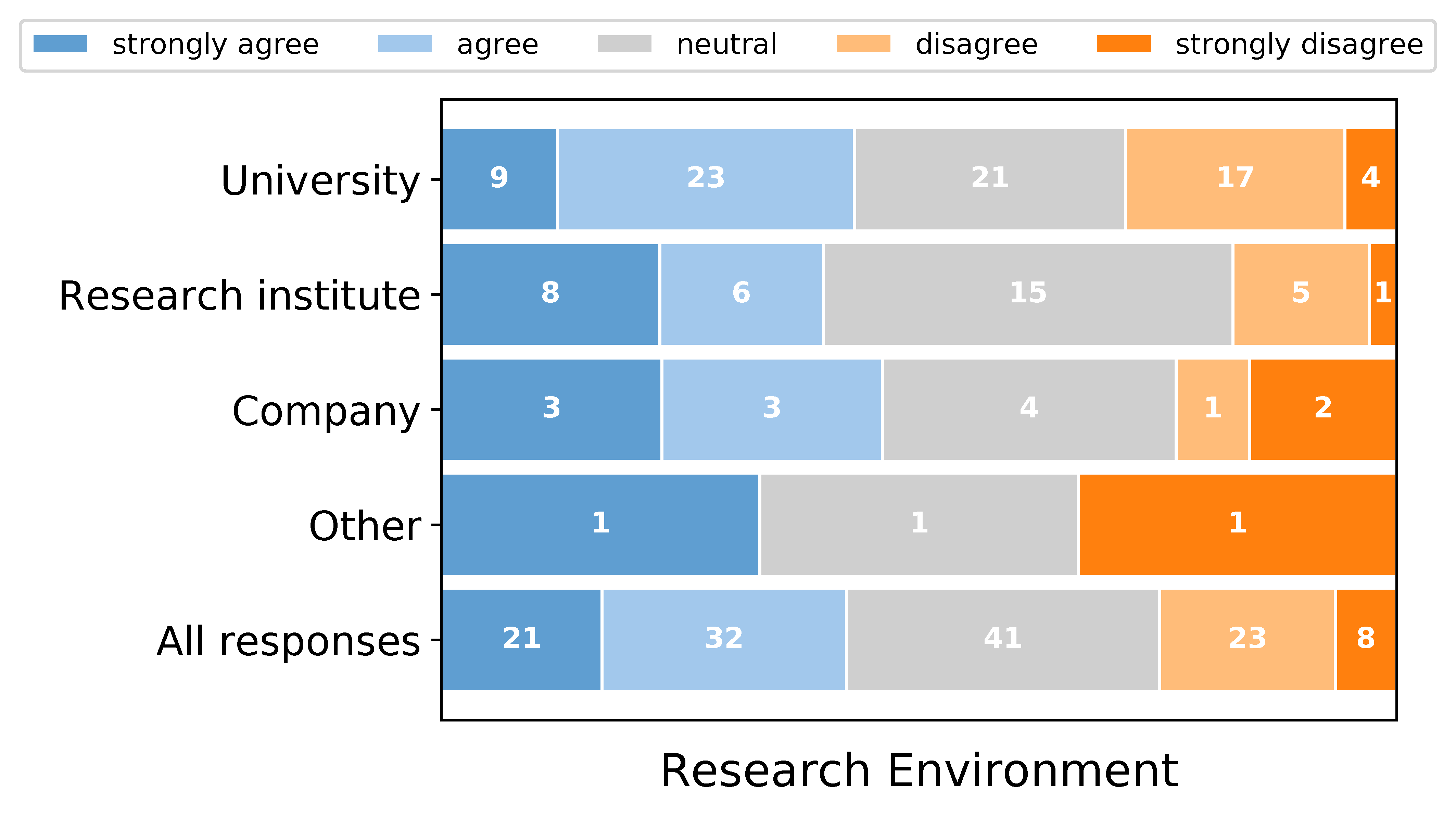

Supplement: Supplemental Information 2 — The answers to each survey question have been evaluated (1) grouped by position, (2) grouped by position, with all groups smaller than a threshold of 10 being summarized in one “other” category, (3) grouped by area of research, (4) grouped by area of research, with all groups smaller than a threshold of 10 being summarized in one “other” category, (5) grouped by research environment, (6) grouped by research environment, with all groups smaller than a threshold of 10 being summarized in one “other” category. [file peerj-cs-05-240-s002.zip › reproducibility-survey-analysis-byresearchenvironmentthreshold-question-12.png]

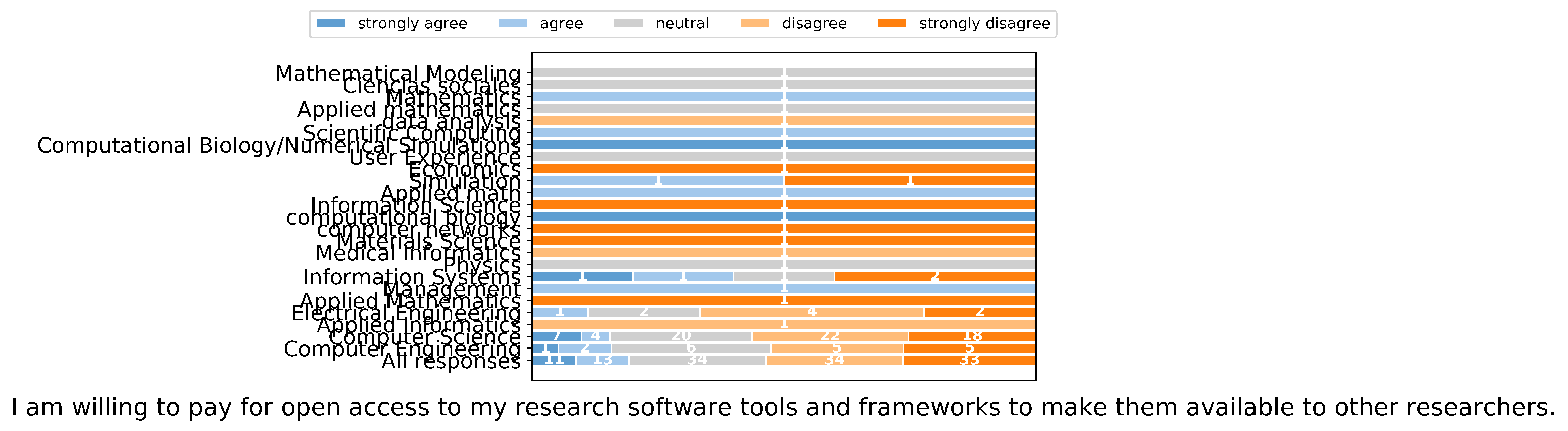

Supplement: Supplemental Information 2 — The answers to each survey question have been evaluated (1) grouped by position, (2) grouped by position, with all groups smaller than a threshold of 10 being summarized in one “other” category, (3) grouped by area of research, (4) grouped by area of research, with all groups smaller than a threshold of 10 being summarized in one “other” category, (5) grouped by research environment, (6) grouped by research environment, with all groups smaller than a threshold of 10 being summarized in one “other” category. [file peerj-cs-05-240-s002.zip › reproducibility-survey-analysis-bythearea-question-01.png]

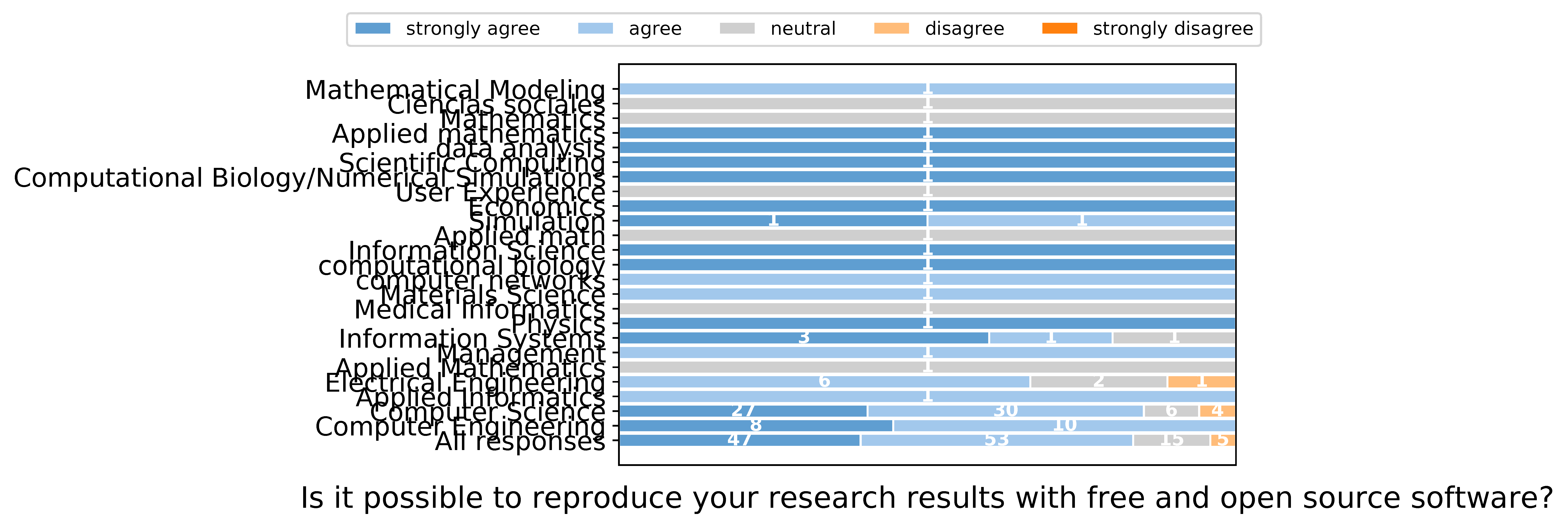

Supplement: Supplemental Information 2 — The answers to each survey question have been evaluated (1) grouped by position, (2) grouped by position, with all groups smaller than a threshold of 10 being summarized in one “other” category, (3) grouped by area of research, (4) grouped by area of research, with all groups smaller than a threshold of 10 being summarized in one “other” category, (5) grouped by research environment, (6) grouped by research environment, with all groups smaller than a threshold of 10 being summarized in one “other” category. [file peerj-cs-05-240-s002.zip › reproducibility-survey-analysis-bythearea-question-02.png]

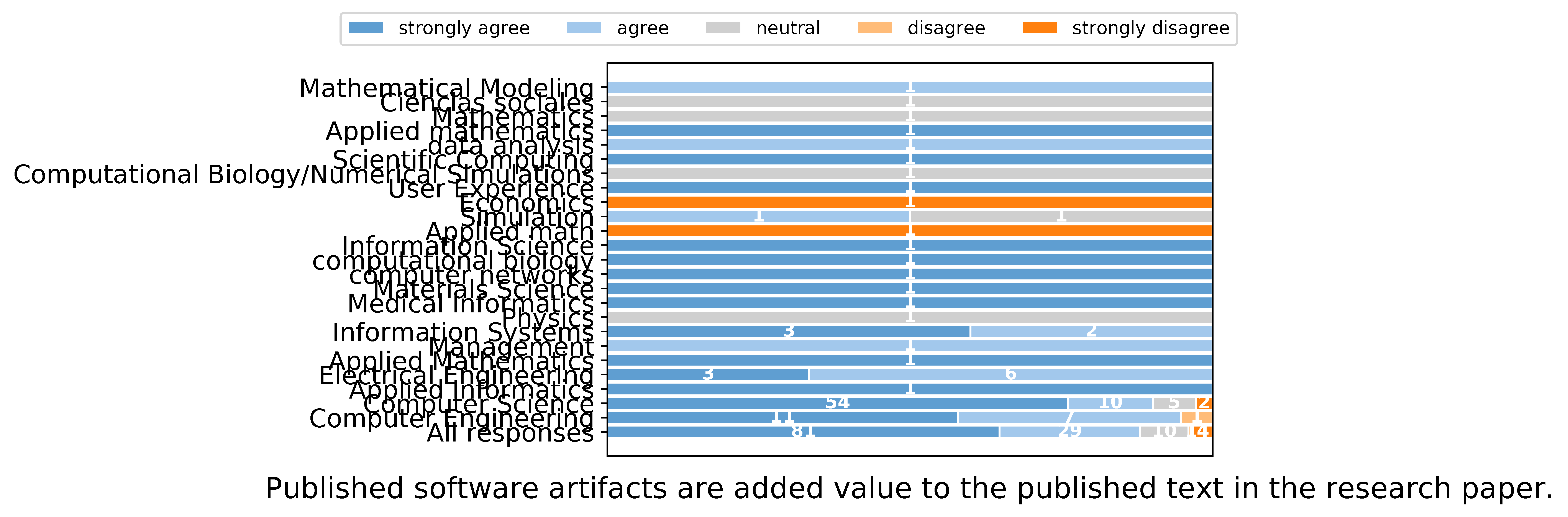

Supplement: Supplemental Information 2 — The answers to each survey question have been evaluated (1) grouped by position, (2) grouped by position, with all groups smaller than a threshold of 10 being summarized in one “other” category, (3) grouped by area of research, (4) grouped by area of research, with all groups smaller than a threshold of 10 being summarized in one “other” category, (5) grouped by research environment, (6) grouped by research environment, with all groups smaller than a threshold of 10 being summarized in one “other” category. [file peerj-cs-05-240-s002.zip › reproducibility-survey-analysis-bythearea-question-03.png]

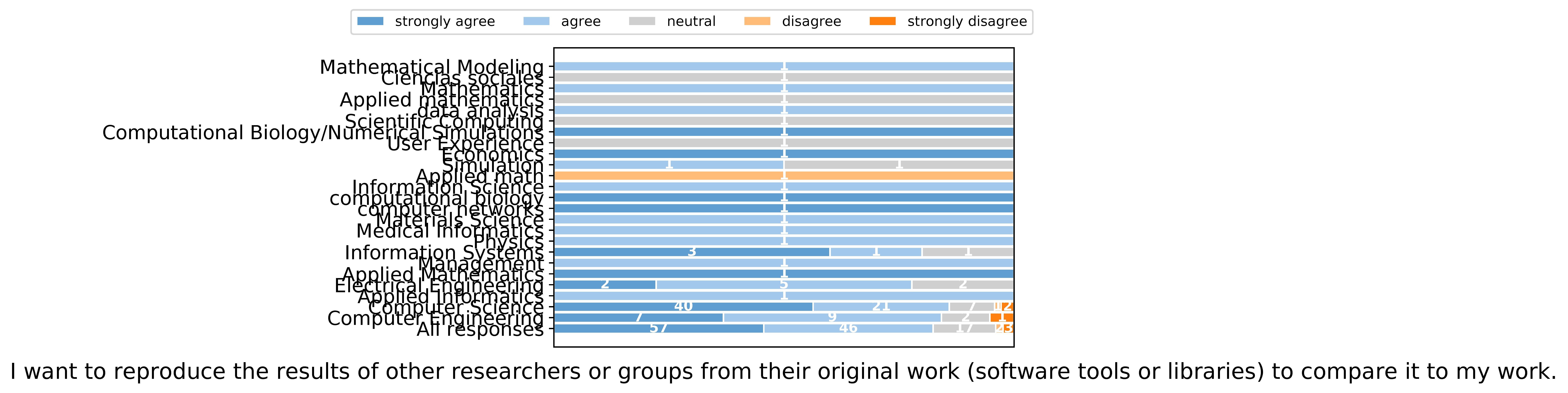

Supplement: Supplemental Information 2 — The answers to each survey question have been evaluated (1) grouped by position, (2) grouped by position, with all groups smaller than a threshold of 10 being summarized in one “other” category, (3) grouped by area of research, (4) grouped by area of research, with all groups smaller than a threshold of 10 being summarized in one “other” category, (5) grouped by research environment, (6) grouped by research environment, with all groups smaller than a threshold of 10 being summarized in one “other” category. [file peerj-cs-05-240-s002.zip › reproducibility-survey-analysis-bythearea-question-04.png]

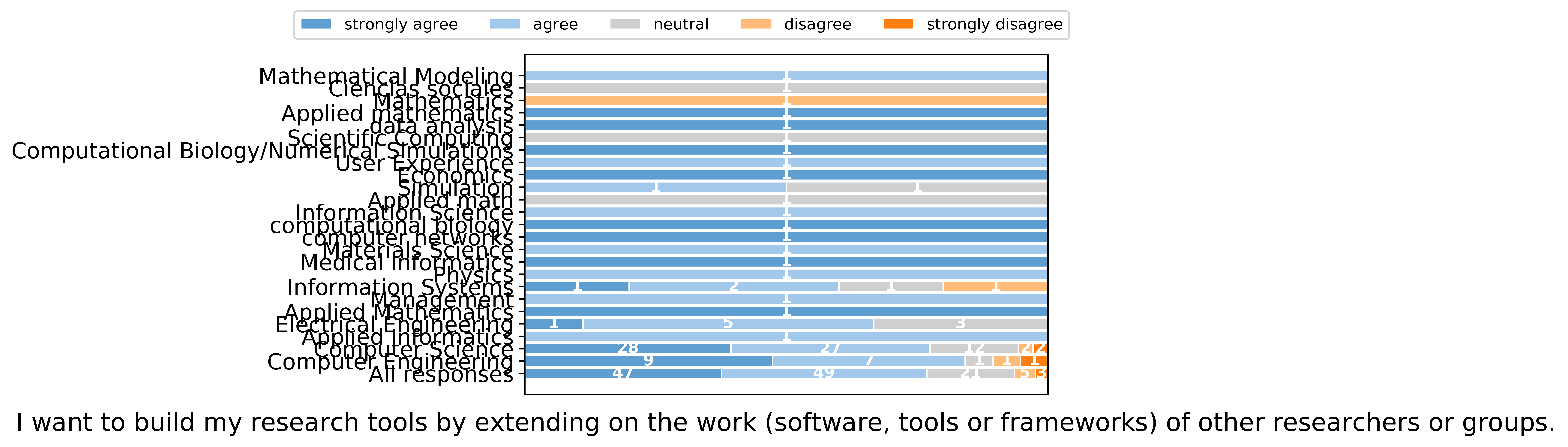

Supplement: Supplemental Information 2 — The answers to each survey question have been evaluated (1) grouped by position, (2) grouped by position, with all groups smaller than a threshold of 10 being summarized in one “other” category, (3) grouped by area of research, (4) grouped by area of research, with all groups smaller than a threshold of 10 being summarized in one “other” category, (5) grouped by research environment, (6) grouped by research environment, with all groups smaller than a threshold of 10 being summarized in one “other” category. [file peerj-cs-05-240-s002.zip › reproducibility-survey-analysis-bythearea-question-05.png]

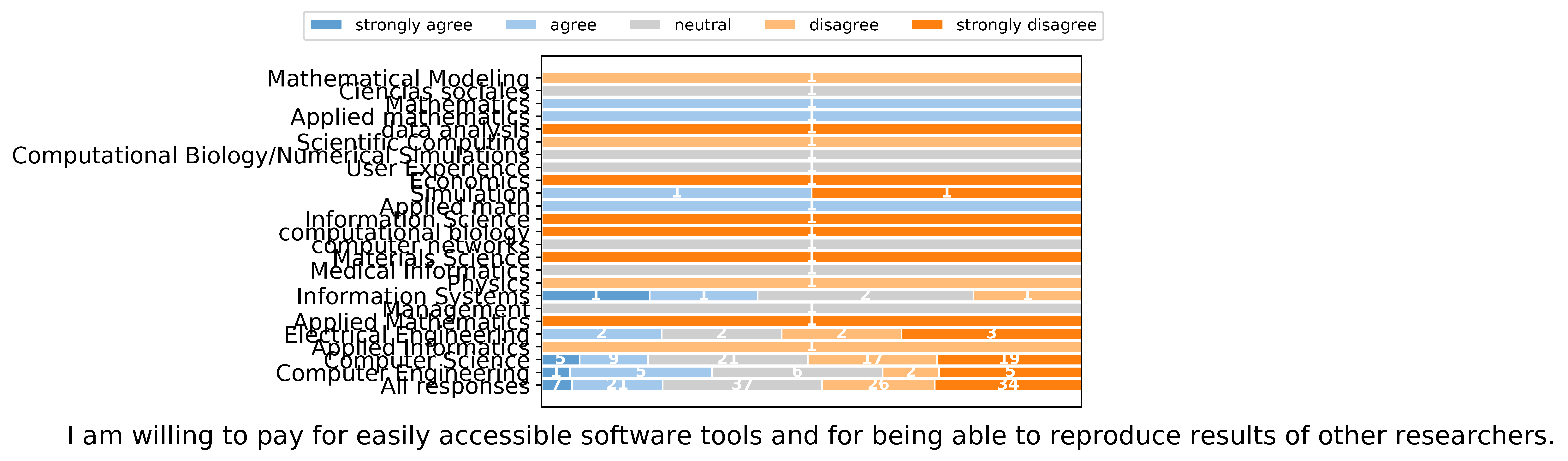

Supplement: Supplemental Information 2 — The answers to each survey question have been evaluated (1) grouped by position, (2) grouped by position, with all groups smaller than a threshold of 10 being summarized in one “other” category, (3) grouped by area of research, (4) grouped by area of research, with all groups smaller than a threshold of 10 being summarized in one “other” category, (5) grouped by research environment, (6) grouped by research environment, with all groups smaller than a threshold of 10 being summarized in one “other” category. [file peerj-cs-05-240-s002.zip › reproducibility-survey-analysis-bythearea-question-06.png]

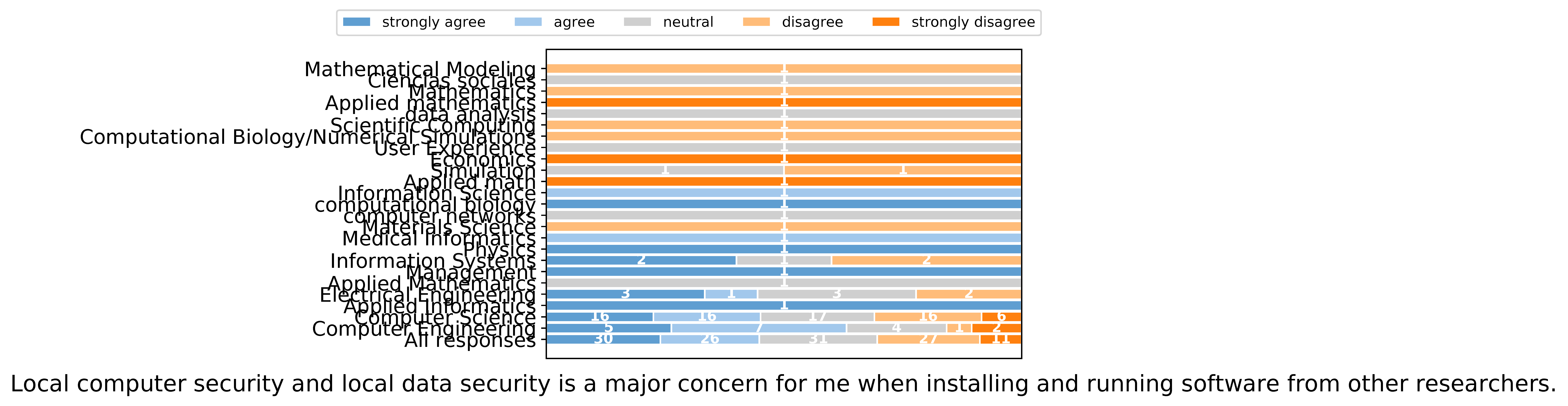

Supplement: Supplemental Information 2 — The answers to each survey question have been evaluated (1) grouped by position, (2) grouped by position, with all groups smaller than a threshold of 10 being summarized in one “other” category, (3) grouped by area of research, (4) grouped by area of research, with all groups smaller than a threshold of 10 being summarized in one “other” category, (5) grouped by research environment, (6) grouped by research environment, with all groups smaller than a threshold of 10 being summarized in one “other” category. [file peerj-cs-05-240-s002.zip › reproducibility-survey-analysis-bythearea-question-07.png]

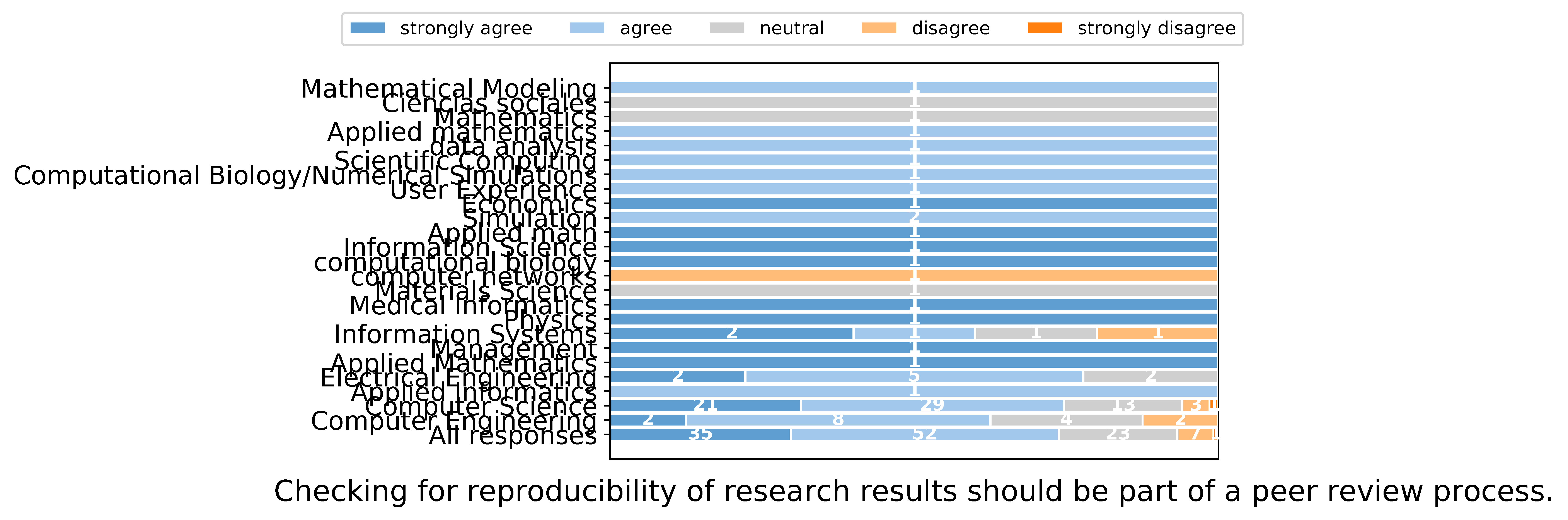

Supplement: Supplemental Information 2 — The answers to each survey question have been evaluated (1) grouped by position, (2) grouped by position, with all groups smaller than a threshold of 10 being summarized in one “other” category, (3) grouped by area of research, (4) grouped by area of research, with all groups smaller than a threshold of 10 being summarized in one “other” category, (5) grouped by research environment, (6) grouped by research environment, with all groups smaller than a threshold of 10 being summarized in one “other” category. [file peerj-cs-05-240-s002.zip › reproducibility-survey-analysis-bythearea-question-08.png]

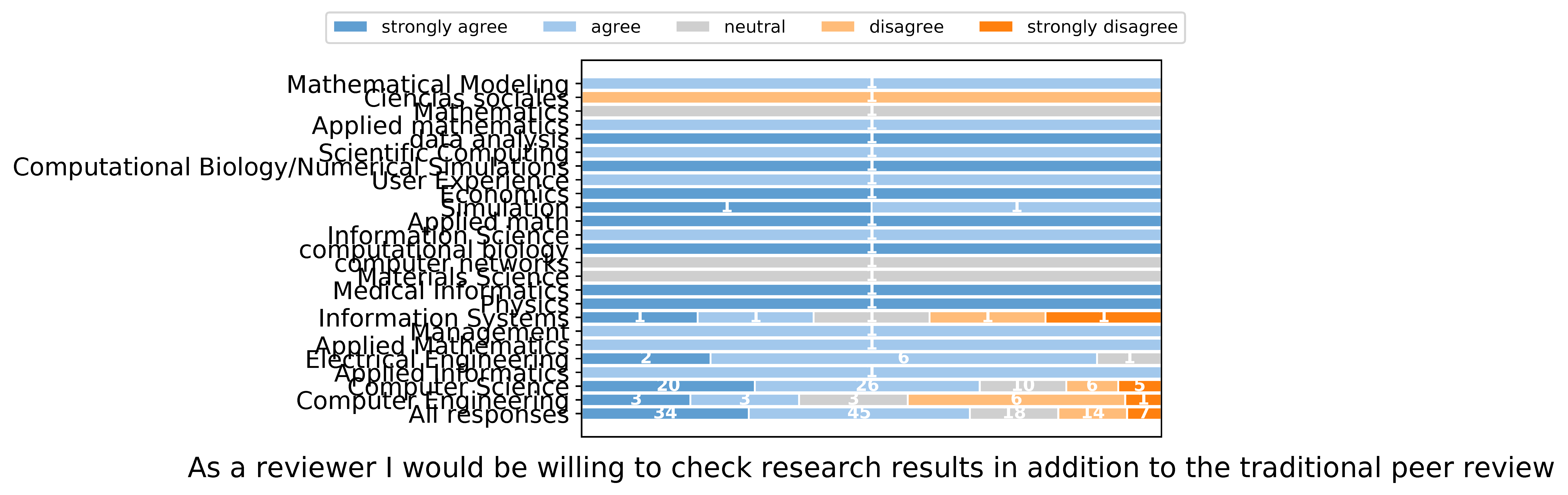

Supplement: Supplemental Information 2 — The answers to each survey question have been evaluated (1) grouped by position, (2) grouped by position, with all groups smaller than a threshold of 10 being summarized in one “other” category, (3) grouped by area of research, (4) grouped by area of research, with all groups smaller than a threshold of 10 being summarized in one “other” category, (5) grouped by research environment, (6) grouped by research environment, with all groups smaller than a threshold of 10 being summarized in one “other” category. [file peerj-cs-05-240-s002.zip › reproducibility-survey-analysis-bythearea-question-09.png]

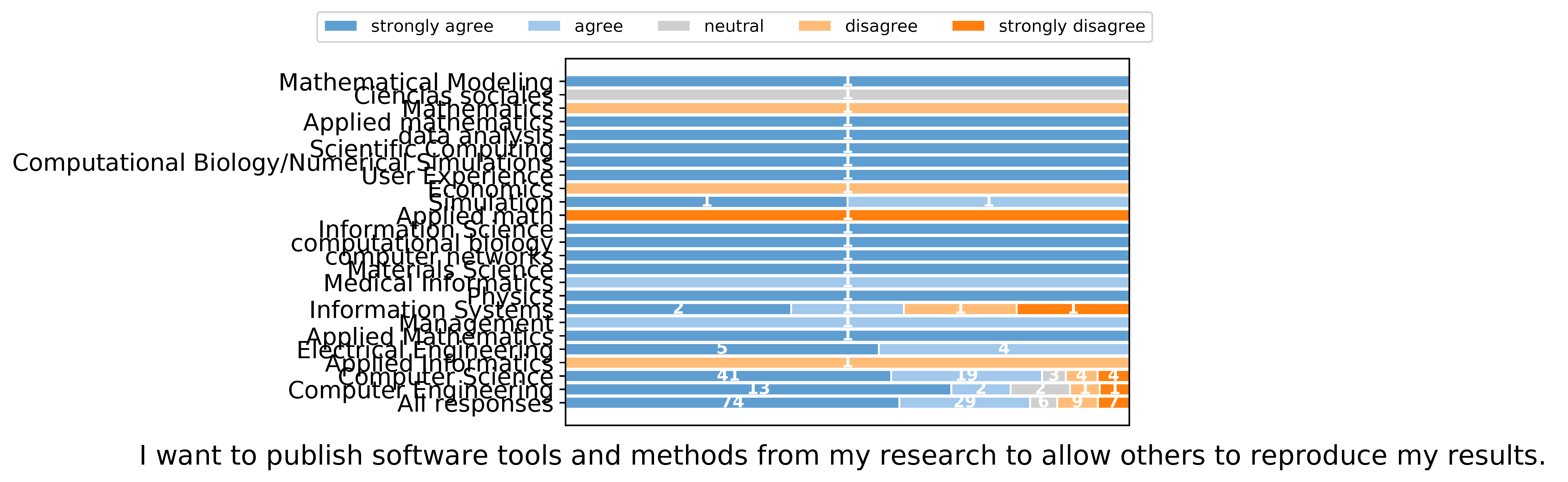

Supplement: Supplemental Information 2 — The answers to each survey question have been evaluated (1) grouped by position, (2) grouped by position, with all groups smaller than a threshold of 10 being summarized in one “other” category, (3) grouped by area of research, (4) grouped by area of research, with all groups smaller than a threshold of 10 being summarized in one “other” category, (5) grouped by research environment, (6) grouped by research environment, with all groups smaller than a threshold of 10 being summarized in one “other” category. [file peerj-cs-05-240-s002.zip › reproducibility-survey-analysis-bythearea-question-10.png]

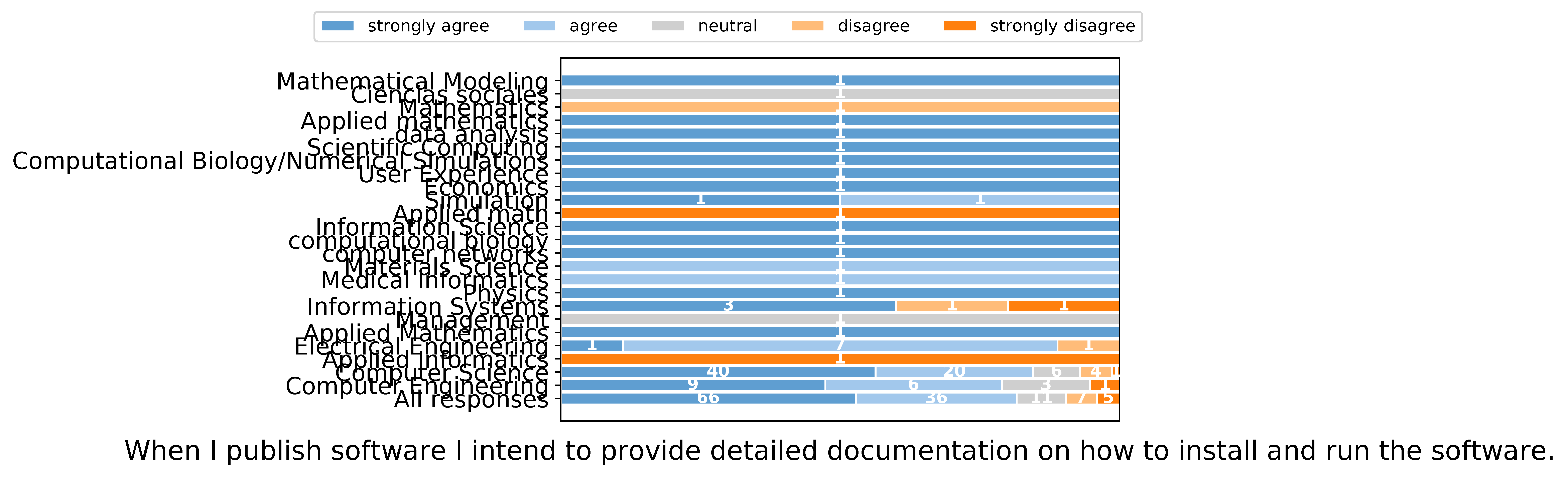

Supplement: Supplemental Information 2 — The answers to each survey question have been evaluated (1) grouped by position, (2) grouped by position, with all groups smaller than a threshold of 10 being summarized in one “other” category, (3) grouped by area of research, (4) grouped by area of research, with all groups smaller than a threshold of 10 being summarized in one “other” category, (5) grouped by research environment, (6) grouped by research environment, with all groups smaller than a threshold of 10 being summarized in one “other” category. [file peerj-cs-05-240-s002.zip › reproducibility-survey-analysis-bythearea-question-11.png]

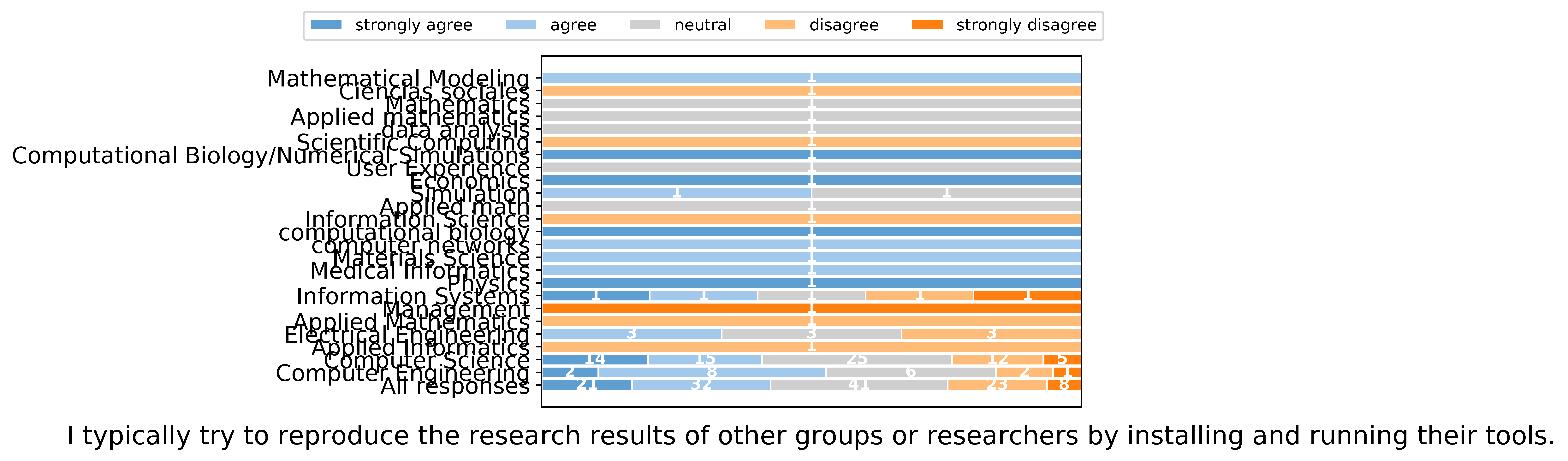

Supplement: Supplemental Information 2 — The answers to each survey question have been evaluated (1) grouped by position, (2) grouped by position, with all groups smaller than a threshold of 10 being summarized in one “other” category, (3) grouped by area of research, (4) grouped by area of research, with all groups smaller than a threshold of 10 being summarized in one “other” category, (5) grouped by research environment, (6) grouped by research environment, with all groups smaller than a threshold of 10 being summarized in one “other” category. [file peerj-cs-05-240-s002.zip › reproducibility-survey-analysis-bythearea-question-12.png]

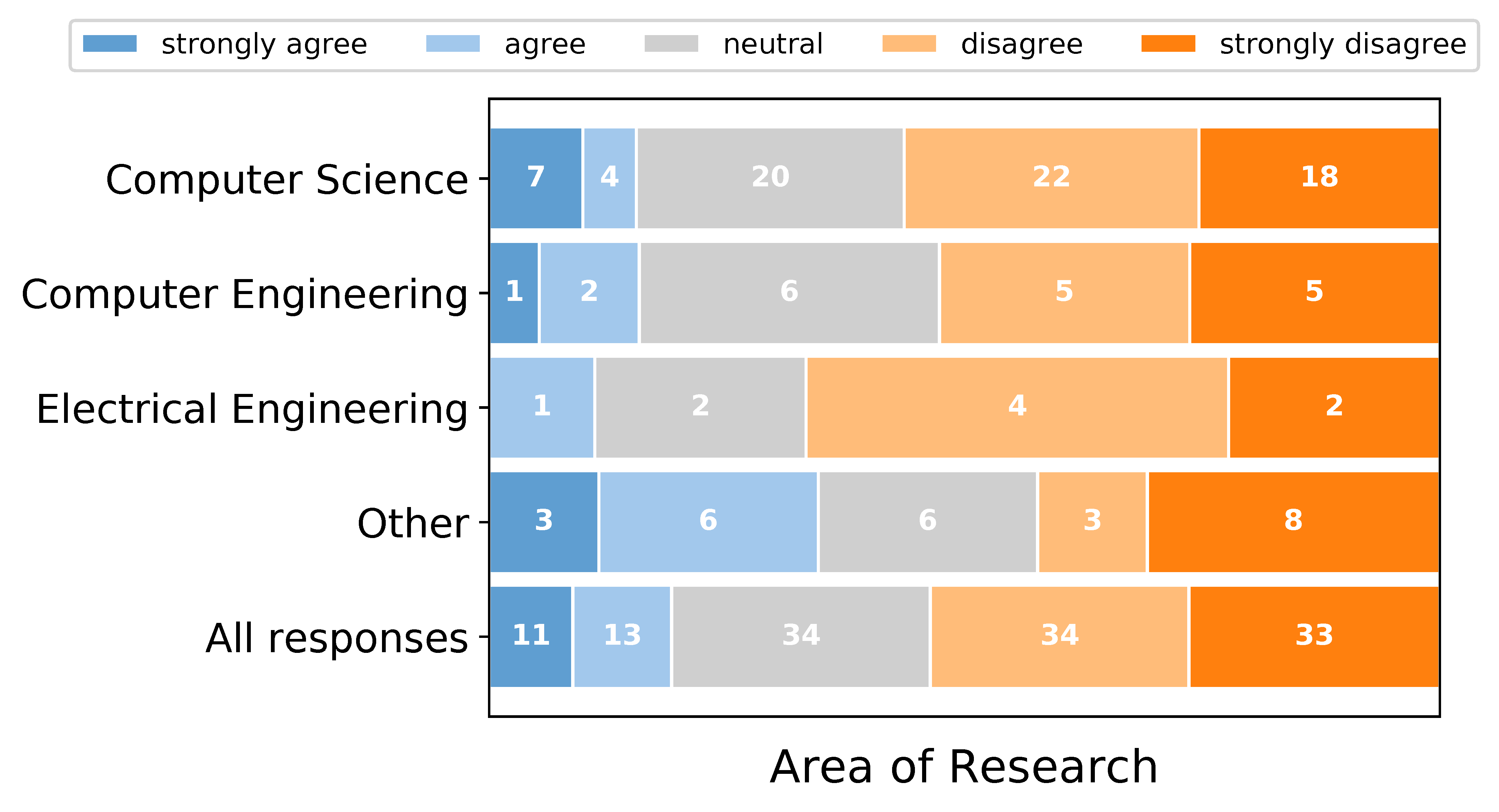

Supplement: Supplemental Information 2 — The answers to each survey question have been evaluated (1) grouped by position, (2) grouped by position, with all groups smaller than a threshold of 10 being summarized in one “other” category, (3) grouped by area of research, (4) grouped by area of research, with all groups smaller than a threshold of 10 being summarized in one “other” category, (5) grouped by research environment, (6) grouped by research environment, with all groups smaller than a threshold of 10 being summarized in one “other” category. [file peerj-cs-05-240-s002.zip › reproducibility-survey-analysis-bytheareathreshold-question-01.png]

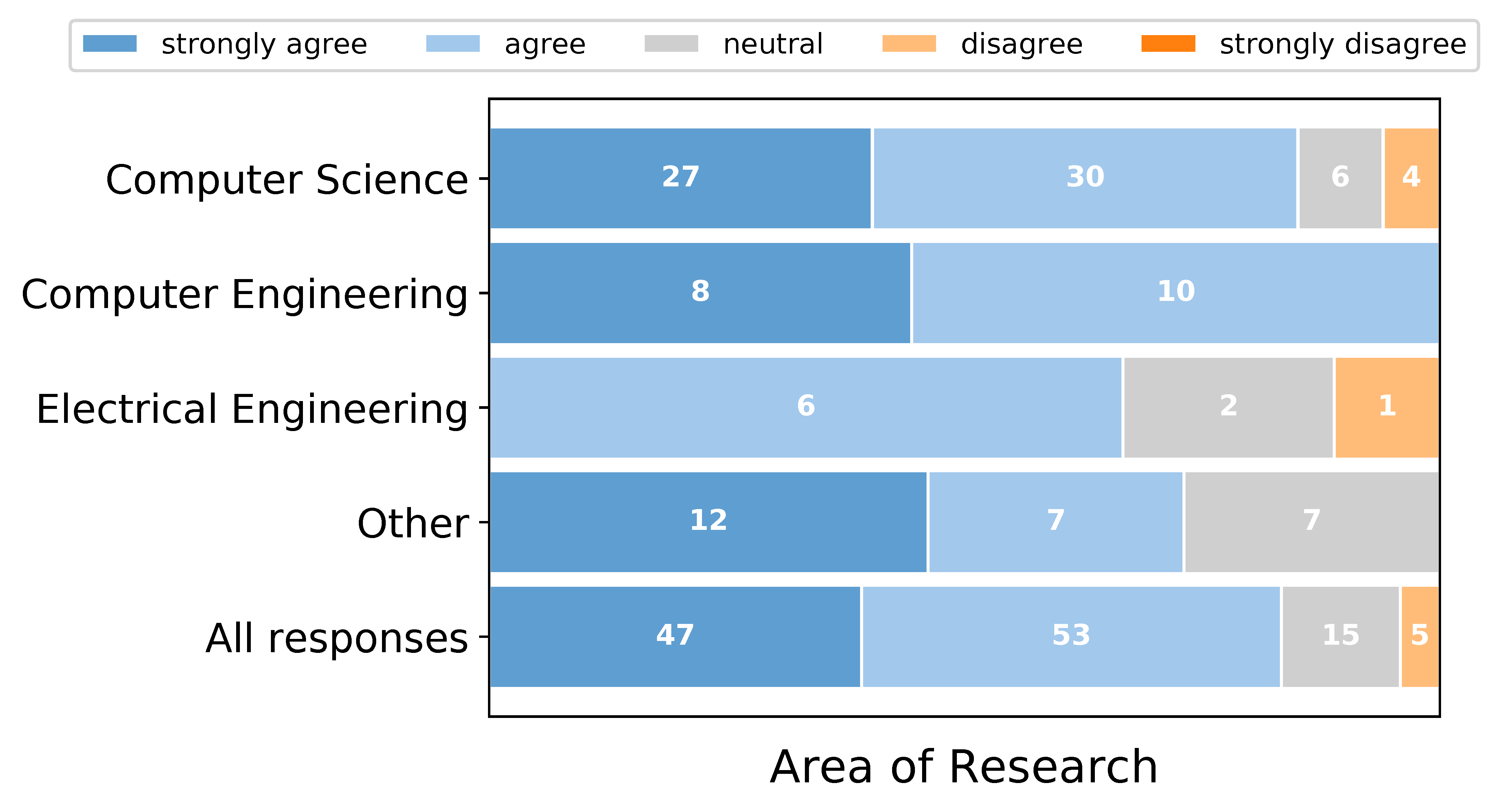

Supplement: Supplemental Information 2 — The answers to each survey question have been evaluated (1) grouped by position, (2) grouped by position, with all groups smaller than a threshold of 10 being summarized in one “other” category, (3) grouped by area of research, (4) grouped by area of research, with all groups smaller than a threshold of 10 being summarized in one “other” category, (5) grouped by research environment, (6) grouped by research environment, with all groups smaller than a threshold of 10 being summarized in one “other” category. [file peerj-cs-05-240-s002.zip › reproducibility-survey-analysis-bytheareathreshold-question-02.png]

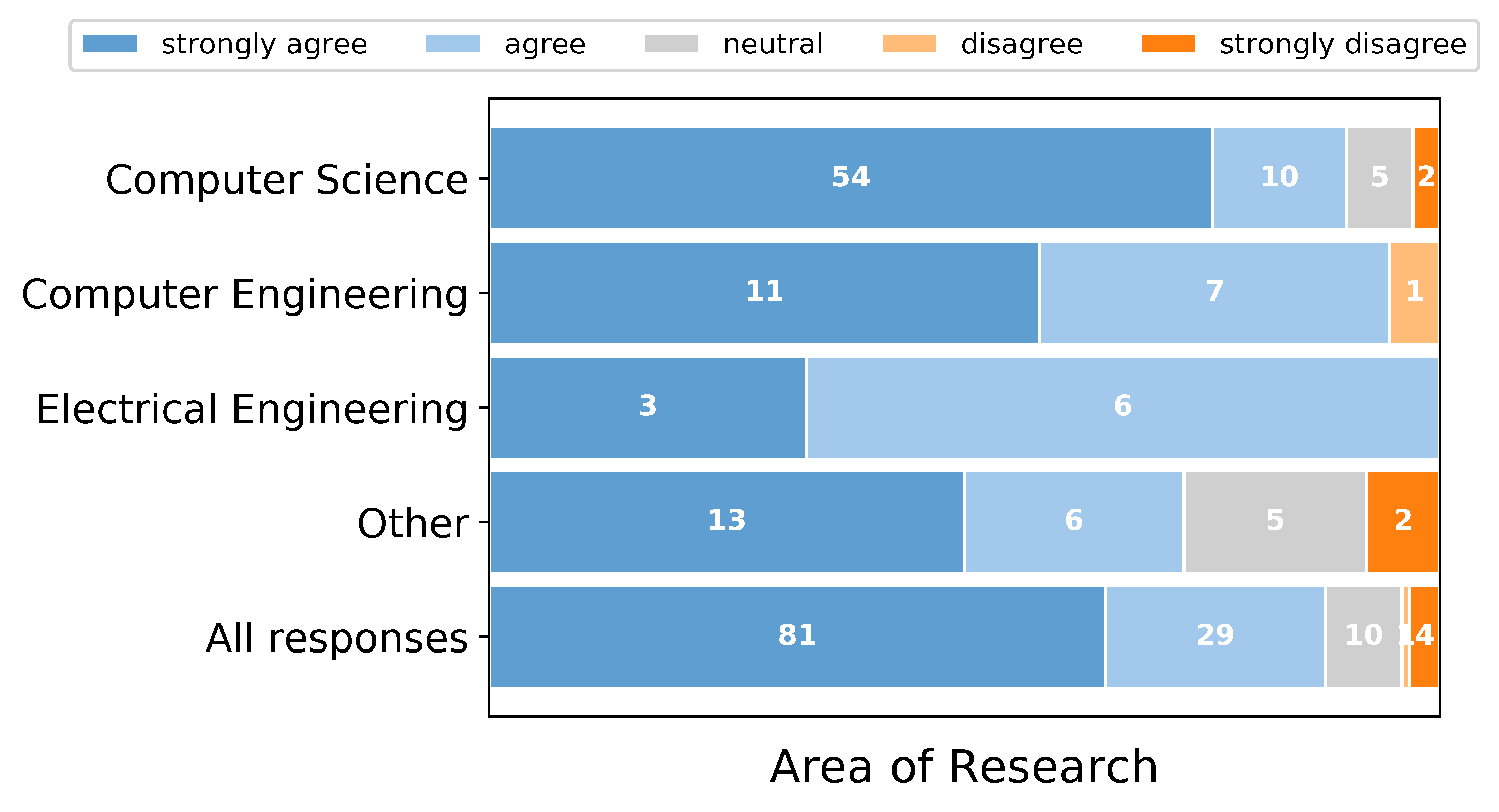

Supplement: Supplemental Information 2 — The answers to each survey question have been evaluated (1) grouped by position, (2) grouped by position, with all groups smaller than a threshold of 10 being summarized in one “other” category, (3) grouped by area of research, (4) grouped by area of research, with all groups smaller than a threshold of 10 being summarized in one “other” category, (5) grouped by research environment, (6) grouped by research environment, with all groups smaller than a threshold of 10 being summarized in one “other” category. [file peerj-cs-05-240-s002.zip › reproducibility-survey-analysis-bytheareathreshold-question-03.png]

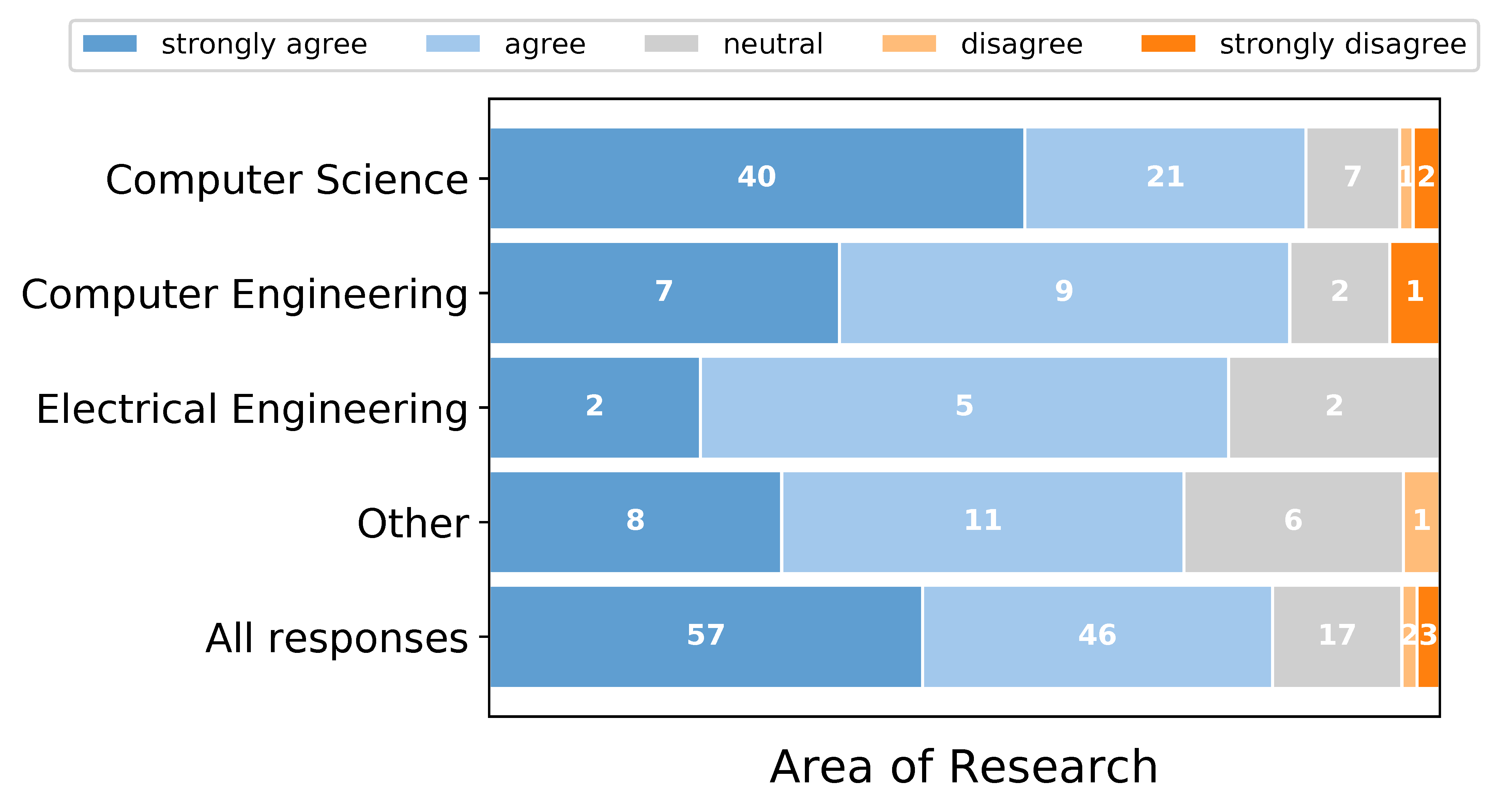

Supplement: Supplemental Information 2 — The answers to each survey question have been evaluated (1) grouped by position, (2) grouped by position, with all groups smaller than a threshold of 10 being summarized in one “other” category, (3) grouped by area of research, (4) grouped by area of research, with all groups smaller than a threshold of 10 being summarized in one “other” category, (5) grouped by research environment, (6) grouped by research environment, with all groups smaller than a threshold of 10 being summarized in one “other” category. [file peerj-cs-05-240-s002.zip › reproducibility-survey-analysis-bytheareathreshold-question-04.png]

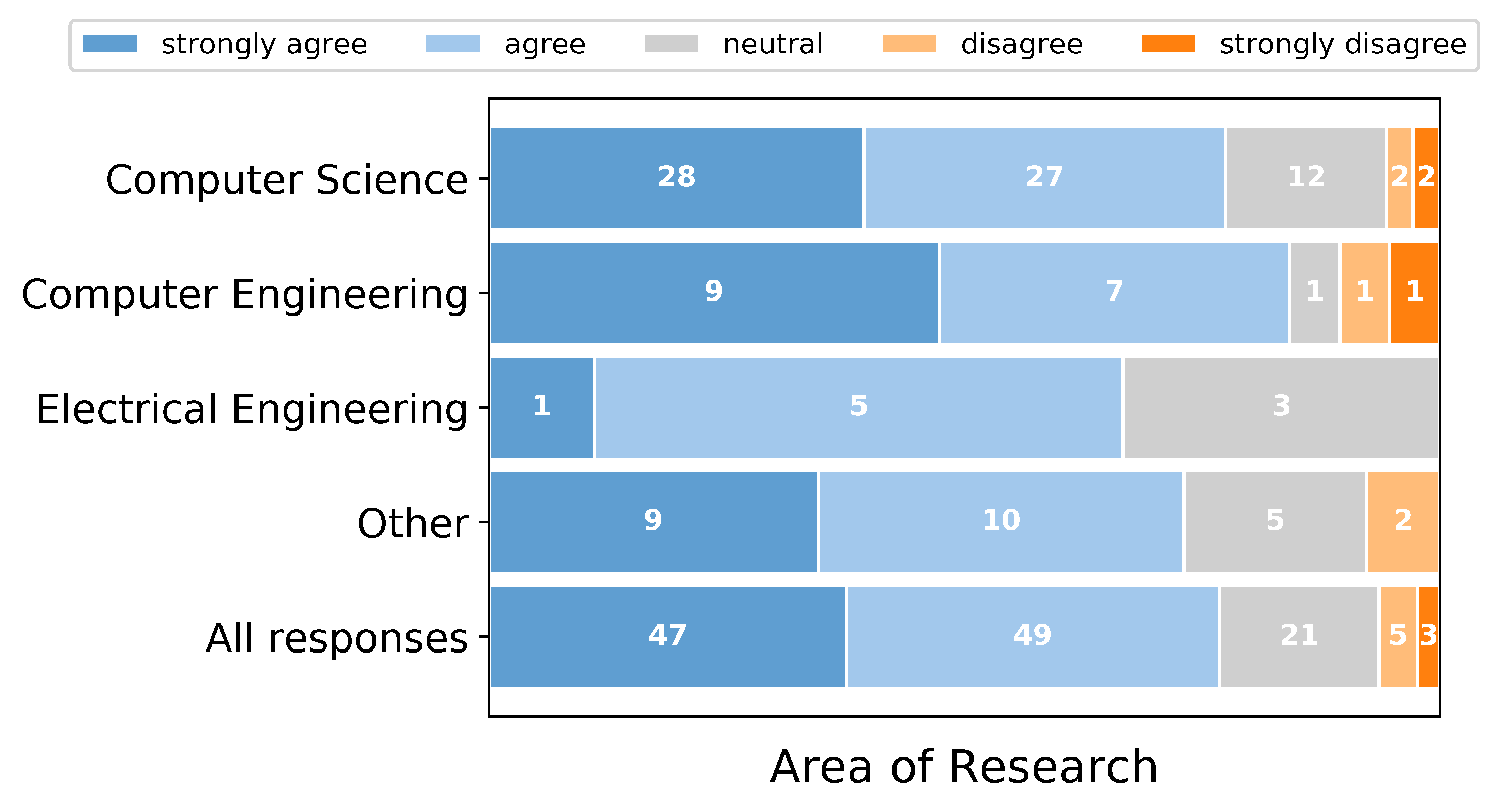

Supplement: Supplemental Information 2 — The answers to each survey question have been evaluated (1) grouped by position, (2) grouped by position, with all groups smaller than a threshold of 10 being summarized in one “other” category, (3) grouped by area of research, (4) grouped by area of research, with all groups smaller than a threshold of 10 being summarized in one “other” category, (5) grouped by research environment, (6) grouped by research environment, with all groups smaller than a threshold of 10 being summarized in one “other” category. [file peerj-cs-05-240-s002.zip › reproducibility-survey-analysis-bytheareathreshold-question-05.png]

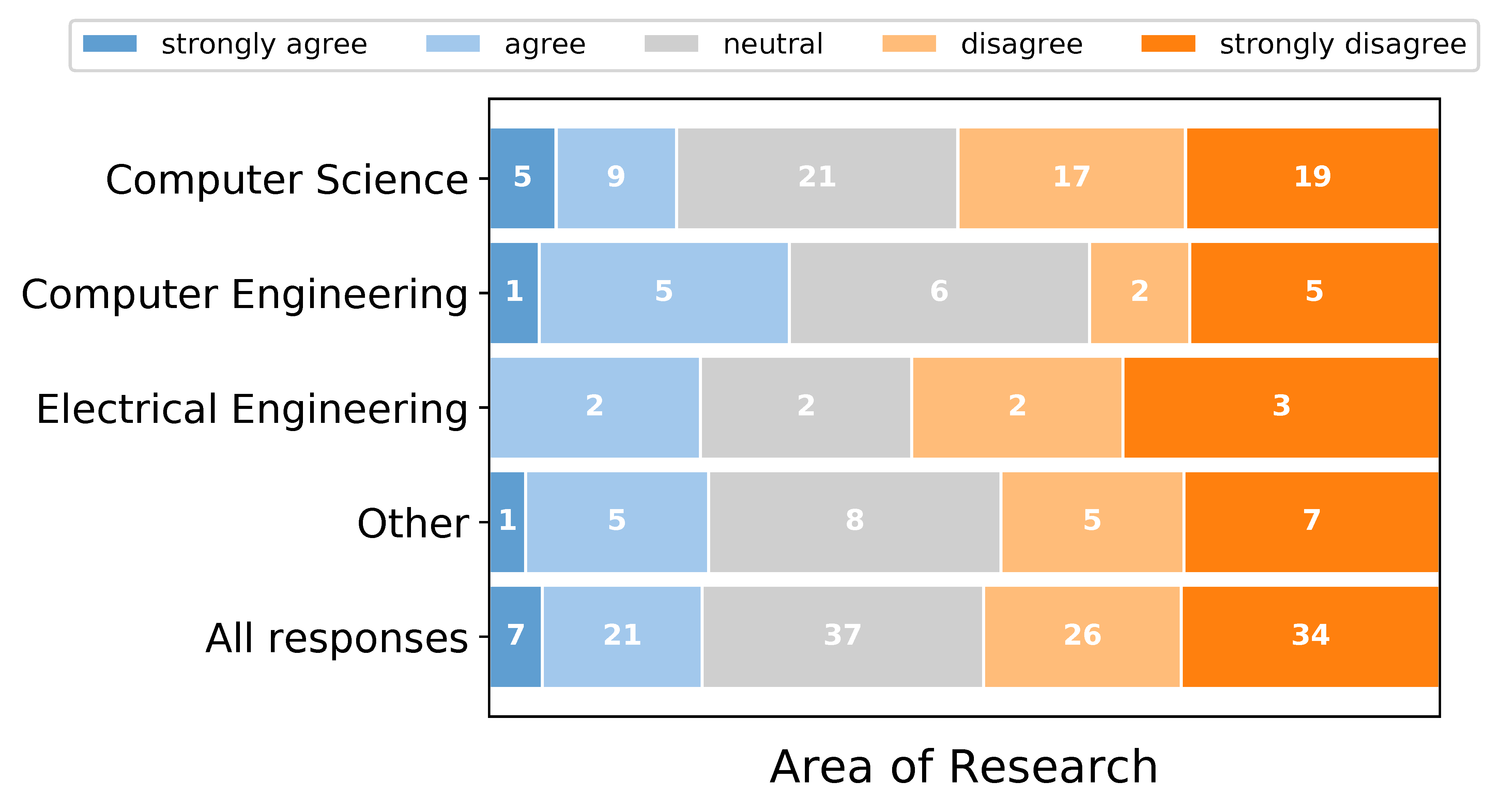

Supplement: Supplemental Information 2 — The answers to each survey question have been evaluated (1) grouped by position, (2) grouped by position, with all groups smaller than a threshold of 10 being summarized in one “other” category, (3) grouped by area of research, (4) grouped by area of research, with all groups smaller than a threshold of 10 being summarized in one “other” category, (5) grouped by research environment, (6) grouped by research environment, with all groups smaller than a threshold of 10 being summarized in one “other” category. [file peerj-cs-05-240-s002.zip › reproducibility-survey-analysis-bytheareathreshold-question-06.png]

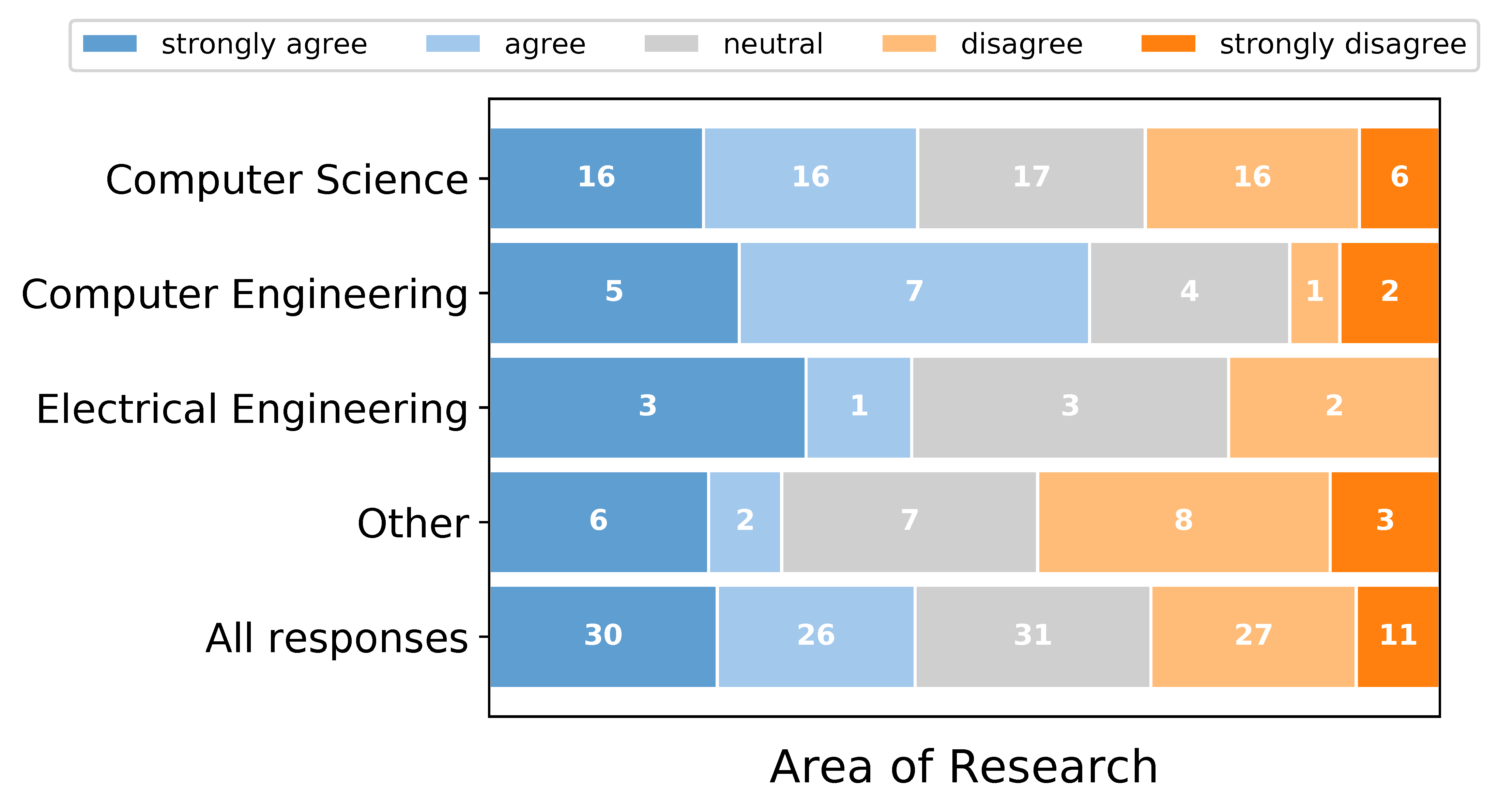

Supplement: Supplemental Information 2 — The answers to each survey question have been evaluated (1) grouped by position, (2) grouped by position, with all groups smaller than a threshold of 10 being summarized in one “other” category, (3) grouped by area of research, (4) grouped by area of research, with all groups smaller than a threshold of 10 being summarized in one “other” category, (5) grouped by research environment, (6) grouped by research environment, with all groups smaller than a threshold of 10 being summarized in one “other” category. [file peerj-cs-05-240-s002.zip › reproducibility-survey-analysis-bytheareathreshold-question-07.png]

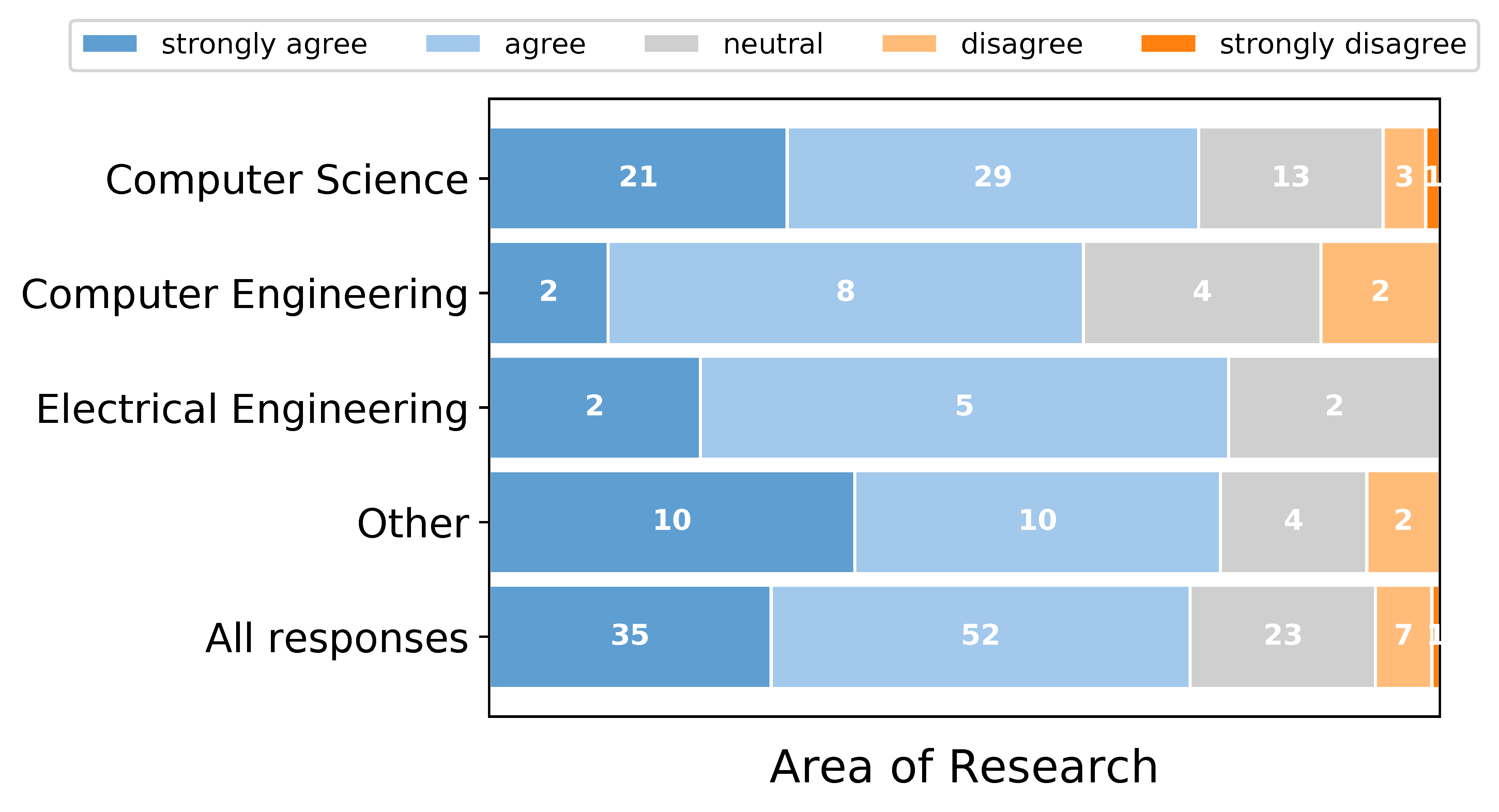

Supplement: Supplemental Information 2 — The answers to each survey question have been evaluated (1) grouped by position, (2) grouped by position, with all groups smaller than a threshold of 10 being summarized in one “other” category, (3) grouped by area of research, (4) grouped by area of research, with all groups smaller than a threshold of 10 being summarized in one “other” category, (5) grouped by research environment, (6) grouped by research environment, with all groups smaller than a threshold of 10 being summarized in one “other” category. [file peerj-cs-05-240-s002.zip › reproducibility-survey-analysis-bytheareathreshold-question-08.png]

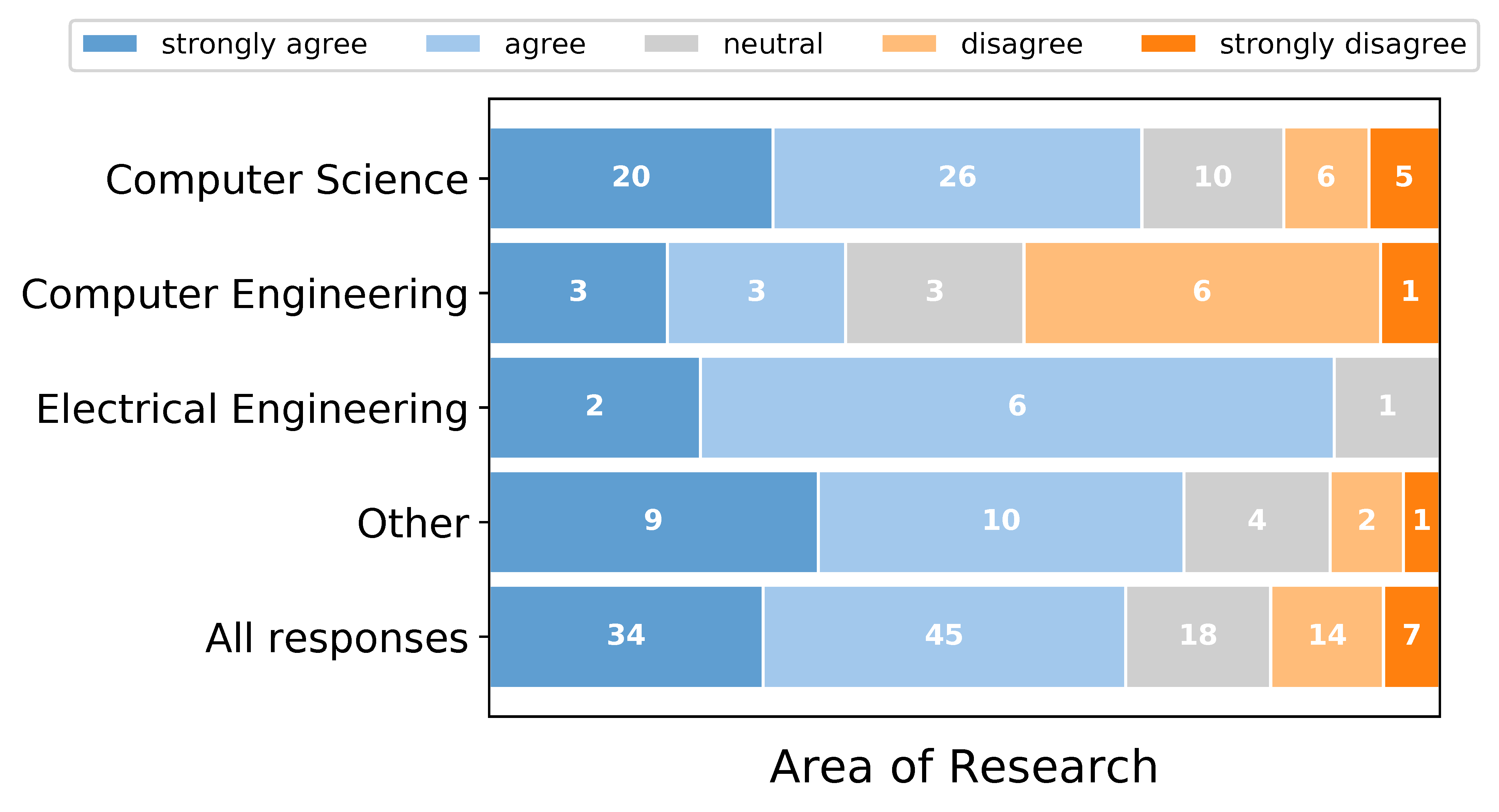

Supplement: Supplemental Information 2 — The answers to each survey question have been evaluated (1) grouped by position, (2) grouped by position, with all groups smaller than a threshold of 10 being summarized in one “other” category, (3) grouped by area of research, (4) grouped by area of research, with all groups smaller than a threshold of 10 being summarized in one “other” category, (5) grouped by research environment, (6) grouped by research environment, with all groups smaller than a threshold of 10 being summarized in one “other” category. [file peerj-cs-05-240-s002.zip › reproducibility-survey-analysis-bytheareathreshold-question-09.png]

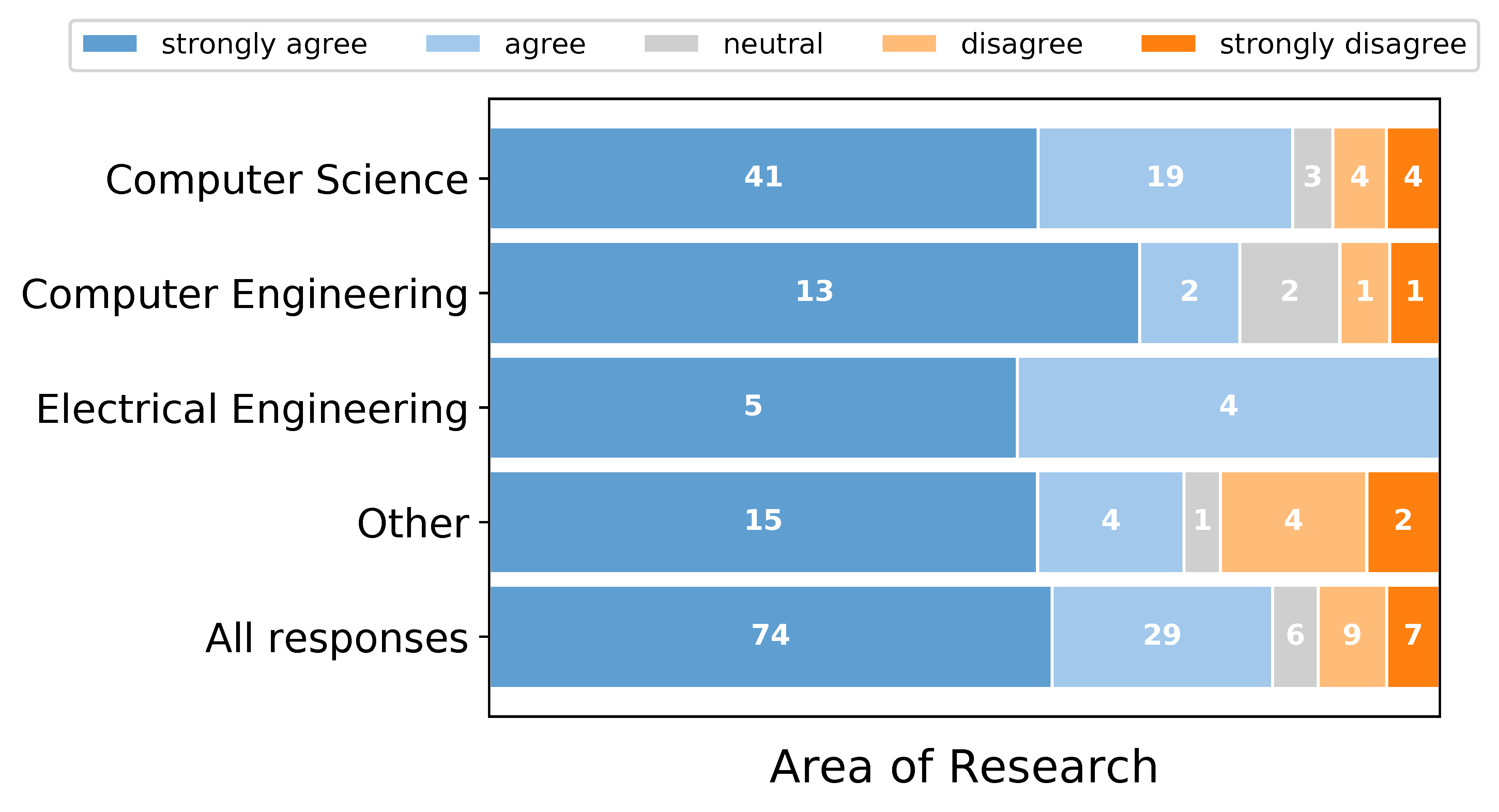

Supplement: Supplemental Information 2 — The answers to each survey question have been evaluated (1) grouped by position, (2) grouped by position, with all groups smaller than a threshold of 10 being summarized in one “other” category, (3) grouped by area of research, (4) grouped by area of research, with all groups smaller than a threshold of 10 being summarized in one “other” category, (5) grouped by research environment, (6) grouped by research environment, with all groups smaller than a threshold of 10 being summarized in one “other” category. [file peerj-cs-05-240-s002.zip › reproducibility-survey-analysis-bytheareathreshold-question-10.png]

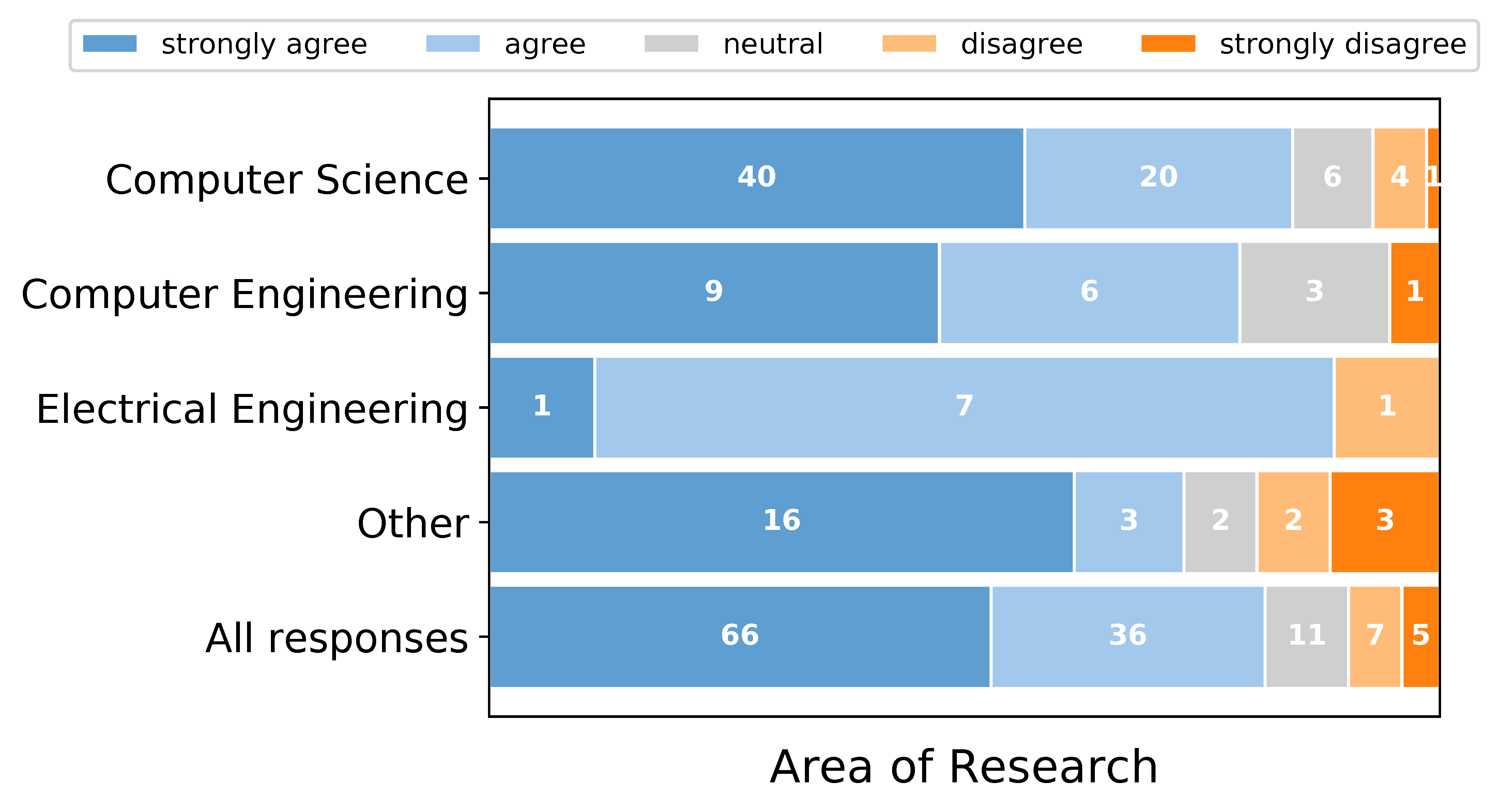

Supplement: Supplemental Information 2 — The answers to each survey question have been evaluated (1) grouped by position, (2) grouped by position, with all groups smaller than a threshold of 10 being summarized in one “other” category, (3) grouped by area of research, (4) grouped by area of research, with all groups smaller than a threshold of 10 being summarized in one “other” category, (5) grouped by research environment, (6) grouped by research environment, with all groups smaller than a threshold of 10 being summarized in one “other” category. [file peerj-cs-05-240-s002.zip › reproducibility-survey-analysis-bytheareathreshold-question-11.png]

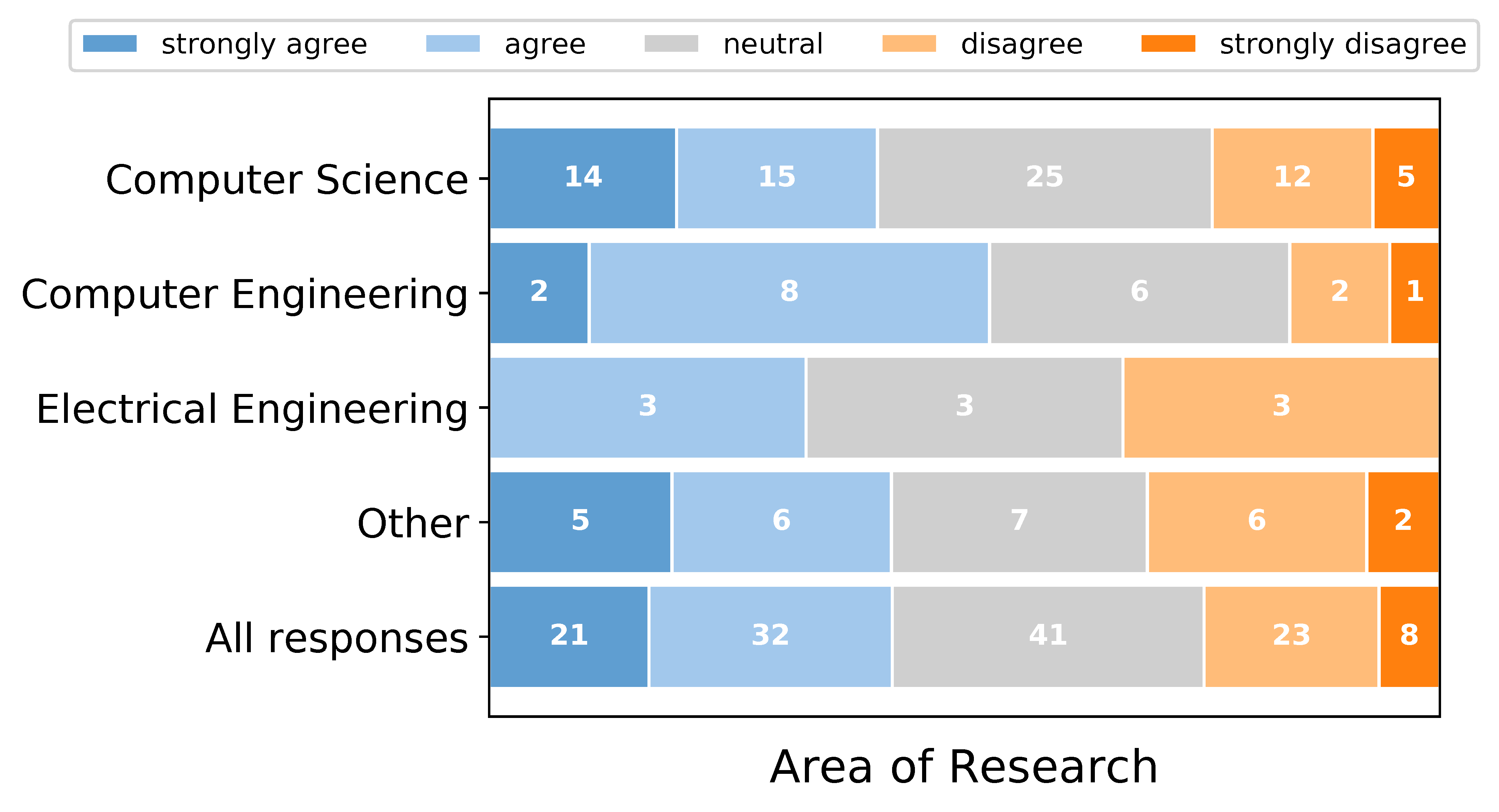

Supplement: Supplemental Information 2 — The answers to each survey question have been evaluated (1) grouped by position, (2) grouped by position, with all groups smaller than a threshold of 10 being summarized in one “other” category, (3) grouped by area of research, (4) grouped by area of research, with all groups smaller than a threshold of 10 being summarized in one “other” category, (5) grouped by research environment, (6) grouped by research environment, with all groups smaller than a threshold of 10 being summarized in one “other” category. [file peerj-cs-05-240-s002.zip › reproducibility-survey-analysis-bytheareathreshold-question-12.png]
